# Selective Hydrogenation of Heteroarenes Using Supported Ruthenium Phosphide Nanoparticle Catalysts

**DOI:** 10.1021/jacs.5c16144

**Published:** 2025-12-23

**Authors:** Hooman Ghazi Zahedi, Jannis Hertel, Bhaskar Paul, Liqun Kang, Jacob Johny, Yufei Wu, Thomas Wiegand, Serena DeBeer, Walter Leitner, Alexis Bordet

**Affiliations:** † 28313 Max Planck Institute for Chemical Energy Conversion, Stiftstrasse 34-36, 45470 Mülheim an der Ruhr, Germany; ‡ Institute of Technical and Macromolecular Chemistry, 9165 RWTH Aachen University, Worringerweg 2, 52074 Aachen, Germany

## Abstract

The selective hydrogenation of heteroarenes, including quinolines, indoles, benzofurans, and benzothiophenes, to the corresponding aromatic compounds is enabled by ruthenium phosphide nanoparticles on imidazolium-based supported ionic liquid phases, Ru_*x*_P_100–*x*_@SILP, as catalysts. The organometallic approach to the synthesis and immobilization of ruthenium phosphide nanoparticles (NPs) allows preparation under mild wet-chemistry conditions and systematic variation of the Ru/P ratio. The novel materials are characterized using various techniques, including electron microscopy and X-ray spectroscopies, and are found to be highly active, selective, and robust for application under batch and continuous flow conditions. The synthetic potential of Ru_50_P_50_@SILP as the most promising catalyst is demonstrated for a wide variety of substrates, enabling the synthesis of valuable drug molecules (e.g., cuspareine and salsolidine) and providing access to useful synthons including isotope labeling for fine chemicals and pharmaceuticals.

## Graphical abstract

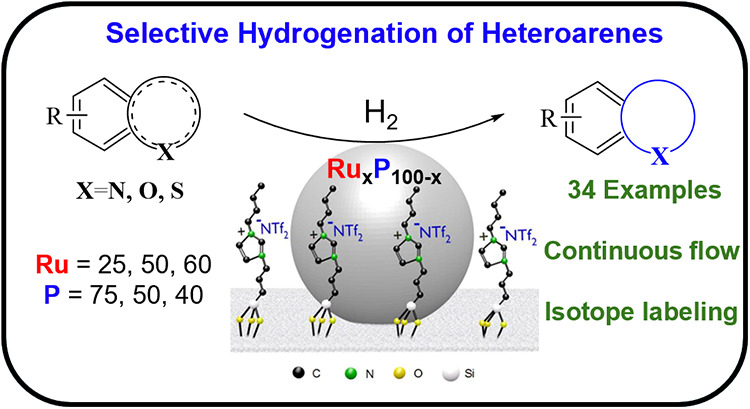

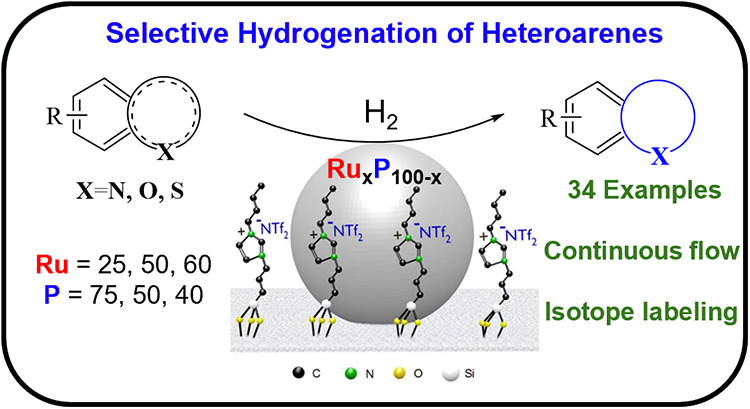

## Introduction

The selective partial hydrogenation of bicyclic heteroaromatic compounds such as quinolines, indoles, benzofurans, and benzothiophenes with molecular hydrogen (H_2_) is a promising approach to access valuable building blocks entering in the synthesis of fine chemicals and pharmaceuticals.^1–3^ In addition, it can potentially enable late-stage modification and derivatization of complex drug molecules.^4–7^ The design of catalysts capable of hydrogenating the heterocycles while leaving the aromatic character of the adjacent carbocycle intact remains a major challenge, however.^8,9^ Over the past decades, significant efforts have been dedicated to the development of homogeneous^1,10–13^ and heterogeneous^14–17^ catalytic systems possessing this ability. In heterogeneous catalysis, strategies to ensure suitable hydrogenation activity while suppressing overreduction include the design of favorable metal–support interactions,^7,17–20^ bimetallic systems,^21–23^ and CO_2_-switchable adaptive catalysts.^24^ These studies mainly focus on the mechanistic understanding of the control factors by using mostly a limited substrate scope of standard substrates. The consistent picture emerging from these studies recognizes the selective adsorption of the heterocycle over the purely aromatic ring as the critical kinetic differentiation path.

Despite this progress, the synthetic potential of this strategy remains largely underexplored, with a combination of robustness and broad synthetic variability defining the main challenges for individual catalysts. We report now the ability of a single catalyst material to selectively hydrogenate a broad range of N- and O-containing heterocycles. In particular, we prepare, characterize, and demonstrate the application of supported ruthenium phosphide nanoparticles for the synthesis of important drug molecules and active pharmaceutical intermediates (APIs) based on quinoline- and indole-type substrates, including application under continuous flow operation and for isotopic labeling (Figure 1).

**Figure 1:**
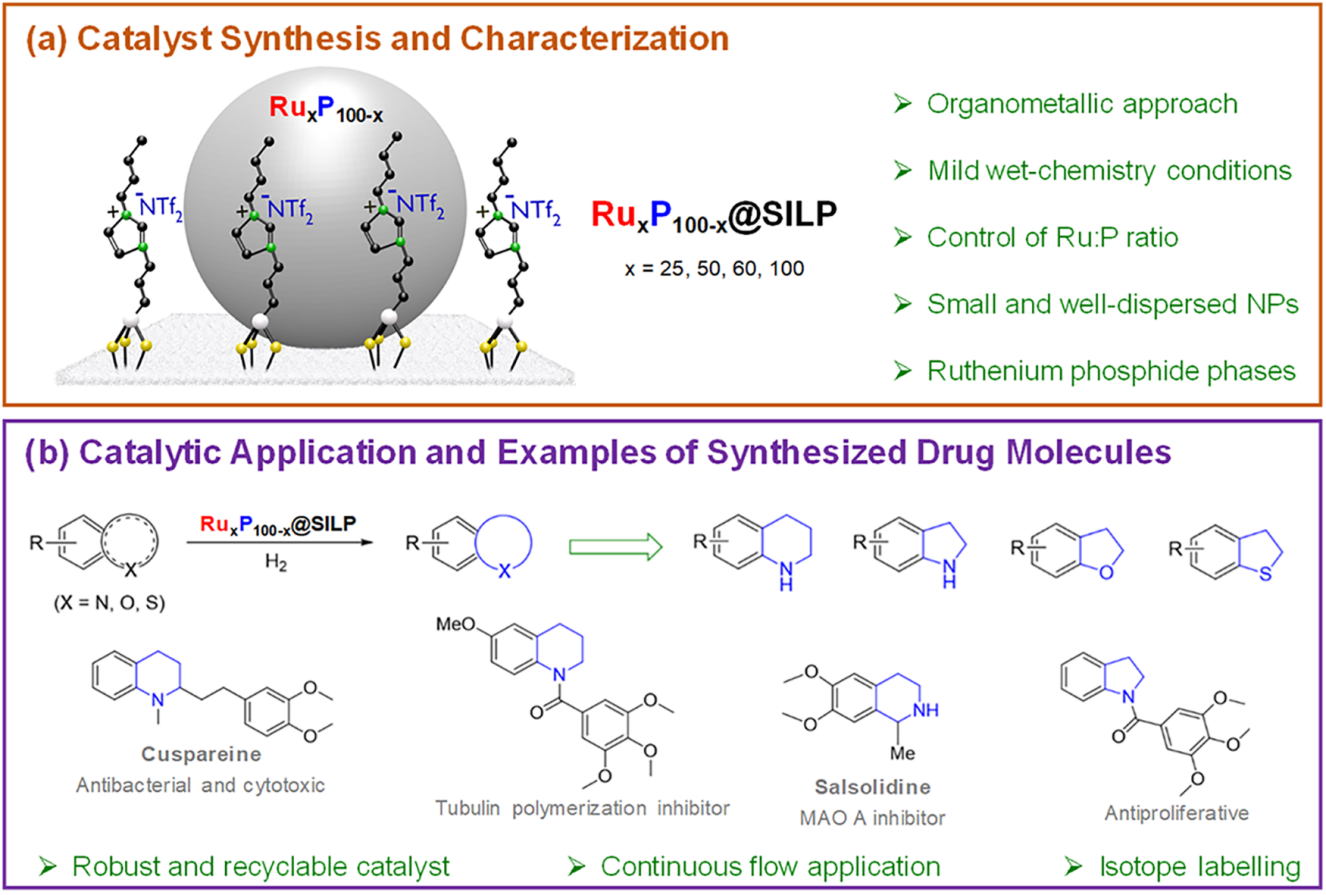
General approach of this study. (a) Schematic representation of supported Ru_*x*_P_100–*x*_ nanoparticles with different Ru/P ratios; (b) selective hydrogenation of bicyclic heteroarenes using Ru_*x*_P_100–*x*_@SILP and examples of pharmaceutical products prepared herein using partially hydrogenated heteroarenes as building blocks.^25–27^ Part of the illustration in (a) was adapted from ref 36. Copyright 2021 American Chemical Society.

## Results and Discussion

### Catalyst Design and Synthesis

We chose ruthenium phosphide nanoparticles (NPs) on molecularly modified supports as promising targets for the catalyst materials. Transition metal phosphide NPs were previously reported for the selective hydrogenation of nitriles (Fe_2_P),^28^ alkynes (Ni_2_P, PdP_2_),^29,30^ and furfural derivatives (Ni_2_P, Co_2_P).^31,32^ Interestingly, in the context of the present study, Shao et al. explored the potential of ruthenium-rich phosphide NPs immobilized on activated carbon (Ru_2_P@AC) as catalysts for the hydrogenation and dehydrogenation of quinolines.^20^ Recently, our group has developed a simple and versatile route enabling the preparation of a wide range of small and well-dispersed transition metal phosphide nanoparticles from commercially available metal and phosphorus precursors under mild reaction conditions (35–55 °C).^33^ In particular, the procedure provides precise size and composition control, allowing systematic variation of the metal-to-phosphorus ratio that is very difficult to achieve by traditional high-temperature syntheses.^31,34,35^ Here, we demonstrate the use of this method for the preparation of tailor-made Ru_*x*_P_100–*x*_ nanoparticles with varying Ru:P ratios immobilized on an imidazolium-based supported ionic liquid phase (Ru_*x*_P_100–*x*_@SILP).

The imidazolium-based SILP was prepared by silanization of the ionic liquid 1-butyl-3-(triethoxysilylpropyl)­imidazolium­[NTf_2_] (NTf_2_ = bis­(trifluoromethanesulfonyl)­imide) on dehydroxylated silica (SiO_2_, 500 °C for 16 h in vacuo), resulting in the formation of the desired SILP with an IL loading of 0.62 mmol_IL_·g_SiO_2__^–1^. This SILP material was demonstrated previously to be suitable for the synthesis and stabilization of various mono- and bimetallic NPs used for hydrogenation^36^ and hydrodeoxygenation reactions,^36,37^ and is thermally stable up to 350 °C.^38^ The synthesis of Ru_*x*_P_100–*x*_@SILP materials was achieved by the reaction of [RuCl_2_(cymene)] with P­(SiMe_3_)_3_ directly in the presence of SILP in mesitylene at 60 °C for 16 h (Figure 2). The release of trimethylsilyl chloride (SiClMe_3_) as a byproduct acts as a driving force for the formation of ruthenium phosphide phases under these conditions, owing to the superior strength of the Si–Cl bond (Bond Dissociation Energy (BDE) = 490.1 kJ mol^–1^) as compared to that of Ru–Cl (BDE = 337.6 kJ mol^–1^) and P–Si (BDE = 363.6 kJ mol^–1^) bonds.^33^ The Ru/P ratio was controlled simply by adjusting the amounts of Ru and P precursors introduced. The use of the SILP support material was found crucial to prevent the uncontrolled decomposition of P­(SiMe_3_)_3_ at the SiO_2_ surface during the reaction (Figure S1). The approach allowed the formation of well-defined NPs with tunable composition (*x* = 25, 50, 60, 100). The reference Ru_100_@SILP material was prepared by reduction of [bis­(2-methylallyl)­(1,5-cyclooctadiene)­Ru] under H_2_ following a literature procedure.^39^

**Figure 2:**
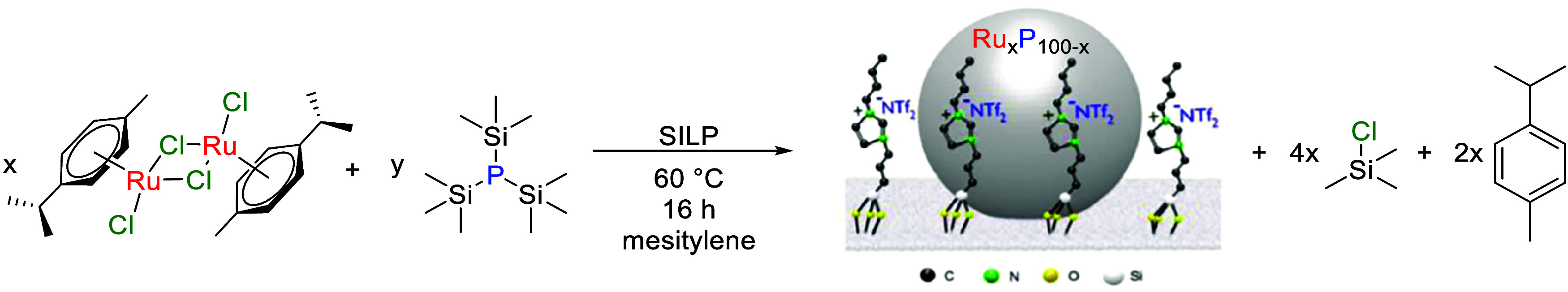
Synthesis of Ru_*x*_P_100–*x*_@SILP materials with controlled Ru/P ratios.

### Catalyst Characterization

A Brunauer–Emmett–Teller (BET) surface area of 309 m^2^·g^–1^ was determined via N_2_ physisorption experiments for the SILP materials, lower than that of the pristine dehydroxylated silica (378 m^2^·g^–1^) due to the presence of the chemically bonded ionic liquid at the SiO_2_ surface. Ru loadings and Ru:P ratios on Ru_*x*_P_100–*x*_@SILP materials determined by inductively coupled plasma optical emission spectroscopy (ICP-OES) analysis and scanning electron microscopy with energy-dispersive X-ray spectroscopy (SEM-EDX) were found consistent with theoretical values (e.g., 4.7 wt % Ru loading and Ru/P ratio of 54:46 for Ru_50_P_50_@SILP, Table S1). Scanning transmission electron microscopy (STEM) revealed the presence of small and well-dispersed nanoparticles with a particle size around 2 nm for all three catalysts, without any observable correlation between particle size and the Ru/P ratio (Table S1). Elemental mapping via high-angle annular dark-field energy-dispersive X-ray spectroscopy (STEM-HAADF-EDX) showed a good overlap of Ru and P signals within the NPs (Figures 3 and S2–S4).

**Figure 3:**
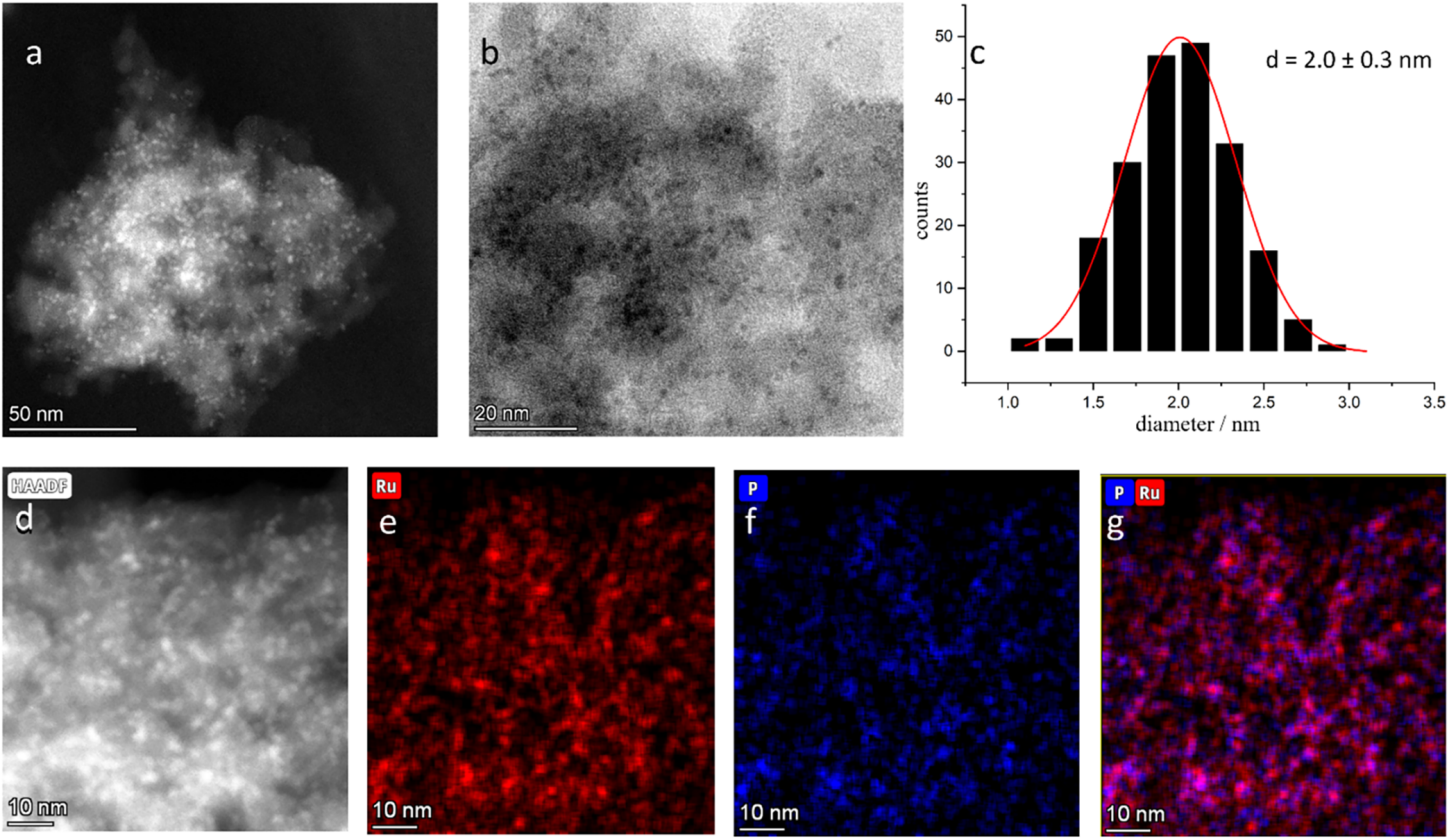
Electron microscopy analysis of Ru_50_P_50_@SILP. (a) STEM-HAADF image; (b) STEM bright-field image; (c) NPs size distribution histogram; (d) STEM-HAADF image; (e) STEM-HAADF-EDX elemental mapping of Ru (Lα); (f) STEM-HAADF-EDX elemental mapping of P (Kα); (g) overlay.

High-resolution N 1s and F 1s X-ray photoelectron spectra of Ru_60_P_40_@SILP (X-ray photoelectron spectroscopy (XPS), Figure 4a,b) as well as solid-state NMR spectroscopy (^1^H–^13^C cross-polarization (CP)-MAS, Figure S5) showed expected characteristic signals of the chemisorbed IL structure, indicating that NPs immobilization did not affect the SILP molecular structure, consistent with recent observations.^40^

**Figure 4:**
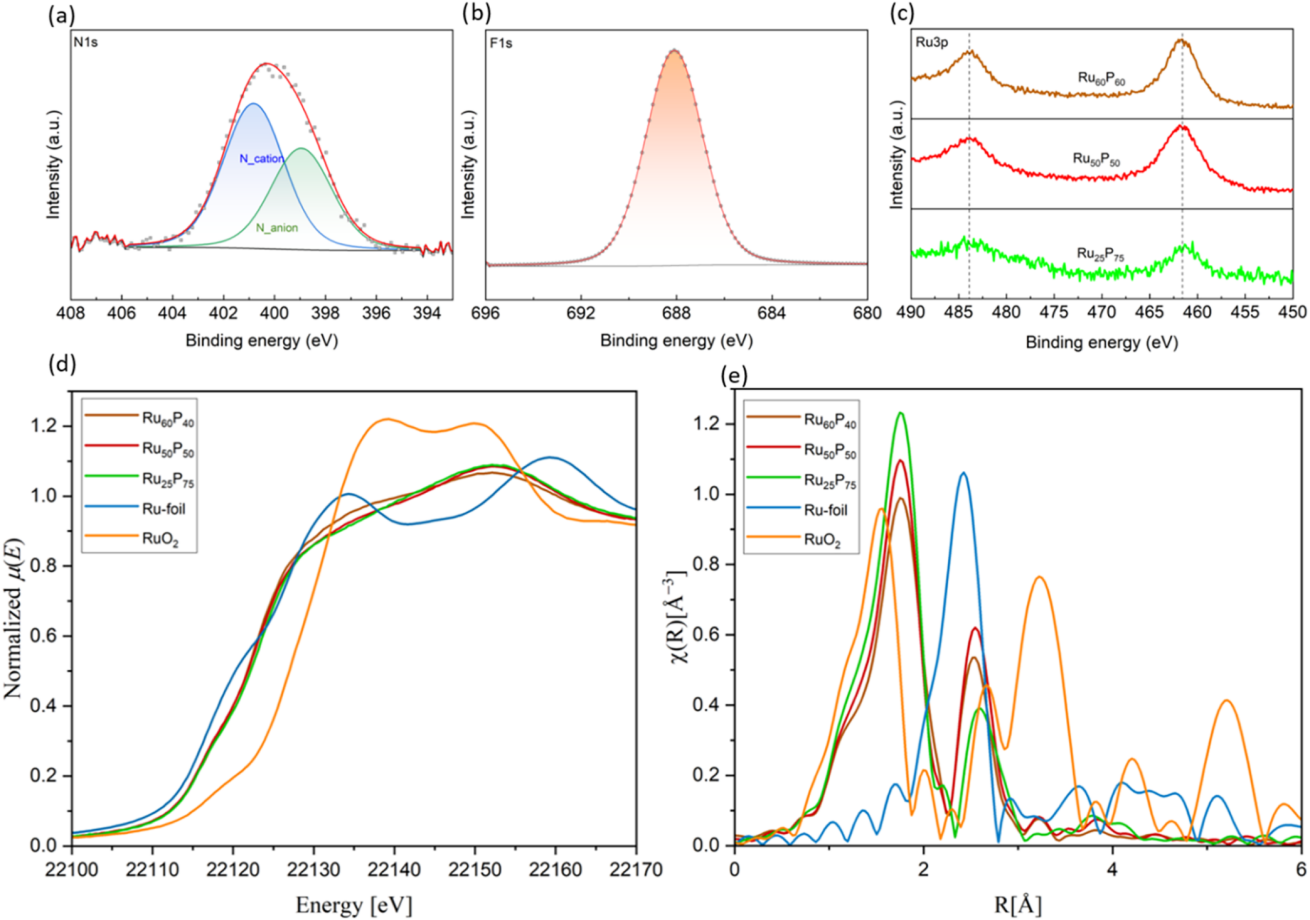
Characterization of Ru_*x*_P_100–*x*_@SILP by X-ray spectroscopies. High-resolution XPS spectra of (a) N 1s; (b) F 1s; and (c) Ru 3p. Ru K-edge XAS of Ru-foil, RuO_2_, and Ru_*x*_P_100–*x*_@SILP (*X* = 25, 50, 60) with (d) normalized XANES spectra and (e) *k* ^2^-weighted *R*-space Fourier transform EXAFS. The *R*-space Fourier transform EXAFS spectra are plotted without phase correction.

In particular, the N 1s component at 400.8 eV is characteristic of the imidazolium cationic part, and the N 1s at 399.0 eV and F 1s at 688.1 eV correspond to NTf_2_^–^ anions.^40,41^ Ru 3p core-level spectra of Ru_*x*_P_100–*x*_@SILP materials revealed Ru 3 p_3/2_ and Ru 3 p_1/2_ binding energies (BEs) at ∼461.5 and 483.7 eV, respectively (Figure 4c), consistent with previously reported values for RuP phases.^34,42^ While a strong Si background signal originating from the SILP prevented detection of the typical P 2p peak (Figure S6a), analysis of the broader P 2s region^43^ in Ru_*x*_P_100–*x*_@SILPs qualitatively confirmed the presence of phosphorus in the NPs (Figure S7).

In addition, XPS analysis of the P 2p region of colloidal Ru_60_P_40_ NPs prepared as a reference showed peaks at 130.2 eV (P 2p_3/2_) and 131.2 eV (P 2p_1/2_) (Figure S6b), similar to what was previously reported for Ru_50_P_50_ NPs water splitting catalysts.^42^ Interestingly, a shift in the P 2s BEs is observed as a function of the NPs composition, suggesting composition-dependent Ru–P interactions that alter the electronic structure (Figure S7). To investigate this effect as well as the electronic and geometric structures of the Ru species within supported Ru_*x*_P_100–*x*_ NPs in further detail, X-ray Absorption Spectroscopy (XAS) analysis was conducted.

The Ru K-edge X-ray absorption near-edge structure (XANES) spectra of Ru_*x*_P_100–*x*_@SILP catalysts exhibit a modest shift toward higher energies compared to metallic Ru, indicative of partial oxidation due to phosphidation (Figure 4d). This edge shift is essentially identical across Ru_25_P_75_@SILP, Ru_50_P_50_@SILP, and Ru_60_P_40_@SILP, showing that the Ru electronic structure is similar irrespective of the Ru:P ratio. These features reflect the covalent character of Ru–P bonding, where phosphorus allows Ru to retain a relatively high electron density and a concomitant partial metallic character. Extended X-ray Absorption Fine Structure (EXAFS) analysis shows a distinct first-shell Ru–P coordination at approximately 2.30–2.33 Å for all compositions (Figure 4e), consistent with established Ru–P distances in crystalline ruthenium phosphides (Table S2). The Ru–Ru bond lengths exhibit a slight systematic variation (Table S3); the P-rich Ru_25_P_75_@SILP catalyst has a slightly longer Ru–Ru distance (∼2.83 Å) compared to those of the more Ru-rich Ru_50_P_50_@SILP and Ru_60_P_40_@SILP (∼2.80 Å). More significantly, compositional effects on coordination numbers (C.N.) are observed (Table S3). Ru_25_P_75_@SILP, with the highest phosphorus content, exhibits the highest Ru–P C.N. (5.4 ± 0.4) and lowest Ru–Ru C.N. (2.6 ± 0.8). In contrast, Ru_50_P_50_@SILP and Ru_60_P_40_@SILP have reduced Ru–P C.N. (∼4.5) and increased Ru–Ru C.N. (∼4), indicating that Ru atoms become more isolated from each other as the phosphorus content increases. However, even in the phosphorus-rich composition, the Ru–P C.N. is below the maximum possible value (6), suggesting incomplete Ru–P coordination, likely due to structural disorder and surface defects. The reduced Ru–Ru coordination numbers relative to bulk Ru phosphides reflect the lack of long-range order and the high fraction of under-coordinated surface atoms inherent to these nanoparticles, where a significant proportion of Ru atoms reside at disordered surfaces rather than in bulk-like crystalline environments. Structural disorder is also reflected by elevated Debye–Waller factors (σ^2^) from EXAFS fitting (Table S3), ranging between 7 and 12 × 10^–3^ Å^2^, significantly higher than well-ordered crystalline references (∼3 × 10^–3^ Å^2^). These high σ^2^ values may be attributed to substantial static disorder, which could arise from atomic-scale defects, local stoichiometric variations, and/or the presence of numerous surface sites in these nanoscale, nonstoichiometric particles.

In summary, while the average Ru electronic state remains unchanged across the Ru_*x*_P_100–*x*_@SILP series, systematic variations in local coordination environments are evident with changing Ru/P ratio: increasing phosphorus content promotes the isolation of Ru centers through higher Ru/P and lower Ru–Ru coordination. The local structure of these catalysts is best described as a pseudoamorphous Ru–P network, characterized by mixed-phase domains, significant static disorder, and a large fraction of under-coordinated surface atoms.

### Catalytic Performance and Substrate Scope

In order to identify the preferred Ru/P ratio, the catalytic performances of Ru_*x*_P_100–*x*_@SILP materials were investigated using the hydrogenation of quinoline **1** as a benchmark (Figure 5a). Quinoline hydrogenation can proceed through two potentially parallel pathways involving either heteroarene hydrogenation to give the 1,2,3,4-tetrahydroquinoline (**1a**) intermediate, or arene hydrogenation to give 5,6,7,8-tetrahydroquinoline (**1b**). **1a** and **1b** can then be hydrogenated to decahydroquinoline (**1c**). Ru-based NPs catalysts typically hydrogenate **1** to **1c** via the formation of the partially hydrogenated intermediate **1a**.^24^

**Figure 5:**
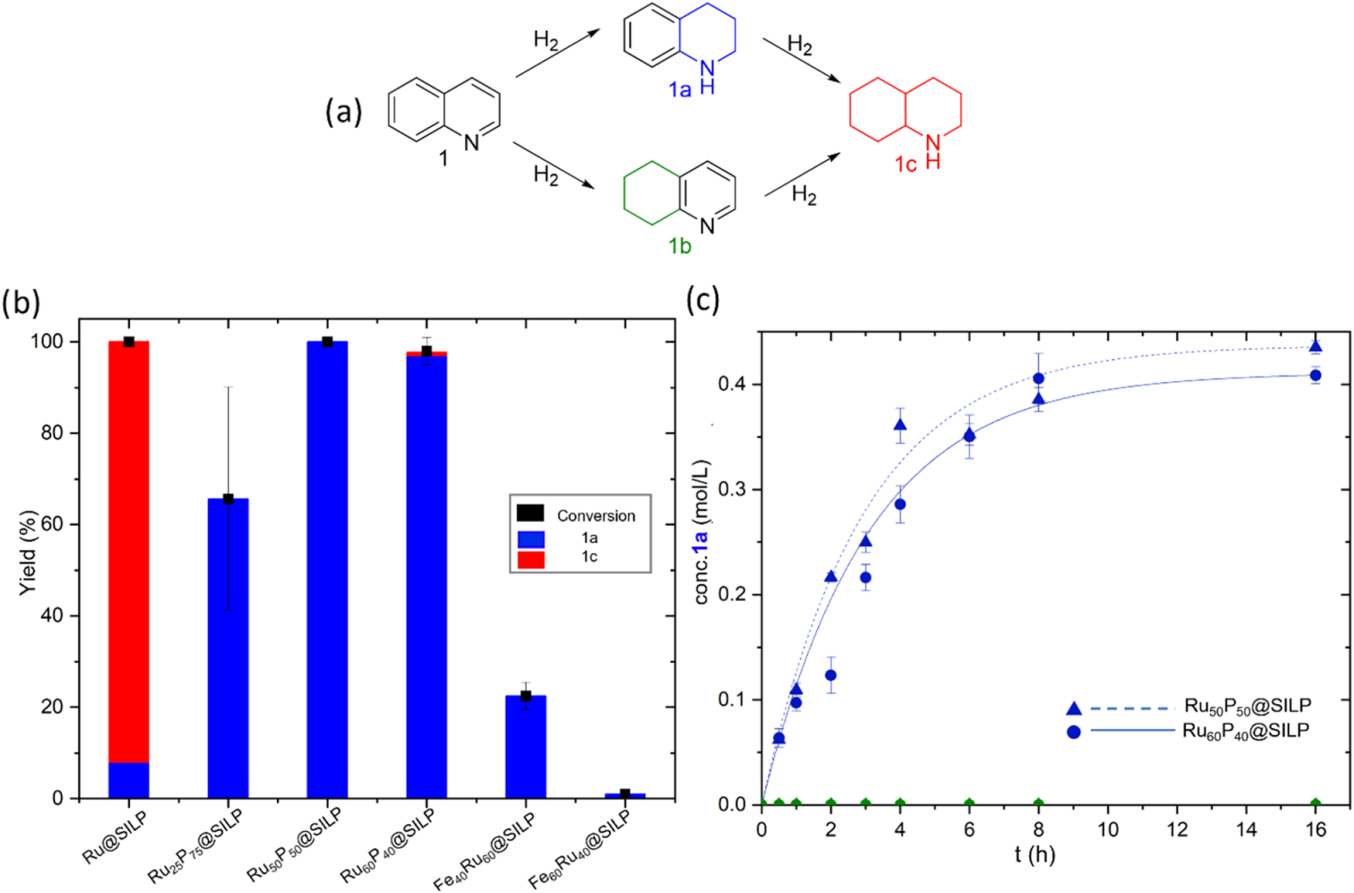
Hydrogenation of quinoline (**1**) using Ru_*x*_P_100–*x*_@SILP and reference catalysts. (a) Reaction network; (b) comparison of Ru_*x*_P_100–*x*_@SILP and reference catalysts under standard conditions: catalyst (10 mg) substrate (53 equiv), heptane (0.5 mL), 90 °C, H_2_ (50 bar), 16 h, 500 rpm; (c) time profiles for the hydrogenation of **1** using Ru_50_P_50_@SILP and Ru_60_P_40_@SILP under adapted conditions: catalyst (10 mg) substrate (53 equiv), heptane (0.5 mL), 50 °C, H_2_ (20 bar), 500 rpm. Each data point is the average of 2–3 experiments and error bars represent standard deviations.

Catalytic reactions were carried out under batch conditions in stainless-steel high-pressure autoclaves equipped with magnetic stir bars. Standard reaction conditions for the comparative study used *n*-heptane as solvent with a substrate-to-total Ru loading of 53 equiv at 90 °C and 50 bar H_2_ for 16 h. Prior to their use in catalysis, Ru_*x*_P_100–*x*_@SILP materials were treated under H_2_ (50 bar) at 175 °C for 3 h to prevent NPs leaching. The pretreatment did not alter the structure of the NPs and the chemisorbed IL layer (see Figures S13–S15 and Table S4 for details). In addition, characterization of Ru_50_P_50_@SILP by solid-state NMR spectroscopy (^31^P Wideband Uniform Rate Smooth Truncation—Carr-Purcell Meiboom-Gill (WURST*-*CPMG) and ^1^H–^13^C and ^1^H–^29^Si cross-polarization (CP)-MAS, Figure S5)^44^ confirmed the unchanged structure of both the ruthenium phosphide NPs and the molecular structure of the chemisorbed ionic liquid layer. Interestingly, the increased presence of T_2_ and T_3_ silicon environments (with T*_n_* representing the number of C^IL^–Si-O^surface^ bonds) as observed in the ^29^Si CP-MAS spectra and the corresponding reduced intensity of Si­(OCH_2_CH_3_) resonances in the ^13^C CP-MAS spectra in the treated catalyst suggest that the treatment promotes stronger attachment of the ionic liquid molecules at the SiO_2_ surface.

The catalytic performance of the three Ru_*x*_P_100–*x*_@SILP in comparison to that of reference Ru_100_@SILP^39^ and bimetallic Fe_*x*_Ru_100–*x*_@SILP^45^ catalysts is shown in Figure 5b. Ru_100_@SILP resulted in the formation of the fully hydrogenated product **1c** (92% yield), consistent with the expected reactivity of Ru NPs under such conditions.^24^ In contrast, ruthenium phosphide NPs-based catalysts were found to be highly selective toward the partially saturated product **1a**. Ru_50_P_50_@SILP delivered the best performance (>99% yield of **1a**), while Ru_25_P_75_@SILP was less active (66% yield of **1a**) and Ru_60_P_40_@SILP was slightly less selective (97% **1a**, 1% **1c**). The RuP catalysts were orders of magnitude more active than previously reported bimetallic Fe_40_Ru_60_@SILP and Fe_60_Ru_40_@SILP catalysts,^46^ exhibiting similarly high selectivity. The observed change in selectivity as compared to Ru@SILP and the activity enhancement as compared to Fe_*x*_Ru_100–*x*_@SILP reveal that the association of phosphorus and ruthenium in phosphide-type nanoparticles results in strong synergistic effects, enabling catalytic performances that are out of reach for reference monometallic and bimetallic Ru-containing NPs.

The substrate:ruthenium ratio could be increased from 53:1 to 533:1 without affecting selectivity, and a turnover number of 533 was reached within 24 h. This experiment allowed determining a turnover frequency (TOF) of 128 h^–1^ for the Ru_50_P_50_@SILP catalyst under these conditions (Figure S16), which is competitive with previously reported state-of-the-art heterogeneous catalysts used in the same transformation (see Table S6 for a comparison). Based on the slightly higher selectivity and activity (Figures 5c and S17) of Ru_50_P_50_@SILP over Ru_60_P_40_@SILP, the material with a Ru/P ratio of 1:1 was chosen to explore its synthetic potential.

The robustness of the Ru_50_P_50_@SILP catalyst was evaluated first by batch-wise recycling using the hydrogenation of quinoline (**1**) as a model reaction (Figure S18a). The reaction time was set to 2 h to ensure incomplete conversion and monitor potential activity changes, and the catalyst was washed with heptane after each cycle. While the excellent selectivity remained uniformly above 99%, the yield of **1a** gradually decreased from the first cycle (52%) to the fourth cycle (25%). Analysis of the spent catalyst using STEM (Figure S13), SEM-EDX, XRF, ICP-OES, and XAS (Figure S15) revealed no substantial NPs growth, Ru leaching, or change in the Ru/P ratio or in the electronic and geometric structures of the NPs, however (Table S5), indicating that the apparent loss of activity was not resulting from instability of the metal particles. Interestingly, repeating the recycling experiments while washing the catalyst with ethanol between each cycle enabled a stable performance for five cycles (∼55% yield, Figure S18b). The regeneration of catalytic activity through washing with a polar solvent such as ethanol indicates that the deactivation originates from the strong binding of the N-heterocycle to the catalyst surface.

Based on the promising activity, selectivity, and recyclability observed in batch-type autoclaves, the performance of Ru_50_P_50_@SILP was also investigated under continuous flow conditions. Indeed, running catalytic processes under continuous flow conditions is particularly attractive to enhance mass and heat transfer, reach higher space-time yields, test catalyst performance and stability under more practically relevant and realistic conditions, and improve operational safety.^47–51^ In this manner, Ru_50_P_50_@SILP was applied under continuous flow (see SI for details) for the hydrogenation of 6-chloroquinoline (**9**) to product **9a** (Figure 6), an important building block for the synthesis of drug molecules, such as the selective histone deacetylase 6 (HDAC6) inhibitor SW-100 tested in the treatment of neurodegenerative disorders.^51^ In addition to the retention of the aromatic ring, dehalogenation poses an additional selectivity challenge for this transformation. Using an H-Cube reactor, a solution of **9** in heptane (0.04 M) was passed through a cartridge containing the Ru_50_P_50_@SILP catalyst bed (250 mg) at a flow rate of 0.7 mL.min^–1^ (residence time τ = 1.14 min). Under 50 °C and 20 bar of H_2_, the yield of **9a** started declining gradually after 2.5 h on stream, consistent with the trend observed in the batch recycling experiments. Flushing the catalyst bed with heptane under flow conditions (25 °C, 30 min) allowed regenerating the catalyst activity, however, confirming accumulation of substrate or (side-)­products at the catalyst material as possible deactivation mechanism.^52^ Running the reaction at higher temperatures (90 °C) to prevent the inhibition allowed stable conversion of **9** to the desired product **9a** with perfect selectivity for over 7 h on stream, corresponding to a space-time yield (STY) of 0.4 kg/L·h. Our results show that the Ru_50_P_50_@SILP catalyst is robust and can be not only recycled under batch conditions but also easily applied under continuous flow conditions while maintaining excellent performance. Notably, this is one of the first examples of catalysts that can be successfully applied in batch and flow for the selective partial hydrogenation of bicyclic heteroaromatic compounds (Table S6). The practical simplicity of the application of Ru_50_P_50_@SILP in a commercial flow system paves the way toward optimization of conditions for maximum productivity, as well as long-term stability tests in the future.

**Figure 6:**
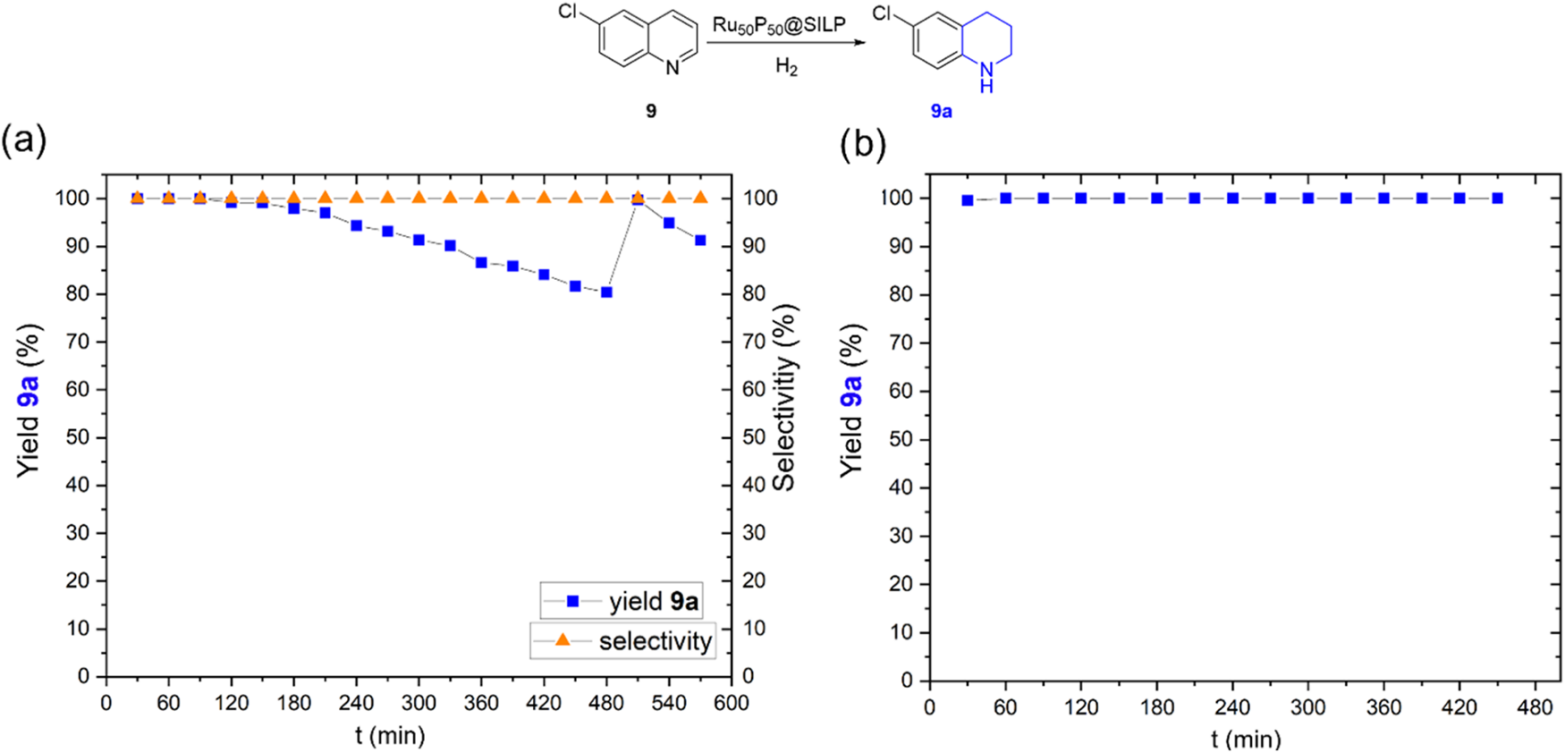
Continuous flow hydrogenation of 6-chloroquinoline (**9**): (a) 50 °C, 20 bar H_2_; (b) 90 °C, 50 bar H_2_. Reaction conditions: Ru_50_P_50_@SILP (250 mg) diluted with 500 mg of glass beads as catalyst, 6-chloroquinoline in heptane (0.04 M) as stock solution. Yields were determined by GC-FID using decane as internal standard. Residence time: 1.14 min. Flow rate: 0.7 mL/min.

Subsequently, the substrate scope of the hydrogenation of bicyclic heteroarenes was explored under batch conditions. In general, quinoline substrates were hydrogenated with excellent selectivity over Ru_50_P_50_@SILP under 90 °C and 50 bar H_2_ (Substrates **1**-**17**). Reaction times between 1 and 5 h were sufficient in most cases to maximize product yield. Hydrogenation was typically found to be slower in the presence of strongly electron-withdrawing groups (e.g., −CF_3_ in substrate **5** and -NO_2_ in substrate **13**), consistent with trends from the literature.^53^ While dehalogenation of N-heterocycles under hydrogenation conditions is a well-documented limitation of most catalytic approaches,^54^ Ru_50_P_50_@SILP demonstrated excellent tolerance to halogen-containing substrates. In addition to 6-chloroquinoline (**9**), also the fluoro (**8**) and bromo (**10**) substituents in the 6-position were fully retained, providing useful building blocks, with only the iodo derivative (**11**) not being tolerated. Substrates with other electron-withdrawing substituents (**5**-**7**) were also effectively converted. While the ester group in substrate **7** proved stable under the present conditions, the acetyl group in **6** was fully deoxygenated at more elevated temperatures (175 °C), providing access to the ethyl-substituted aromatic ring. Even substrates with very bulky isopropyl (**2**) and *tert*-butyl (**3**) groups in 6-position (**2–3**) were quantitatively and highly selectively hydrogenated within 1 h reaction time.

The catalytic hydrogenation worked equally well for other substitution patterns on the quinoline ring. The hydroxyl functional group at the 8-position of substrate **12** was conserved, and product **12a** was formed selectively within 2 h. In the case of 8-nitroquinoline (**13**), the hydrogenation proceeded stepwise within 9 h, with first the rapid reduction of the nitro group to the corresponding amine, followed by the slower hydrogenation of the N-heterocycle to give 1,2,3,4-tetrahydroquinolin-8-amine (**13a**). Methyl substituents in positions 2 and 3 did not affect the performance of the catalyst, and products **14a** and **15a** were obtained in quantitative yield under standard conditions. The annulated Benzo­[*h*]­quinoline (**16**) was converted to [1,2,3,4-tetrahydrobenzo­[*h*]­quinoline] in high yields (91% **16a**) within 5 h. Isoquinolines (**18–19**) were also effectively and selectively hydrogenated to provide partially hydrogenated products **18a** and **19a**. Notably, quinoxaline (**20**) decomposed partially into 1,2-diaminobenzene under standard conditions (Figures S49 and S50), while phenazine (**21**) was selectively hydrogenated to the desired partially saturated product 5,10-dihydrophenazine (**21a**, 97% yield, Figures S51 and S52).

Encouraged by these findings, we extended our study to the hydrogenation of more challenging unprotected indoles, as the potentially resulting indoline derivatives are valuable intermediates in the synthesis of biologically active compounds.^55^ As summarized in Figure 7b, a higher temperature of 130 °C and longer reaction times were required, but good yields could be obtained for products **22a**, **24a**, and **25a**. The conversion of trifluoromethylindol (**23**) bearing the electron-withdrawing −CF_3_ to the desired indoline **23a** proved challenging, consistent with trends from the literature.^17,56,57^ Also indoles bearing Me substituents in positions 1 and 2 (**26** and **27**, respectively) were not fully converted within 16 h. Gratifyingly, selectivity remained uniformly nearly perfect with overhydrogenated products observed below 7%, leaving unreacted substrate largely remaining for further conversion upon extended reaction times (*vide infra*).

**Figure 7:**
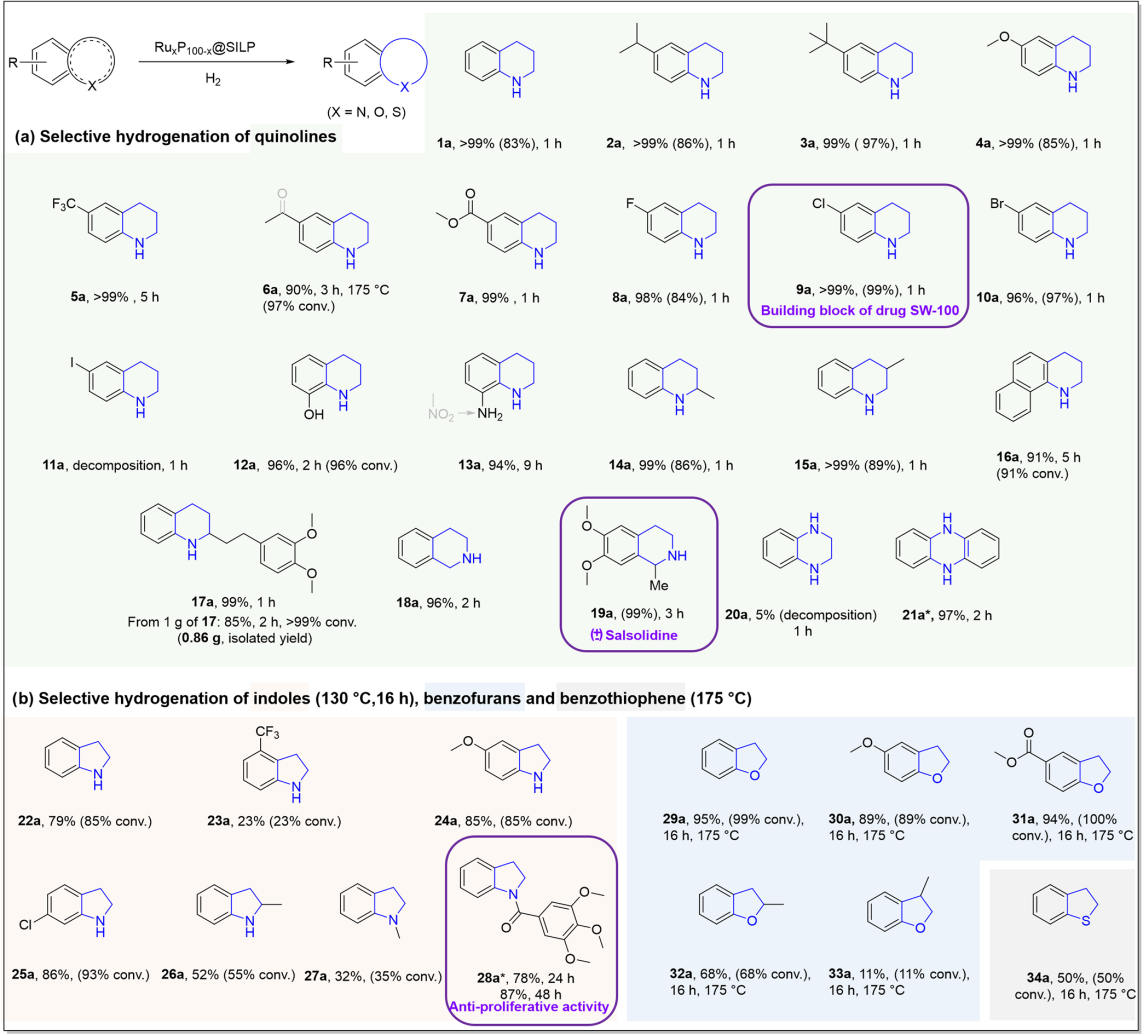
Selective hydrogenation of bicyclic heteroarenes using Ru_50_P_50_@SILP. Catalyst (10 mg, metal content: Ru_50_P_50_@SILP 0.0047 mmol), substrate (for quinolines and indoles: 52–55 equiv, for benzofuranes and benzothiophene = 22–25 equiv), heptane (0.5 mL), 90 °C, H_2_ (50 bar), 1 h (for quinolines) or 16 h (for indoles), 500 rpm. Conversion and product yields were determined by GD-FID using tetradecane or decane as an internal standard. Numbers in the parentheses refer to the isolated yields, conv. = conversion.*:NMR-yield.

Heterocycles with heteroatoms other than nitrogen were also addressed briefly. Even though quite harsh conditions were applied (175 °C, 50 bar, and 16 h), benzofuran derivatives were converted with high selectivity. Excellent yields of the partially hydrogenated products were obtained with either electron-donating or -withdrawing substituents in the aromatic ring (**29–31**). Conversion slowed substantially with methyl substituents in the heterocycle, but yields of up to 68% could still be reached (**32–33**). Although sulfur is generally perceived as a catalyst poison, benzothiophene (**34**) was selectively hydrogenated using Ru_50_P_50_@SILP, yielding product **34a** in 50% yield within the standard reaction time.

### Synthetic Applications

Given the proven robustness and versatility of the Ru_50_P_50_@SILP system, we aimed to validate the application of the catalytic methodology for the synthesis of actual pharmaceutical products by using N-containing heterocycles of various structural patterns.

As an example for quinolines with substituents in 6-position, [6-methoxy-1,2,3,4-tetrahydroquinoline] (**4a**) was obtained after the catalytic hydrogenation and used directly without purification as a building block to give the tubulin polymerization inhibitor^8,25^ **4a′** in excellent isolated yield after purification by flash column chromatography (overall yield of 95%, Scheme 1).

**Scheme 1:**
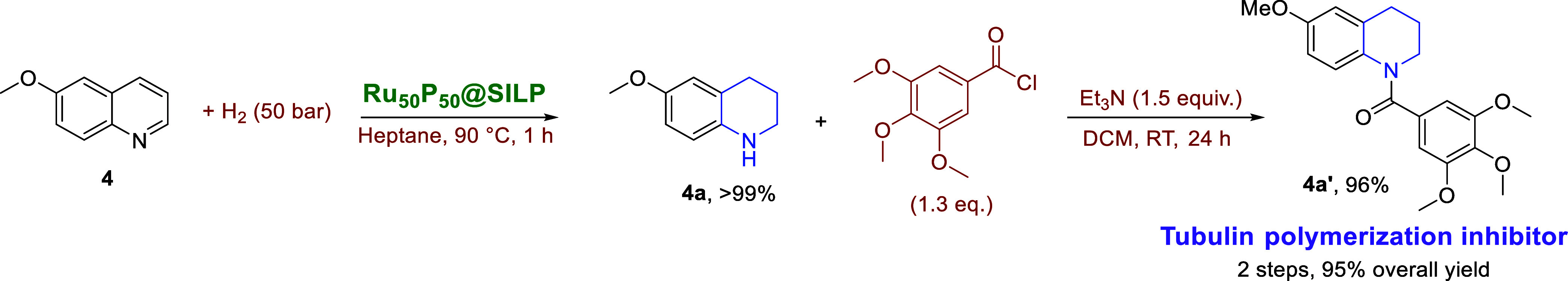
Synthesis of the Tubulin Polymerization Inhibitor **4a′**

The substrate **17** was prepared by coupling of 2-methyl quinoline with 4-(bromomethyl)-1,2-dimethoxybenzene, purified in 91% yield by flash column chromatography, and used for selective hydrogenation to give **17a** (>99%). Methylation at the NH group finalized the synthesis of cytotoxic and antiviral (±)­cuspareine (**17a′**, 88% overall yield, Scheme 2), a Hancock alkaloid used to treat dysentery and fever.^25,27,58^ The selective hydrogenation of **17** to **17a** demonstrated excellent green chemistry metrics,^59^ including an E-factor of 2.01, 100% atom and carbon efficiency, and a reaction mass efficiency of 86%—well within industrial targets (SI Box 1).^59^ These metrics reflect an effective synthetic step generating very little waste, outlining the excellent performance, recyclability, and practicability of the Ru_50_P_50_@SILP catalyst.

**Scheme 2:**
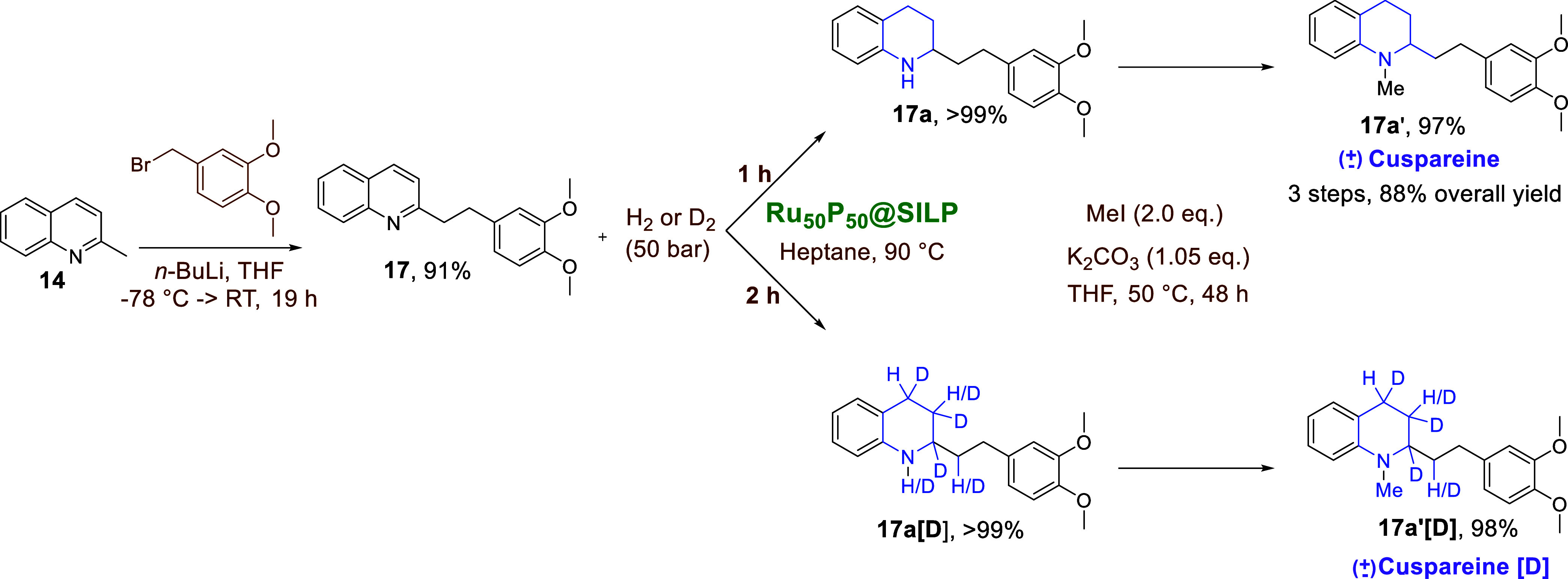
Total Synthesis of (±) Cuspareine (**17a′**) and Its Deuterated Analogue (**17a′[D]**)

In particular, the pathway allowed for the first time the synthesis of deuterium-labeled cuspareine­[D] (**17a′[D]**) via selective catalytic deuteration of **17** to **17a­[D]**. NMR characterizations, including ^2^H and ^13^C­{^1^H,^2^H} experiments, revealed the expected deuterium incorporation via reduction of the heterocyclic C=C bond, along with H/D exchanges occurring not only at the methylene groups of the heterocycle, but also at the bridging methylene group in the vicinity of the nitrogen atom (Scheme 2, and see SI Section S10 for detailed characterization). HR-MS analysis of **17a­[D]** and **17a′[D]** showed the predominance of the [D4] isotopomer in the obtained mixtures (containing [D1] to [D6], see SI Section S10). These results emphasize the utility of the Ru_50_P_50_@SILP catalyst for the selective isotope labeling of drug molecules, providing various potential benefits including the reduction of toxicity and doses, and the improvement of solubility and pharmokinetic activity.^60^

The selective hydrogenation of isoquinolines over Ru_50_P_50_@SILP was validated as an effective approach to (±)-salsolidine (**19a**), an alkaloid exhibiting a range of valuable pharmacological activities (Scheme 3).^61,62^ The substrate 6,7-dimethoxy-1-methyl-3,4-dihydroisoquinoline (**19**) was synthesized *via* a two-step Bischler–Napieralski reaction following a literature protocol.^63^ The hydrogenation of **19** was quantitative under standard conditions within 3 h, giving (±)-salsolidine (**19a**) in 92% overall isolated yield after filtration and solvent removal (Scheme 3).

**Scheme 3:**
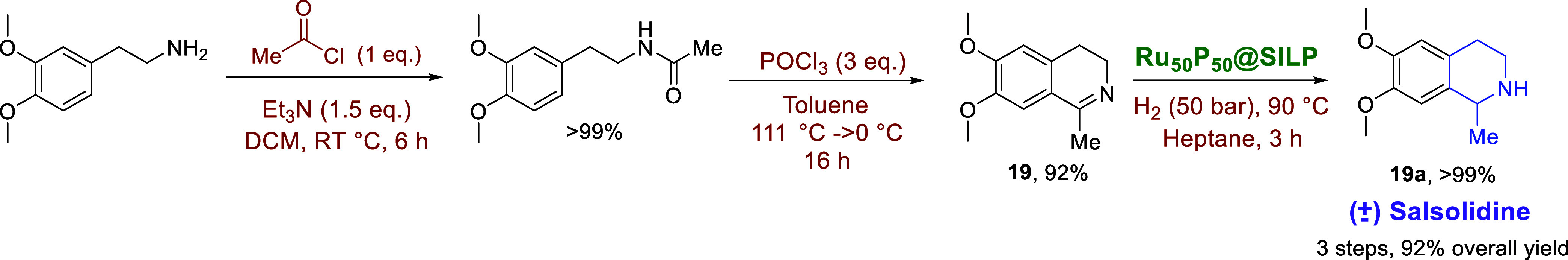
Total Synthesis of (±) Salsolidine (**19a**)

The application to indole-derived pharmaceuticals was demonstrated for the antiproliferative and anticancer drug [indolin-1-yl­(3,4,5-trimethoxyphenyl)­methanone] (**28a**).^64,65^ The substrate [(1H-indol-1-yl)­(3,4,5-trimethoxyphenyl)­methanone] (**28**) was prepared via the coupling reaction of indole with trimethoxybenzoyl chloride and obtained in 88% isolated yield after purification by flash column chromatography. Hydrogenation over Ru_50_P_50_@SILP required a prolonged reaction time of 48 h but gave **28a** in an excellent 77% yield over the two steps (Scheme 4).

**Scheme 4:**
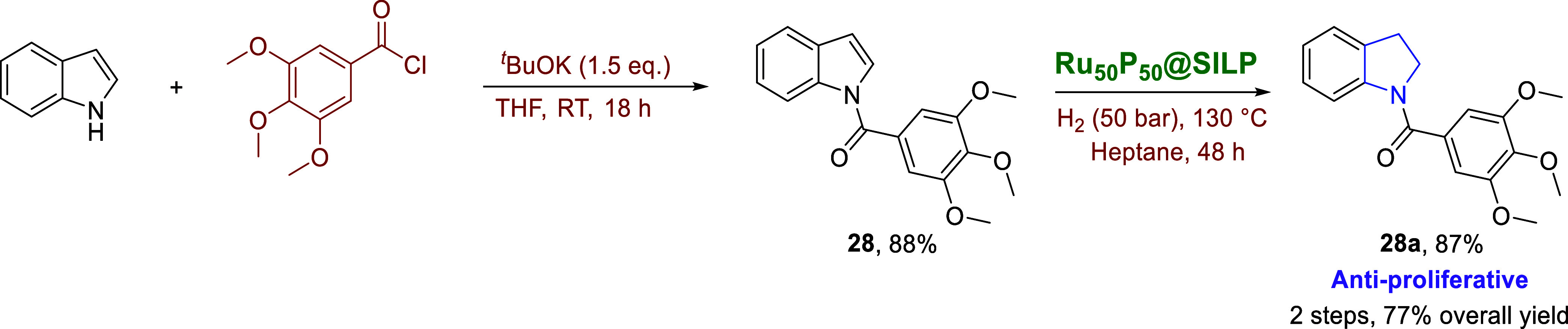
Total Synthesis of the Antiproliferative Agent **28a**

## Conclusion

Ruthenium phosphide nanoparticles immobilized on silica modified with an imidazolium-based supported ionic liquid, Ru_*x*_P_100–*x*_@SILP, provide a new type of catalyst material for selective hydrogenation reactions. In the present study, Ru_50_P_50_@SILP was found to comprise the optimum Ru/P ratio for use as an active and highly selective catalyst for the partial hydrogenation of bicyclic heteroaromatic substrates. The catalyst performed well for a remarkably broad substrate scope, including quinolines, indoles, benzofurans, and benzothiophene, and was applied successfully to a wide range of structurally diverse substrates, and its practical potential was demonstrated in the synthesis of actual pharmaceuticals and drug molecules, including deuterated analogues. It proved structurally robust and suitable for continuous flow operation.

In general terms, the molecular approach to the preparation of supported metal phosphide nanoparticles is simple and versatile, producing materials with precisely defined particle sizes and adjustable metal–phosphorus ratios. The modular preparation technique utilizing suitable metal precursors across a wide range of the periodic table in connection with various support materials provides access to a large variety of possible materials. The present study thus opens a broad range of opportunities beyond the exemplified application to explore a new class of catalyst materials, allowing for fine-tuned nanoparticle composition, functional supports, and nanoparticle-support interactions. It may thus pave the way toward the development of other supported metal phosphide catalysts to tackle challenging transformations.

## Supplementary Material



## Data Availability

The data utilized in this study areavailable for download on Edmond (open data repository of the Max Planck Society) at https://doi.org/10.17617/3.JTPNT3.

## Notes

Experimental procedures; characterization techniques; characterization data for Ru*_x_* P_100–*x*_@SILP; crystal structure parameters for ruthenium phosphides; and ^1^H NMR (400 MHz, CDCl_3_) spectrum of 2-(3, 4-dimethoxyphenethyl)­quinoline (**17**) (PDF)
