# Supplementary material for: Selective Hydrogenation of Heteroarenes Using Supported Ruthenium Phosphide Nanoparticle Catalysts
Source: J Am Chem Soc. 2025 Dec 23;148(1):766–77. doi: 10.1021/jacs.5c16144 (PMC12814184; doi:10.1021/jacs.5c16144)
Supplement: Supplementary file 1 [file ja5c16144_si_001.pdf]

SUPPORTING INFORMATION FOR:

Selective Hydrogenation of Heteroarenes Using Supported Ruthenium Phosphide Nanoparticle Catalysts

Hooman Ghazi Zahedi,[ab] Jannis Hertel,[a] Bhaskar Paul,[a] Liqun Kang,[a] Jacob Johny,[a] Yufei Wu,[a] Thomas Wiegand,[ab] Serena DeBeer,[a] Walter Leitner,[ab] Alexis Bordet* [a]*

[a]Max Planck Institute for Chemical Energy Conversion, Stifstrasse 34-36, 45470 Mülheim an der Ruhr (Germany)

[b]Institute of Technical and Macromolecular Chemistry, RWTH Aachen University, Worringerweg 2, 52074 Aachen (Germany)

E-mail: walter.leitner@cec.mpg.de / alexis.bordet@cec.mpg.de

KEYWORDS. Ruthenium phosphides, nanoparticles, selective hydrogenation, heteroarenes

Contents

1. Safety Warning S3
2. General S3
3. Analytics S3
   3.1 Electron Microscopy S3
   3.2 Solution-State NMR Spectroscopy S4
   3.3 Solid-State NMR Spectroscopy S4
   3.4 ICP-OES S6
   3.5 XRF S6
   3.6 XPS S6
   3.7 X-Ray Absorption Spectroscopy S6
   3.8 Mass Spectrometry S7
   3.9 GC Analysis S8
4. Synthesis S9
   4.1 Synthesis of Supported Metal Phosphide Nanoparticles S9
   4.2 Synthesis of Substrates **17**, **19**, and **28** S9
   4.3 Synthesis of (6-methoxy-dihydroquinolin-1-yl)(3,4,5-trimethoxyphenyl)-methanone (**4a'**) .. S11
   4.4 Synthesis of Cuspareine (**17a'**) S11
5. Catalytic Experiments S12
   5.1 Turnover Number (TON) and Frequency (TOF) S12
   5.2 Product Isolation S12
   5.3 Catalyst Recycling S13
   5.4 Continuous Flow Experiments S13
   5.5 Time Profiles S14
6. Supplementary Tables and Figures S14
7. Box 1. Green Chemistry Metrics S29
8. NMR Data for Isolated Products. S31
9. NMR Spectra. S34
10. Characterization of 17a, 17a[D], 17a' and 17a'[D] S52
11. References S62

1. Safety Warning

Tristrimethylsilylphosphine ($P(SiMe_3)_3$) is a pyrophoric substance and may readily produce phosphine gas (PH_3) upon reaction with water or a protic medium. Therefore, it was stored under inert conditions in the freezer of an argon-filled glovebox and handled with caution, within the glovebox or outside using standard Schlenk techniques to prevent exposure to air. All the equipment or tools contaminated with $P(SiMe_3)_3$ were washed inside the fume hood using isopropanol and water.

2. General

Unless otherwise noticed, the preparation and washing process of nanoparticles, ionic liquids (ILs), supported ionic liquid phases (SILPs), and nanoparticles immobilized on SILPs (NPs@SILPs) were performed under an inert argon atmosphere using standard Schlenk techniques or within a glovebox. All synthesized materials were stored in the glovebox. Commercially available substrates were used as received, without any further purification. The solvents were degassed and dried over molecular sieves and subsequently stored in the glovebox.

3. Analytics

3.1 Electron Microscopy

Scanning electron microscopy with energy dispersive X-ray spectroscopy (SEM/EDX) was performed on a Hitachi TM-3030 microscope operating at 30kV at the Max-Planck-Institut für Kohlenforschung (45470, Germany). Samples were prepared by depositing the material on a carbon tap for the analysis. Transmission electron microscopy (TEM) and scanning transmission electron microscopy (STEM) in high-angle annular dark-field mode (HAADF) coupled with energy dispersive X-ray spectroscopy (EDX) were performed on a FEI Talos F200X operating a 200 kV equipped with a SuperX (4 Bruker SDD EDX detectors).

3.2 Solution-State NMR Spectroscopy

The solution-state NMR spectra were recorded using a Bruker Ascend 400 and 500 MHz spectrometers at ambient temperature. Isotropic chemical-shift values (δ) are reported in ppm and were referenced to the resonances of the corresponding deuterated solvent for ^{1}H NMR and ^{13}C-NMR. J-coupling constants (J) are reported in Hertz (Hz).

3.3 Solid-State NMR Spectroscopy

Solid-state NMR spectra were recorded on a Bruker wide-bore 500 MHz magnet (11.7 T magnetic-field strength, ^{13}C Larmor frequency of 125.7 MHz, ^{29}Si Larmor frequency of 99.3 MHz, ^{31}P Larmor frequency of 202.4 MHz) equipped with a 3.2 mm Bruker triple-resonance probe. All NMR spectra were processed with the software TopSpin version 4.1.4 (Bruker Biospin). The envelope shape of the WURST-CPMG spikelet pattern was extracted with UWNMRSpectralShape software[1] and fitted with the software DMFit (release #20230120).[2] All experimental parameters are summarized in the tables below.

The ^{1}H-^{13}C and ^{1}H-^{29}Si solid-state CP-MAS NMR spectra were recorded at a MAS frequency of 17.0 kHz. The temperature of the cooling gas was maintained at 280 K to compensate for frictional heating. The ^{1}H-^{13}C CP parameters were optimized on a 1,2-^{13}C- and ^{15}N-labelled glycine ethyl ester standard sample. The ^{1}H-^{29}Si CP parameters were optimized on a ^{29}Si-labelled octakis(trimethylsiloxy)silsesquioxane sample. After the ^{1}H excitation pulse, adiabatic polarization transfer under the Hartmann-Hahn condition was achieved by a ramped-amplitude cross-polarization step,[20-21] with $\nu_{RF}(^{1}H)$ being swept from 48 to 72 kHz using a tangent ramp, while $\nu_{RF}(^{13}C)$ or $\nu_{RF}(^{29}Si)$ were kept at 43 kHz. 90 kHz SPINAL-64 ^{1}H decoupling was applied during data acquisition.[3] Isotropic chemical-shift values were referenced indirectly to tetramethylsilane using adamantane as a secondary standard for ^{1}H and ^{13}C (**C**H_2 $\delta = 38.48$ ppm)[4-5] and octakis(trimethylsiloxy)silsesquioxane for ^{29}Si (O**Si**$(CH_3)_3$ $\delta = 11.5$ ppm).[6]

The ^{31}P WURST-QCPMG spectra were recorded under static conditions. The temperature of the cooling gas was maintained at 290 K. The ^{31}P pulse length was calibrated on an $NH_4H_2PO_4$ standard sample. A WURST-80 pulse[7] with an rf-field of 27 kHz, a sweep width of 1 MHz, a length of 80 µs, and 1000 increments was used. ^{31}P chemical-shift values were referenced indirectly to 85 % H_3PO_4 solution using $NH_4H_2PO_4$ as a secondary standard ($\delta = 0.8$ ppm).[8]

Overview of the experimental parameters used for ^{1}H-^{13}C and ^{1}H-^{29}Si CP-MAS NMR measurements.

	RuP@SILP ^{1}H-^{13}C	RuP@SILP ^{1}H-^{29}Si	RuP@SILP-treated [illegible]	RuP@SILP-treated [illegible]
ν_r / kHz	17.0	17.0	17.0	17.0
B_0 / T	11.7	11.7	11.7	11.7
$\nu_1(^{1}H)$ excitation / kHz	100	100	100	100
CP contact power $\nu_1(^{1}H)$ / kHz	48 to 72	48 to 72	48 to 72	48 to 72
CP contact power $\nu_1(^{13}C$ or $^{29}Si)$ / kHz	43	43	43	43
CP contact time / ms	1.5	5.0	1.5	5.0
^{1}H carrier / ppm	2.3	2.3	2.3	2.3
^{13}C or ^{29}Si carrier / ppm	43.7	-79.8	43.7	-79.8
Sweep width / ppm	795	1198	795	1198
Acquisition time / ms	10.2	12.9	10.2	12.9
Recycle delay / s	2.0	1.2	2.0	1.2
Number of scans	32000	48000	32000	16000
Measurement time/ h	17.8	16.1	17.8	5.4

Overview of the experimental parameters used for ^{31}P WURST-QCPMG measurements.

	RuP@SILP	RuP@SILP treated
ν_r / kHz	0 (static)	0 (static)
B_0 / T	11.7	11.7
ν_1 / kHz	27	27
Shape	WURST-80	WURST-80
Carrier / ppm	410	410
Sweep width / ppm	4938	4938
Echo delay / µs	10	10
Number of echoes	17	17
Acquisition time / ms	3.1	3.1
Recycle delay / s	2.0	2.0
Number of scans	32000	32000
Measurement time/ h	17.8	17.8

3.4 ICP-OES

Inductively Coupled Plasma Optical Emission Spectroscopy (ICP-OES) was performed by Mikroanalytisches Labor Kolbe via Spectro Arcos ICP-OES (Spectro). For sample preparation a CEM-Mars 6 microwave digestion system was utilized.

3.5 XRF

Xepos C instrument from Spectro was used for X-ray fluorescence (XRF) spectroscopy. A 12 µm polypropylene film served as the sample support. Measurements were recorded over a duration of 600 seconds under a helium/air atmosphere in a range between 3 keV and 19 keV.

3.6 XPS

X-ray photoelectron spectroscopy (XPS) measurements were conducted on Ru_xP_{100-x}@SILPs and isolated $Ru_{60}P_{40}$ NPs using a near ambient pressure (NAP) XPS (Specs GmbH) employing a monochromated Al-Kα source having energy 1486.6 eV. All XPS samples were prepared inside a glove box and transferred into the XPS chamber under controlled atmosphere. The high-resolution scans were recorded using a pass energy of 20 eV and a resolution of 0.05 eV while a pass energy of 100 eV was used for the survey scans. The recorded spectra were corrected using C1s binding energy at 285.0 eV and the data analysis was performed using the CasaXPS where a Shirley-type background was applied for deconvolution of high-resolution spectra.

3.7 X-Ray Absorption Spectroscopy

The Ru K-edge XAS spectra for the fresh catalysts ($Ru_{25}P_{75}$@SILP, $Ru_{50}P_{50}$@SILP, and $Ru_{60}P_{40}$@SILP) were collected at the P65 beamline of PETRA III (Germany).[9] At this beamline, the X-ray beam was generated using the 5th-order harmonic radiation from an 11-period undulator. A water-cooled, fixed-exit Si(311) double crystal monochromator (DCM) was employed to monochromatize the beam. The DCM was operated in QEXAFS mode, and the undulator offset was calibrated to the DCM to maximise photon flux. The beam was then focused by Pt-coated mirrors and collimated at the sample position to a spot size of approximately 1.0 mm × 0.5 mm (H × V). The Ru K-edge XAS spectra for the post-synthetically treated catalyst ($Ru_{50}P_{50}$@SILP-treated) and the spent catalyst ($Ru_{50}P_{50}$@SILP-PHT-Spent) were recorded at the SAMBA beamline[10] of the SOLEIL synchrotron. These two samples were prepared from a separate batch, and to ensure consistency between the two XAS setups, the untreated catalyst ($Ru_{50}P_{50}$@SILP)

from the same batch was also measured. At SAMBA beamline, X-rays were generated by a bending magnet, and a Si(220) monochromator operated in QEXAFS mode was used to produce the monochromatic beam.[11] The beam was focused by Pd-coated mirrors and collimated at the sample position to a spot size of approximately 1.5 mm × 1.0 mm (H × V). To prevent exposure to air or moisture, each catalyst powder was sealed in custom-designed anaerobic sample cells, using Kapton films as entrance and exit windows for the X-rays. All samples were prepared in a glovebox and transferred anaerobically to the beamline. Measurements were conducted at room temperature. For each sample, the Ru K-edge XAS spectra were collected 3–5 times in transmission mode and subsequently merged to enhance the signal-to-noise ratio. A Ru foil was measured concurrently with each sample to calibrate the energy scale, with the first inflection point in the first derivative of the XANES spectrum of Ru foil set to 22,117 eV. Additionally, commercial RuO_2 powder was measured in transmission mode as a reference. The full XAFS energy ranges for spectra collected at P65 and SAMBA were 21,942–22,942 eV (k_{max} = 14.6) and 21,942–23,312 eV (k_{max}= 17.7), respectively, with energy step sizes of 0.4 eV and 0.5 eV.

The Ru K-edge XAS data were analysed using the Demeter software suite (Athena and Artemis, version 0.9.26).[12] Pre-edge background subtraction and post-edge normalization were performed in Athena. A linear regression background was determined in the 21,943–22,043 eV range, and a quadratic polynomial was fitted for post-edge normalization in the 22,223–22,923 eV region. The spectra were splined from $k = 0$ Å^{-1} to $k = 14.5$ Å^{-1}, with an rbkg of 1.0 and a k-weight of 2. EXAFS fitting (R-range: 1–3.3 Å; k-range: 2.7–13 Å^{-1}) was carried out in Artemis using scattering paths generated from FEFF6. The amplitude reduction factor (S_0^2) was determined to be 0.69 by fitting the k^2-weighted R-space EXAFS of Ru metal powder, based on crystallographic parameters from the Crystallography Open Database (COD ID: 9008513). This value was fixed for the EXAFS fitting of all other Ru samples.

3.8 Mass Spectrometry

High resolution mass spectrometry (HR-MS) was performed at Max-Planck Institute für Kohlenforschung using an Exactive GC Orbitrap spectrometer from Thermo Scientific. MS 8 Rxi-5MS was used as the column with a length of 30 m. GC-EI was chosen as the ionization method, starting at 35°C with a heating rate of 15 and a final temperature of 320 °C.

GC-MS measurements were performed on a GC2010 Plus equipped with a MS QP-2020 from Shimadzu. Depending on the substrate, either an Rtx-1 column (Restek; 30 m length, 0.25 µm film thickness, 0.25 mm inner diameter) or a CP Wax 52 CB column (Agilent; 60 m length, 0.25 µm film thickness, 0.25 mm inner diameter) was used.

3.9 GC Analysis

The Quantification of the products was achieved using gas chromatography (GC) via a Shimadzu Nexis 2030 system equipped with a flame ionization detector (FID). The analysis methods are summarized in the table below. GC measurements for the quinoline and indole substrates were performed following GC Method No. 1, while the benzofuran and benzothiophene substrates were analyzed following Method No. 2.

Methode Nr.	1	2
Stationary phase (Column)	Rtx-1, Restek 30m, Ø 0.25 mm, 0.5 µm Film	CP Wax, 52 CB Agilent 60 m, Ø 0.25 mm, 0.25 µm Film
Mobile Phase [mL/min] (Carrier Gas)	Helium 43.5 ml/min	Helium 72.3 ml/min
Flow Control Mode Linear Velocity [cm/sec]	35 cm/s	35 cm/s
Injection Volume [µL]	1 µL	0.3 µL
Split Ratio	25	30
Temperature Program [°C]	Start 50°C with 10K/min to 200°C, holding for 5 min	Start 70°C with 10K/min to 200°C, holding for 5 min
Detector Temperature [°C]	250°C	250°C

4. Synthesis

4.1 Synthesis of Supported Metal Phosphide Nanoparticles

a) Synthesis of $Ru_{25}P_{75}$@SILP

In a Schenk tube, $P(SiMe_3)_3$ (66 mg, 0.25 mmol, 3.0 eq. 95%-purity) was added to a mixture of [$RuCl_2$(cymene)] (27 mg, 0.08 mmol, 1.0 eq., 95%-purity) and SILP (500 mg) in mesitylene (3.6 mL), and stirred for 16 h at 60 °C. Subsequently the resulting black powder was washed with (5 x 5 mL) toluene and dried under reduced pressure for 7 h.

b) Synthesis of $Ru_{50}P_{50}$@SILP

In a Schlenk tube, $P(SiMe_3)_3$ (66 mg, 0.25 mmol, 1.0 eq. 95 %-purity) was added to a suspension of [$RuCl_2$(cymene)] (80.5 mg, 0.25 mmol, 1.0 eq., 95 %-purity) and SILP (500 mg) in mesitylene (3.6 mL). After stirring for 16 h at 60 °C the resulting black powder was washed with (5 x 5 mL) toluene and dried under reduced pressure for 7 h.

c) Synthesis of $Ru_{60}P_{40}$@SILP

Following the same strategy using a Schlenk tube, $P(SiMe_3)_3$ (44 mg, 0.17 mmol, 0.7 eq. 95%-purity) was added to a mixture of [$RuCl_2$(cymene)] (80.5 mg, 0.25 mmol, 1.0 eq., 95% purity) and SILP (500 mg) in mesitylene (3.6 mL). After stirring for 16 h at 60 °C the resulted black powder was washed with (11 x 5 mL) toluene and subsequently dried under vacuum for 7 h.

4.2 Synthesis of Substrates **17**, **19**, and **28**

a) Synthesis of 2-(3,4-dimethoxyphenethyl)quinoline (**17**). This molecule was synthesized according to a literature procedure.[13] To a Schlenk round-bottom flask (250 mL), 2-methylquinoline (15 mmol) and dry tetrahydrofuran (THF, 50 mL) were taken under nitrogen. Then, the flask was cooled to -78 °C before adding n-BuLi (18 mmol, 2.5 M in n-Hexane) dropwise over 15-20 minutes. The reaction mixture was slowly warmed to room temperature and stirred for 2 h. The solution was again cooled to -78 °C and then a THF (10 mL) solution of 4-(bromomethyl)-1,2-dimethoxybenzene (16.5 mmol) was added dropwise over 30 minutes. The reaction mixture was stirred at -78 °C for 2 h, and then slowly warmed to room temperature, and stirred for another 15 h. The solution was slowly quenched by dropwise addition of ice-cold water under nitrogen. The reaction mixture was extracted with ethyl acetate (3 x 30 mL) and stored over Na_2SO_4. Solvent was evaporated and the crude reaction mixture was purified by silica-gel column chromatography

by using ethyl acetate/ pentane (1:10 to 1:3) as eluent which afforded the product as a light-yellow oil (Yield: 13.65 mmol, 4.0 g, 91%). **^{1}H NMR** (400 MHz, $CDCl_3$) δ (ppm): 8.08 (d, J = 8.5 Hz, 1H), 8.03 (d, J = 8.5 Hz, 1H), 7.76 (dd, J = 8.3, 1.5 Hz, 1H), 7.69 (ddd, J = 8.4, 6.9, 1.5 Hz, 1H), 7.48 (ddd, J = 8.3, 6.9, 1.5 Hz, 1H), 7.22 (d, J = 8.3 Hz, 1H), 6.77 (s, 2H), 6.75 (s, 1H), 3.84 (s, 3H), 3.79 (s, 3H), 3.29-3.25 (m, 2H), 3.12-3.08 (m, 2H). **^{13}C{^{1}H}NMR** (101 MHz, $CDCl_3$) δ 161.9, 148.8, 147.9, 147.3, 136.3, 134.1, 129.5, 128.8, 127.6, 126.8, 125.9, 121.7, 120.4, 111.9, 111.2, 55.9, 55.8, 41.3, 35.7.

b) Synthesis of 6,7-dimethoxy-1-methyl-3,4-dihydroisoquinoline (**19**). This molecule was synthesized according to a literature procedure.[14] **Step 1:** To a Schlenk round-bottom flask (250 mL), acetyl chloride (20 mmol), arylethylamine (20 mmol), and dry DCM (60 mL) were taken. The solution was cooled to 0 °C and then triethylamine (30 mmol) was slowly added over 30 minutes. The reaction mixture was stirred at room temperature for 6 h and then quenched with water (50 mL). The reaction mixture was extracted with DCM (3 x 30 mL), and dried over Na_2SO_4. The solvent was removed and the orange-yellow residue was used in the next step without further purification.

Step 2: A solution of N-(3,4-Dimethoxyphenethyl)acetamide (15 mmol) in toluene (40 mL) was warmed to 60 °C under air and then phosphoryl chloride (45 mmol) was added. The solution was refluxed for 3 h and was then cooled to 0 °C for 3 h. The solution was decanted and to the remaining solid water was added slowly. The reaction mixture was basified with aqueous sodium hydroxide solution and then extracted with dichloromethane (4 × 50 mL). The solution was dried over Na_2SO_4, and the crude product was purified by flash column chromatography using pentane/ethyl acetate (2:1) as eluent which afforded the pure product as an orange yellow crystalline solid (Yield: 18.4 mmol, 3.77 g, 92%). **^{1}H NMR** (400 MHz, $CDCl_3$) δ (ppm): 6.96 (s, 1H), 6.66 (s, 1H), 3.88 (s+s, 6H), 3.60 (tq, J = 7.6, 1.5 Hz, 2H), 2.61 (d, J = 7.6 Hz, 2H), 2.34 (t, J = 1.5 Hz, 3H). **^{13}C{^{1}H}** NMR (101 MHz, $CDCl_3$) δ 163.8, 150.9, 147.5, 131.2, 122.5, 110.3, 109.1, 56.3, 56.0, 47.0, 25.8, 23.5.

c) Synthesis of (1H-indol-1-yl)(3,4,5-trimethoxyphenyl)methanone (28). This molecule was synthesized according to the literature procedure.[15] To a Schlenk round-bottom flask (250 mL), indole (15 mmol), potassium tert-butoxide (22.5 mmol), and dry THF (100 mL) were taken under

nitrogen. The reaction mixture was stirred for 1 h at room temperature and then solid 3, 4,5-trimethoxybenzoyl chloride (22.5 mmol) was added. The reaction mixture was stirred for 18 h and then quenched with water (70 mL). The reaction mixture was extracted with ethyl acetate (3 x 40 mL) and then solution was stored over Na_2SO_4. Solvent was removed under reduced pressure and the crude residue was purified by silica-gel column chromatography by using ethyl acetate/ pentane (1:5 to 1:2) as eluent which afforded the product as a pale-yellow crystalline solid (Yield: 13.2 mmol, 4.1g, 88%). **^{1}H NMR** (400 MHz, $CDCl_3$) δ (ppm): 8.36 (d, J = 9.3 Hz, 1H), 7.60 (d, J = 7.6 Hz, 1H), 7.29-7.35 (m, 2H), 7.30 (m, 1H), 6.98 (s, 2H), 6.62 (d, J = 3.0 Hz, 1H), 3.94 (s, 3H), 3.88 (s, 6H). **^{13}C{^{1}H} NMR** (101 MHz, $CDCl_3$) δ 168.2, 153.2, 141.2, 136.0, 130.7, 129.5, 127.6, 124.9, 123.9, 120.9, 116.3, 108.5, 106.8, 61.0, 56.4.

4.3 Synthesis of (6-methoxy-3,4-dihydroquinolin-1(2H)-yl) (3,4,5-trimethoxyphenyl)-methanone (**4a'**)

This molecule was synthesized according to a literature procedure.[16] In a Schlenk round-bottom flask (25 mL), 6-methoxy-1, 2, 3, 4-tetrahydroquinoline (1.0 mmol), and dry dichloromethane (5.0 mL) were taken under nitrogen. Then, triethylamine (1.5 mmol), and 3,4,5-trimethoxybenzoyl chloride (1.3 mmol) were added in stepwise way. The reaction mixture was stirred for 15 h at room temperature and then the crude residue was purified by flash chromatography with silica using pentane/ethyl acetate as eluent (80: 20) which afforded the product **4a'** as a colorless liquid (Yield: 0.96 mmol, 343.0 mg, 96%).

^{1}H NMR (400 MHz, $CDCl_3$) δ (ppm): 6.68 (s+s, 2H), 6.57 (s, 2H), 6.48 (d, J = 6.5 Hz, 1H), 3.86 (t, J = 6.5 Hz, 2H), 3.82 (s, 3H), 3.73 (s, 3H), 3.68 (s, 6H), 2.79 (t, J = 6.5 Hz, 2H), 2.02 (p, J = 6.5 Hz, 2H). **^{13}C{^{1}H} NMR** (101 MHz, $CDCl_3$) δ 169.5, 156.7, 152.8, 139.6, 133.1, 132.7, 131.3, 126.4, 113.2, 111.5, 106.3, 61.0, 56.1, 55.5, 44.7, 27.3, 24.3.

4.4 Synthesis of Cuspareine (**17a'**)

The methylation experiment was performed according to a literature procedure.[17] In a nitrogen filled Schlenk tube (100 mL), 2-(3,4-dimethoxyphenethyl)-1,2,3,4-tetrahydroquinoline (**17a**, 856.5 mg, 2.9 mmol), methyl iodide (2.0 equiv.), potassium carbonate (1.05 equiv.) and dry THF (30 mL) were added. The reaction mixture was allowed to heated at 50 °C for 48 h. Once the

substrates were consumed, THF was removed under reduced pressure and directly purified by flash column chromatography (*n*-pentane/ ethyl acetate, 1:3) to afford the pure product as yellow oil (**17a'**, 0.87 g, 2.79 mmol, 97%).

^{1}H NMR (400 MHz, $CDCl_3$) δ (ppm): 7.12 (t, J = 7.7 Hz, 1H), 7.02 (d, J = 8.2 Hz, 1H), 6.84-6.75 (m, 3H), 6.63 (t, J = 7.3 Hz, 1H), 6.57 (d, J = 8.2 Hz, 1H), 3.91 (s, 3H), 3.89 (s, 3H), 3.33 (dq, J = 8.7, 4.3 Hz, 1H), 2.95 (s, 3H), 2.92-2.85 (m, 1H), 2.76-2.68 (m, 2H), 2.61-2.53 (m, 1H), 2.04-.91 (m, 3H), 1.82-1.72 (m, 1H). **^{13}C{^{1}H} NMR** (101 MHz, $CDCl_3$) δ 148.9, 147.2, 145.3, 134.7, 128.8, 127.2, 121.7, 120.1, 115.4, 111.6, 111.3, 110.6, 58.5, 56.0, 55.9, 38.2, 33.1, 32.0, 24.4, 23.6.

5. Catalytic Experiments

As a representative example, the hydrogenation of quinoline will be described in this section. For other substrates, the amounts for the substrate, catalyst, solvents, as well as reaction conditions, may be different.

In a glovebox, quinoline (53 equivalents, 0.25 mmol), catalyst (10 mg, 0.0047 mmol of metal), and solvent (heptane, 0.5 mL) were added to the glass inlet of an autoclave. The autoclave was closed, removed from the glovebox, flushed with hydrogen, and pressurized with 50 bar hydrogen. The reaction was performed at 90 °C for 1 hour under stirring (500 rpm). Subsequently, the autoclave was cooled in an ice/water bath and depressurized.

For GC sample preparation, acetone (0.5 mL) was added to the reaction mixture, which was further passed through a syringe filter. Tetradecane (14 mg) was used as an internal standard.

5.1 Determination of turnover number (TON) and frequency (TOF)

For the determination of TOF and TON, the substrate to total Ru loading was increased from 53 to 533. The TOF was determined from after 30 min of reaction under standard conditions (90 °C, 50 bar H_2). After 24 h, quantitative yield of the desired product was reached, corresponding to a TON of 533. To simplify the comparison with other catalytic systems the calculation of the TOF was based on the total Ru-amount.

5.2 Product Isolation

For the isolation of products, reaction mixtures were diluted with acetone and filtered through a syringe filter. In the next step, the solvent was removed under reduced pressure. After drying under vacuum the sample was analyzed using ^{1}H and ^{13}C NMR.

5.3 Catalyst Recycling

In a glovebox, the catalyst (10 mg, containing 0.0047 mmol Ru), substrate (53 equivalents, 0.25 mmol), and solvent (heptane, 0.5 mL) were weighed into the glass inlet of an autoclave. The autoclave was flushed with hydrogen and pressurized with 20 bar of hydrogen. The reaction was performed under stirring (500 rpm) at 50 °C. After 2 h the autoclave was cooled in an ice/water bath and depressurized. To prevent the catalyst oxidation the glass inlet containing the reaction mixture was transferred to a Schlenk tube under a strong argon flow and transferred back into the glovebox. For GC-analysis, first the supernatant was withdrawn with a pipette. The remaining catalyst was washed with 0.5 mL of heptane. Subsequently the new supernatant was taken, passed through a syringe filter and combined with the first phase. For the new catalytic run fresh substrate and heptane were added to the solid catalyst and the autoclave was pressurized with hydrogen and heated under the same conditions as in the first run. Tetradecane (14 mg) was used as the internal standard for GC-FID analysis.

The improved recycling experiments (**Figure S17b**), were performed outside of the glove box under strong argon flow to prevent catalyst oxidation. Catalyst washing was performed with ethanol (2 x 1 mL) and heptane (1 x 1 mL), followed by centrifugation for separation. The heptane and ethanol supernatants were recombined and filtered for GC-analysis. Subsequently quinoline and heptane were added to the reaction vessel and the reaction was prepared for the next run as mentioned before.

5.4 Continuous Flow Experiments

Continuous flow experiments were performed using an H-Cube reactor from ThalesNano. Inside the glovebox $Ru_{50}P_{50}$@SILP (250 mg) and glass beads (500 mg) were loaded into a CatCart reactor cartridge and subsequently sealed (the CatCart press was used to tighten the sealing). A solution including 6-chloroquinoline (**9**) (0.04 $mol \cdot L^{-1}$) in heptane was prepared, kept under an argon atmosphere. Prior to catalysis, the H-Cube system was washed with pure heptane for 10–15 minutes. The CatCart was subsequently inserted in the flow reactor. The reactor was heated to 50 °C (90 °C for productivity investigation) under a continuous flow of heptane 0.7 $mL \cdot min^{-1}$ and hydrogen at 20 bars (50 bar for productivity investigation), until a stable flow and conditions were achieved. The stock solution (0.04 $mol \cdot L^{-1}$ 6-chloroquinoline (**9**) in heptane) was introduced into the system at a flow rate of 0.7 $mL \cdot min^{-1}$, marking the reaction-start. Under the flow samples were

collected every 30 min. which were analyzed via GC with decane as an internal standard. The residence time equals the ratio of the reactor volume to the volumetric flow rate.

5.5 Time Profile

In a glovebox, quinoline (53 equivalents, 0.25 mmol), catalyst (10 mg, 0.0047 mmol), and solvent (heptane, 0.5 mL) were weighed in the glass inlet of an autoclave. The autoclave was sealed, flushed with hydrogen, and pressurized with 20 bars of hydrogen. The reaction was performed under stirring at 500 rpm and 50 °C for different durations. At the end of the reactions, the autoclave was cooled in an ice-water bath and depressurized. For GC-FID analysis, acetone (0.5 mL) was first added to the reaction mixture. The reaction mixture was passed through a syringe fitter. Tetradecane (14 mg) was used as an internal standard.

6. Supplementary Tables and Figures

Table S1. Characterization data for Ru_xP_{100-x}@SILP. Error on the values is typically ~10%, reflecting both sample preparation and measurement.

Sample	Theoretical Ru loading (wt%)	Ru:P ratio SEM-EDX	Ru loading ICP-OES (wt%)	Cl content SEM-EDX (at%)	Cl content ICP-OES (wt%)	NPs size TEM (nm)
$Ru_{25}P_{75}$@SILP	1.6	28:72	-	0	0.08	1.7 ± 0.3
$Ru_{50}P_{50}$@SILP	4.7	54:46	4.4	0	0.03	2.0 ± 0.3
$Ru_{60}P_{40}$@SILP	4.8	61:39	4.3	0.44	0.64	1.9 ± 0.3
Ru@SILP	4.5	100	-	0	0	1.9 ± 0.4 [18]

Table S2. Crystal structure parameters for ruthenium phosphides.

Composition	Space Group	Crystal System	Scattering Paths	Coordination Number	Interatomic Distances	ICSD Index	PDF number
Ru_2P		Orthorhombic	Ru(1)-P	1	2.258	43686	01-089-3031

	Pnma		Ru(1)-P	1	2.301		
			Ru(1)-P	2	2.401		
			Ru(1)-Ru(1)	3	2.75		
			Ru(1)-Ru(2)	4	2.835		
			Ru(1)-Ru(2)	1	2.947		
			Ru(2)-P	1	2.325		
			Ru(2)-P	2	2.546		
			Ru(2)-Ru(1)	1	2.758		
			Ru(2)-P	2	2.817		
			Ru(2)-Ru(1)	4	2.835		
			Ru(2)-Ru(2)	3	2.935		
			Ru(2)-Ru(2)	2	3.209		
RuP	Pnma	Orthorhombic	Ru-P	4	2.309	648015	01-079-4702
			Ru-P	1	2.45		
			Ru-Ru	2	2.664		
			Ru-Ru	2	2.909		
			Ru-P	1	2.939		
			Ru-Ru	2	3.168		
RuP_2	Pnnm	Orthorhombic	Ru-P	6	2.351	42607	01-070-2569
			Ru-Ru	2	2.871		
RuP_3	$P\bar{1}$	Triclinic	Ru(1)-P	1	2.281	62420	01-078-1268
			Ru(1)-P	4	2.375		
			Ru(1)-P	1	2.405		
			Ru(1)-Ru(1)	1	2.869		
			Ru(2)-P	3	2.346		
			Ru(2)-P	2	2.382		

			Ru(2)-P	1	2.402		
			Ru(2)-Ru(2)	1	2.795		
RuP_4	$P\bar{1}$	Triclinic	Ru(1)-P	6	2.367	2492	01-071-0502
			Ru(2)-P	1	2.28		
			Ru(2)-P	3	2.363		
			Ru(2)-P	1	2.387		
			Ru(2)-P	1	2.44		
			Ru(2)-Ru(2)	1	3.576		

Table S3. Fitting results for Ru K-edge EXAFS.

Sample	Scattering path	C.N.	R [Å]	σ2 [Å2]	E0 [eV]	R-factor
Ru foil	Ru-Ru	12 (fixed)	2.68 ± 0.01	0.0034 ± 0.0006	22121.9 ± 2.0	0.0124
Ru25P75@SILP	Ru-P	5.4 ± 0.4	2.30 ± 0.01	0.0077 ± 0.0010	22120.1 ± 0.6	0.0134
	Ru-Ru	2.6 ± 0.8	2.83 ± 0.01	0.0100 ± 0.0026		
Ru50P50@SILP	Ru-P	4.5 ± 0.3	2.31 ± 0.01	0.0073 ± 0.0007	22119.2 ± 0.4	0.0060
	Ru-Ru	4.2 ± 0.5	2.81 ± 0.01	0.0094 ± 0.0009		
Ru60P40@SILP	Ru-P	4.7 ± 0.4	2.33 ± 0.01	0.0090 ± 0.0011	22119.4 ± 0.6	0.0122
	Ru-Ru	3.5 ± 0.6	2.80 ± 0.01	0.0092 ± 0.0013		
Ru50P50@SILP treated	Ru-P	4.5 ± 0.3	2.31 ± 0.01	0.0072 ± 0.0008	22119.8 ± 0.4	0.0079
	Ru-Ru	4.7 ± 0.6	2.80 ± 0.01	0.0112 ± 0.0012		
Ru50P50@SILP -treated-Spent	Ru-P	4.5 ± 0.3	2.31 ± 0.01	0.0070 ± 0.0007	22119.5 ± 0.4	0.0080
	Ru-Ru	4.6 ± 0.6	2.80 ± 0.01	0.0108 ± 0.0011		

S_0^2 = amplitude reduction factor, this value is determined by fitting the EXAFS of Ru foil and used as a fixed parameter for the EXAFS fitting of other Ru samples; C.N. = coordination number; R = interatomic distance; σ^2 = Debye-Waller factor. E_0 = E_0 position in the EXAFS fitting model.

Table S4. Characterization and catalytic performance of as-synthesized and treated $Ru_{50}P_{50}$@SILP.

Catalyst	NPs size (nm)	Ru loading ICP-OES (wt%)	Ru:P ratio ICP-OES	Ru:P ratio SEM-EDX	Yield of **1a** (%)
As-synthesized $Ru_{50}P_{50}$@SILP	2.0 ± 0.3	4.4	51:49	54:46	100
Treated $Ru_{50}P_{50}$@SILP	2.1 ± 0.3	4.2	47:53	55:45	98

Reaction conditions: Catalyst (10 mg) substrate (53 eq.), heptane (0.5 mL), 90 °C, H_2 (50 bar), 16 h, 500 rpm).

Table S5: Catalyst characterization before and after catalysis.

Catalyst	NPs size (nm)	Ru loading ICP-OES (wt%)	Ru:P ratio SEM-EDX	Ru leaching in solution XRF (ppm)
Before catalysis	2.1 ± 0.3	4.2	54:46	0
After 4th-run	2.0 ± 0.3	3.9	54:46	<0.2 ppm

Table S6. Comparison of different heterogenous catalysts for the selective hydrogenation of quinoline.

Catalyst	Solvent	T (°C)	P H_2 (bar)	Recyclability	Continuous flow application?	TOF (h^{-1})
$Ru_{50}P_{50}$@SILP	heptane	90	50	Yes, 5 cycles	Yes. Catalyst stable for at least 7 h	128
Ru@PGS[20]	n-butanol	100	H_2/CO_2 (45 bar)	Yes, 5 cycles	No.	4*
Ru/AC[21]	ethanol	60	5	Not recyclable	No.	47 (42*)
Ru_2P/AC[21]	ethanol	60	5	8 cycles, but at 100% yield.	No.	164 (65*)
RuS_2 /AC[21]	ethanol	60	5	Not recyclable	No.	20 (20*)
nanoRu@hectorite[22]	H_2O	100	30	Not recyclable	No.	n.r.
PEG 4000-stabilized Rh[23]	toluene/heptane (3/1)	100	30	Yes, 10 cycles	No.	320
SiO_2@RF/Pt[24]	toluene	r.t.	1	5 cycles, but at 100% yield.	No.	16*
Co_3O_4– Co/NGr@α-Al_2O_3[25]	toluene	120	20	6 cycles (1st cycle at 100% yield, 6th cycle at 76%)	No.	2.0*
Co@SiO_2[26]	MeOH	80	40	4 cycles, but at 100% yield.	Yes, no detail on catalyst stability	0.5*
Fe(1)/L4(4.5) @C-800[16]	$^iPrOH/H_2O$ (3/1)	130	40	6 cycles, but at 100% yield.	No.	0.3*

*: TOF calculated based on the reported data. TOF given as a function of the metal loading.

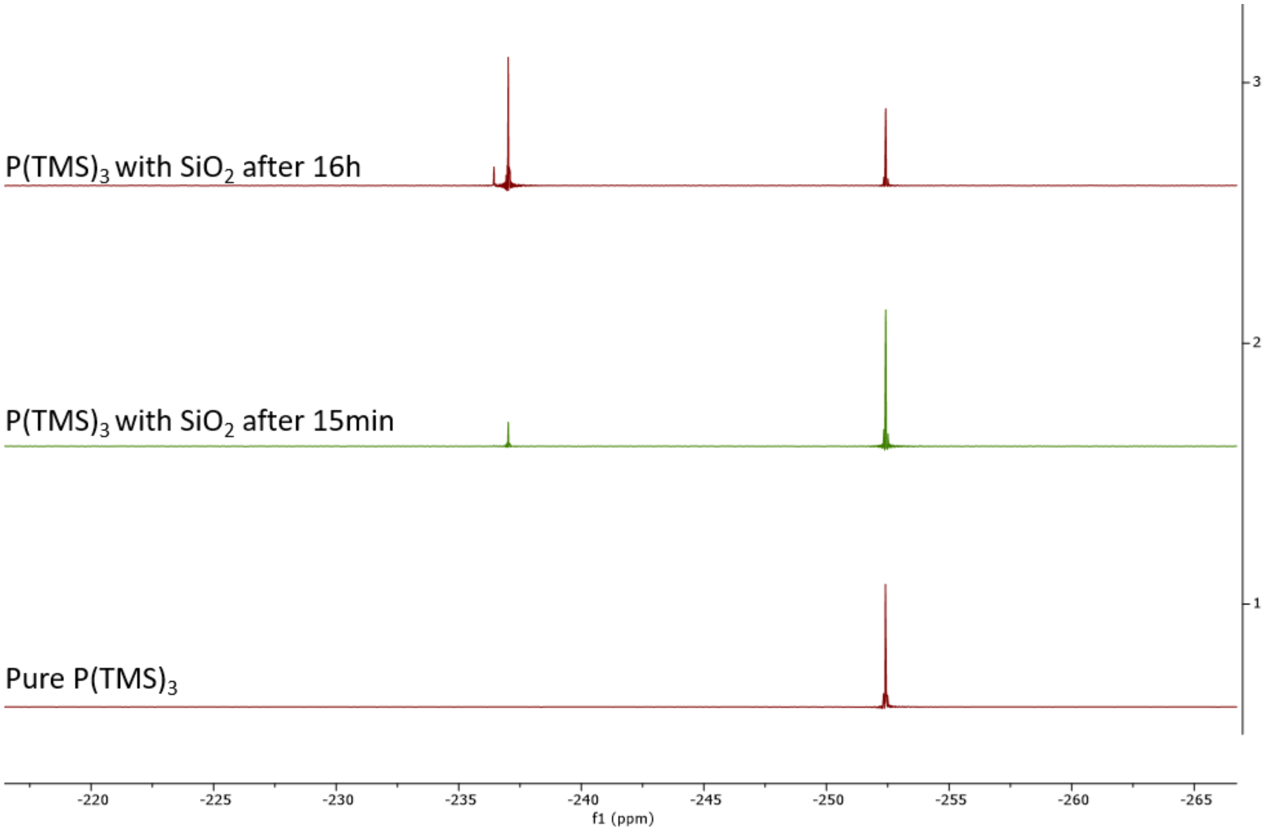

Figure S1. ^{1}H-NMR (400 MHz) spectra of the reaction of $P(SiMe_3)_3$ (0.25 mmol, 64 mg) with SiO_2 (500 mg) in toluene-d_8 at 60 °C.

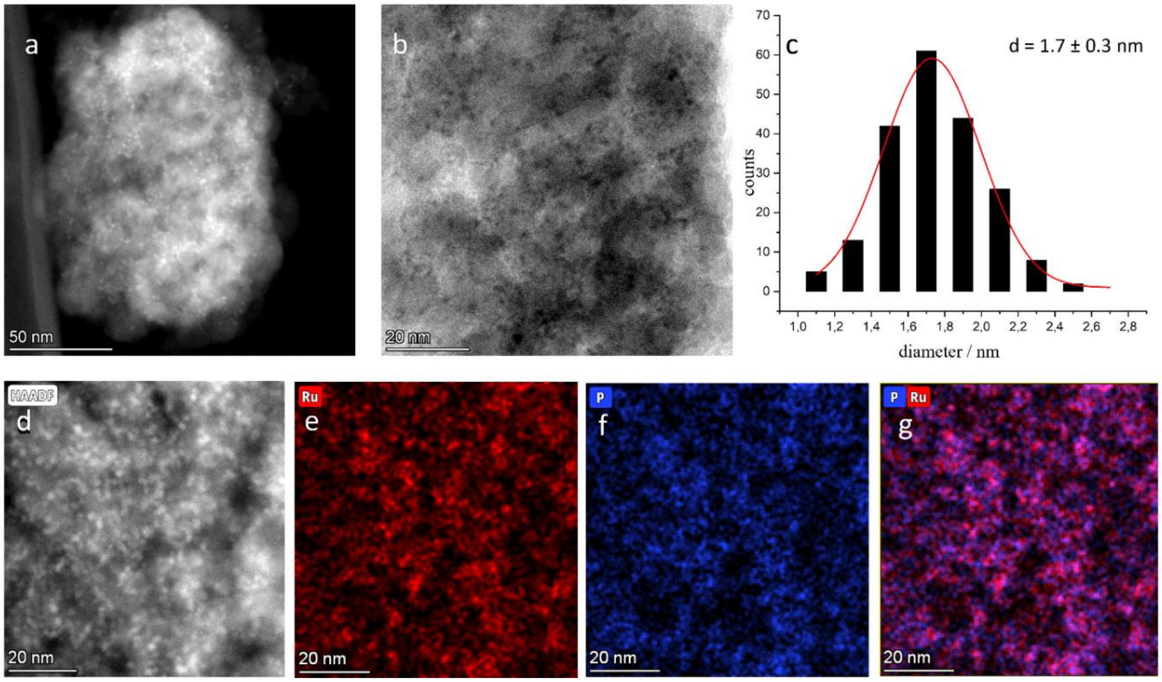

Figure S2. Electron microscopy analysis of $Ru_{25}P_{75}$@SILP. **a**) STEM-HAADF image, **b**) STEM bright field image, **c**) NPs size distribution-histogram, **d**) STEM-HAADF, **e**) STEM-HAADF-EDX elemental mapping of Ru (Lα), **f**) STEM-HAADF-EDX elemental mapping of P (Kα), and **g**) overlay.

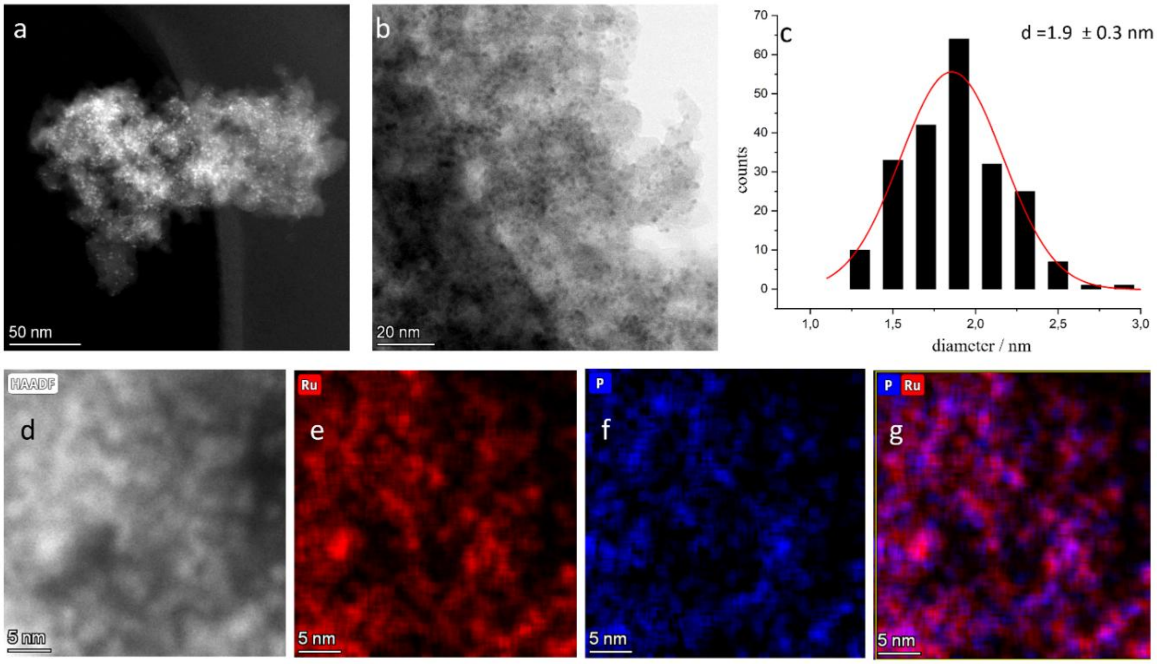

Figure S3. Electron microscopy analysis of $Ru_{60}P_{40}$@SILP. **a**) STEM-HAADF image, **b**) STEM bright field image, **c**) NPs size distribution histogram, **d**) STEM-HAADF, **e**) STEM-HAADF - EDX elemental mapping of Ru (Lα), **f**) STEM-HAADF-EDX elemental mapping of P (Kα), and **g**) overlay.

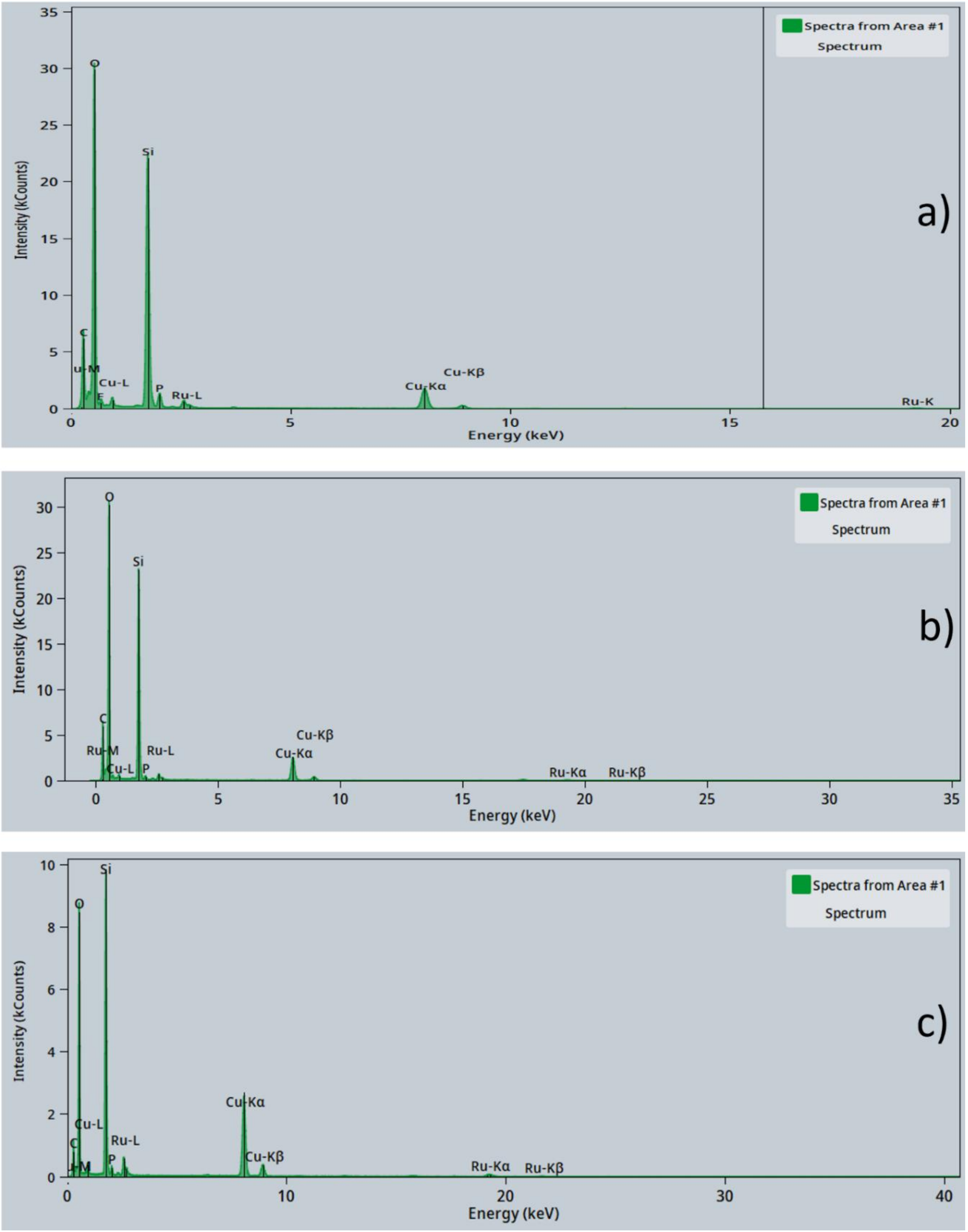

Figure S4. EDS spectra of the three catalysts. **a**) $Ru_{25}P_{75}$@SILP, **b**) $Ru_{50}P_{50}$@SILP, and **c**) $Ru_{60}P_{40}$@SILP.

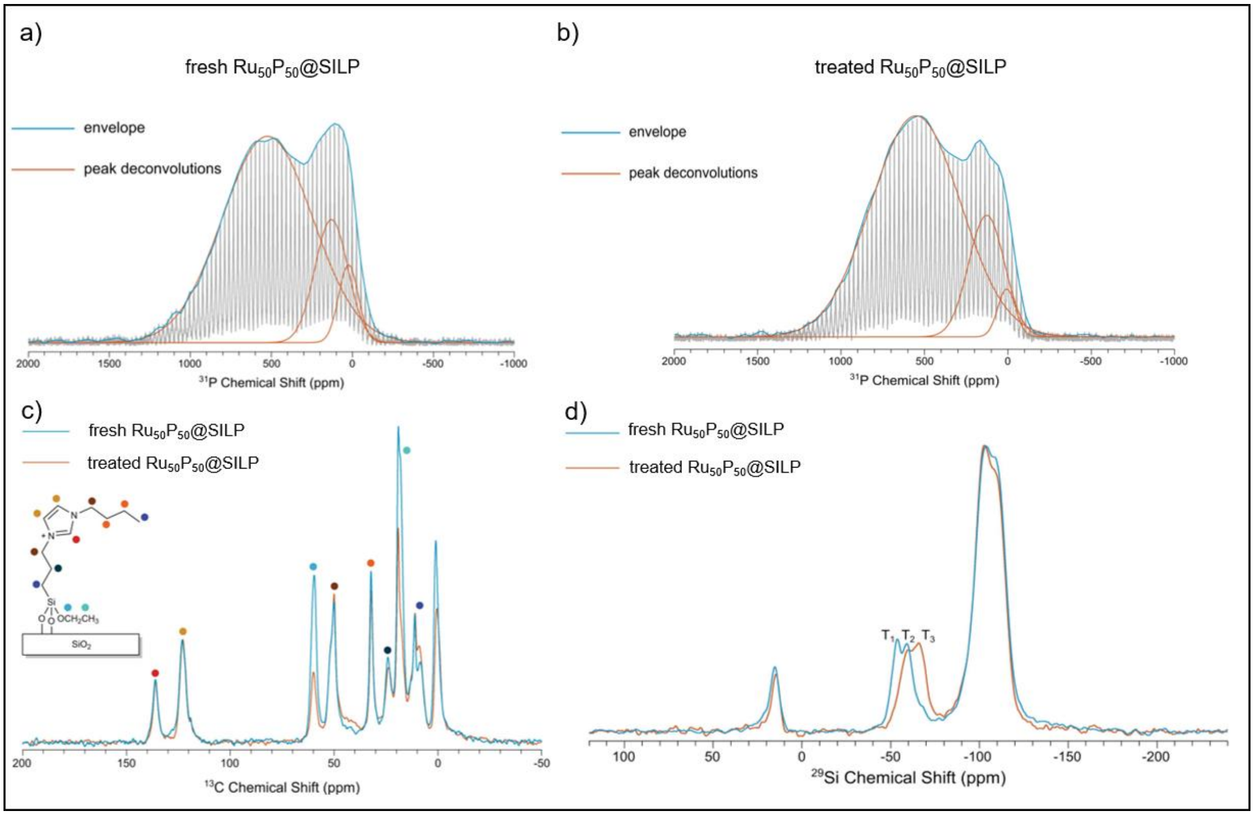

Figure S5. Solid-state NMR: **a**) ^{31}P WURST-CPMG spectrum of fresh $Ru_{50}P_{50}$@SILP. **b**) ^{31}P WURST-CPMG spectrum of treated $Ru_{50}P_{50}$@SILP. In both case line shape simulations are shown as reported previously.[19] **c**) ^{1}H-^{13}C CP-MAS spectrum of both fresh (blue) and treated (orange) $Ru_{50}P_{50}$@SILP; the resonances are assigned to the individual carbon species as indicated by coloured circles. **d**)^{1}H-^{29}Si CP-MAS spectrum of both fresh (blue) and treated (orange) $Ru_{50}P_{50}$@SILP. Resonance assignments are indicated on the spectra.

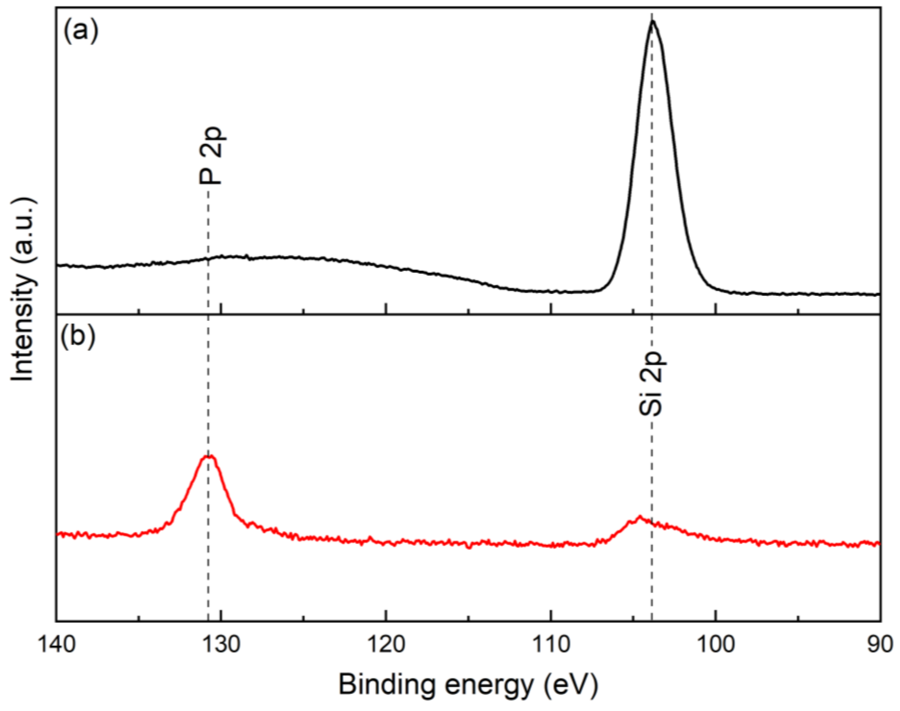

Figure S6. P 2p XPS high-resolution scans of (**a**) supported, and (**b**) isolated $Ru_{60}P_{40}$ NPs.

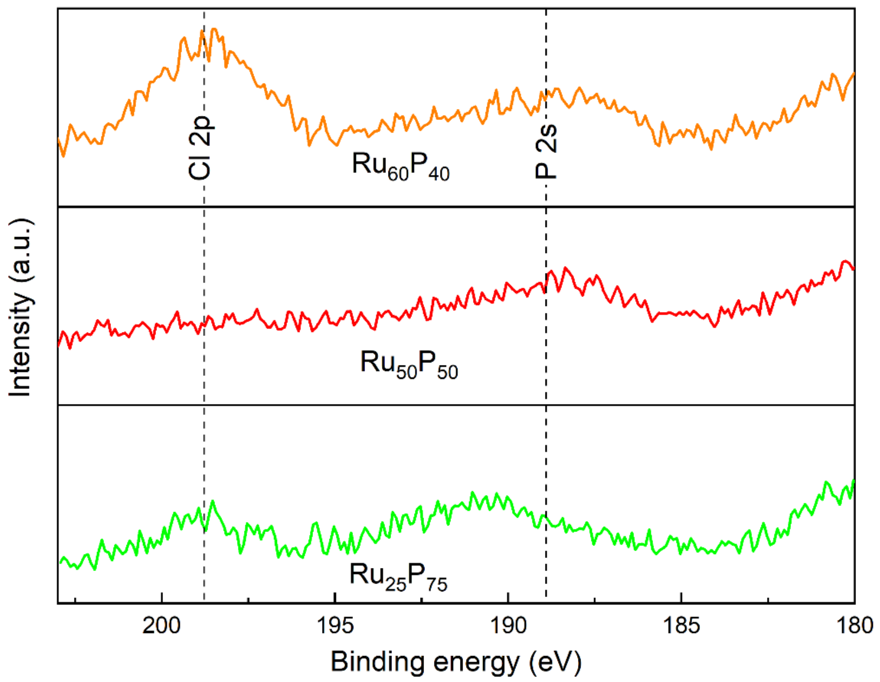

Figure S7. P 2s and Cl 2p XPS high-resolution scans of Ru_xP_{100-x}@SILPs samples.

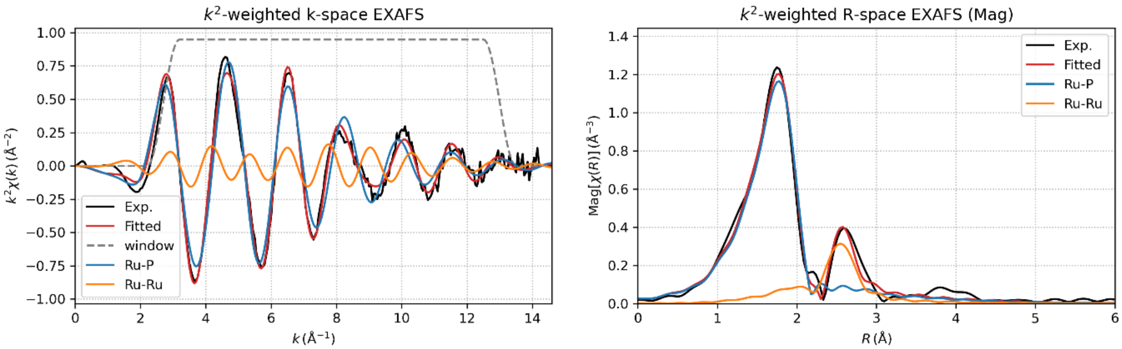

Figure S8. Fitting results of k^2-weighted k-space and R-space FT-EXAFS spectra of $Ru_{25}P_{75}$@SILP. The experimental EXAFS spectra and fitted spectra are plotted in black and red curves, respectively. The R-space spectra are plotted without phase correction.

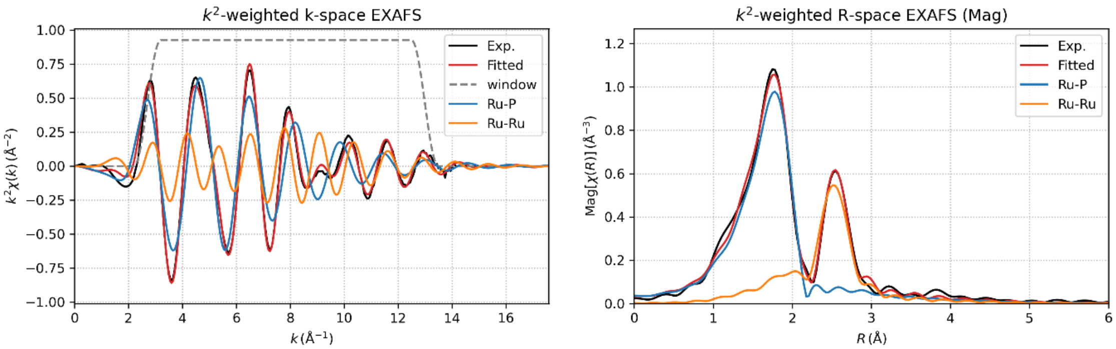

Figure S9. Fitting results of k^2-weighted k-space and R-space FT-EXAFS spectra of $Ru_{50}P_{50}$@SILP. The experimental EXAFS spectra and fitted spectra are plotted in black and red curves, respectively. The R-space spectra are plotted without phase correction.

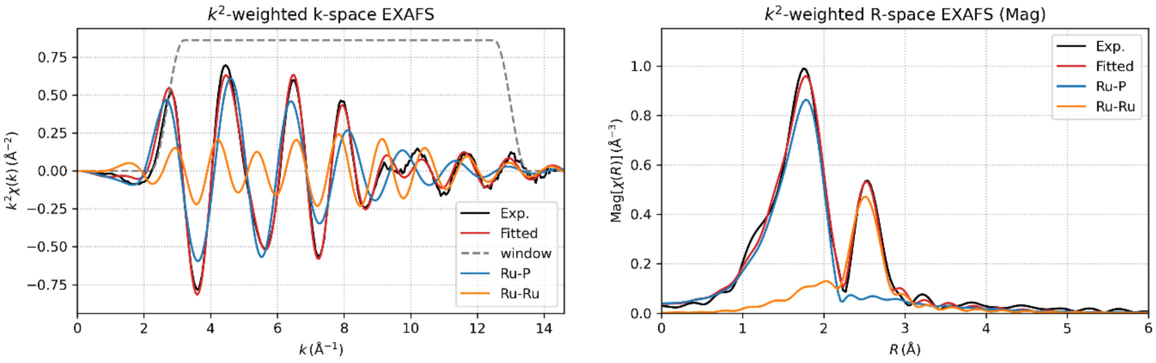

Figure S10. Fitting results of k^2-weighted k-space and R-space FT-EXAFS spectra of $Ru_{60}P_{40}$@SILP. The experimental EXAFS spectra and fitted spectra are plotted in black and red curves, respectively. The R-space spectra are plotted without phase correction.

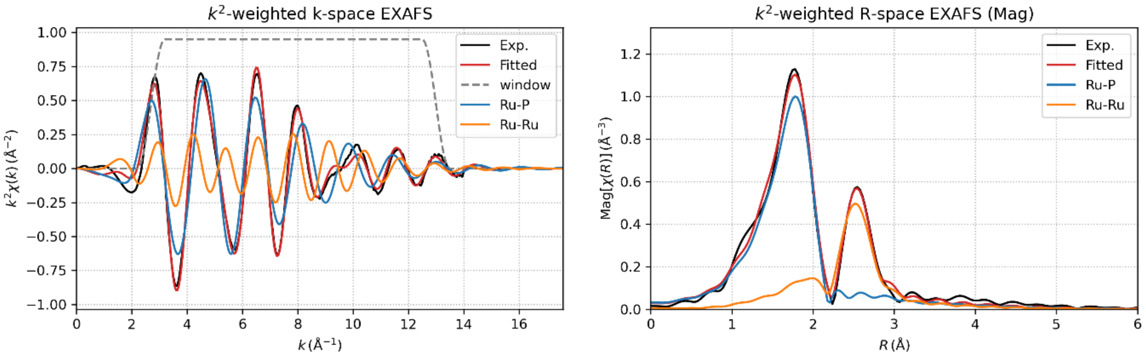

Figure S11. Fitting results of k^2-weighted k-space and R-space FT-EXAFS spectra of $Ru_{50}P_{50}$@SILP-PHT. The experimental EXAFS spectra and fitted spectra are plotted in black and red curves, respectively. The R-space spectra are plotted without phase correction.

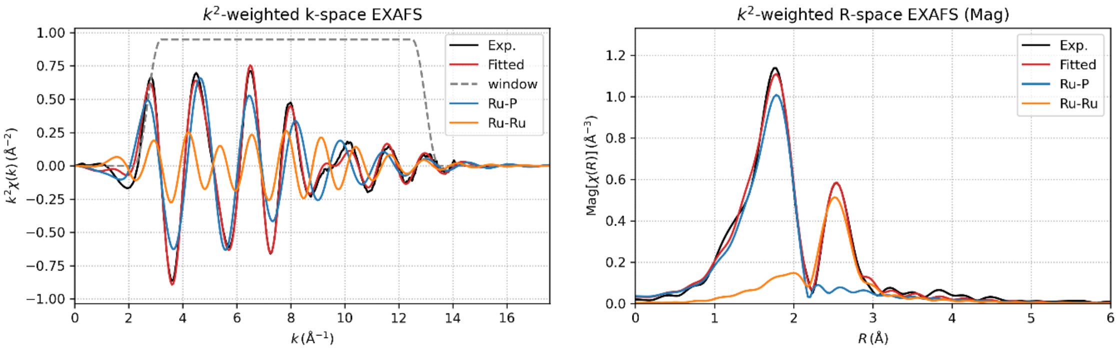

Figure S12. Fitting results of k^2-weighted k-space and R-space FT-EXAFS spectra of $Ru_{50}P_{50}$@SILP-PHT-Spent. The experimental EXAFS spectra and fitted spectra are plotted in black and red curves, respectively. The R-space spectra are plotted without phase correction.

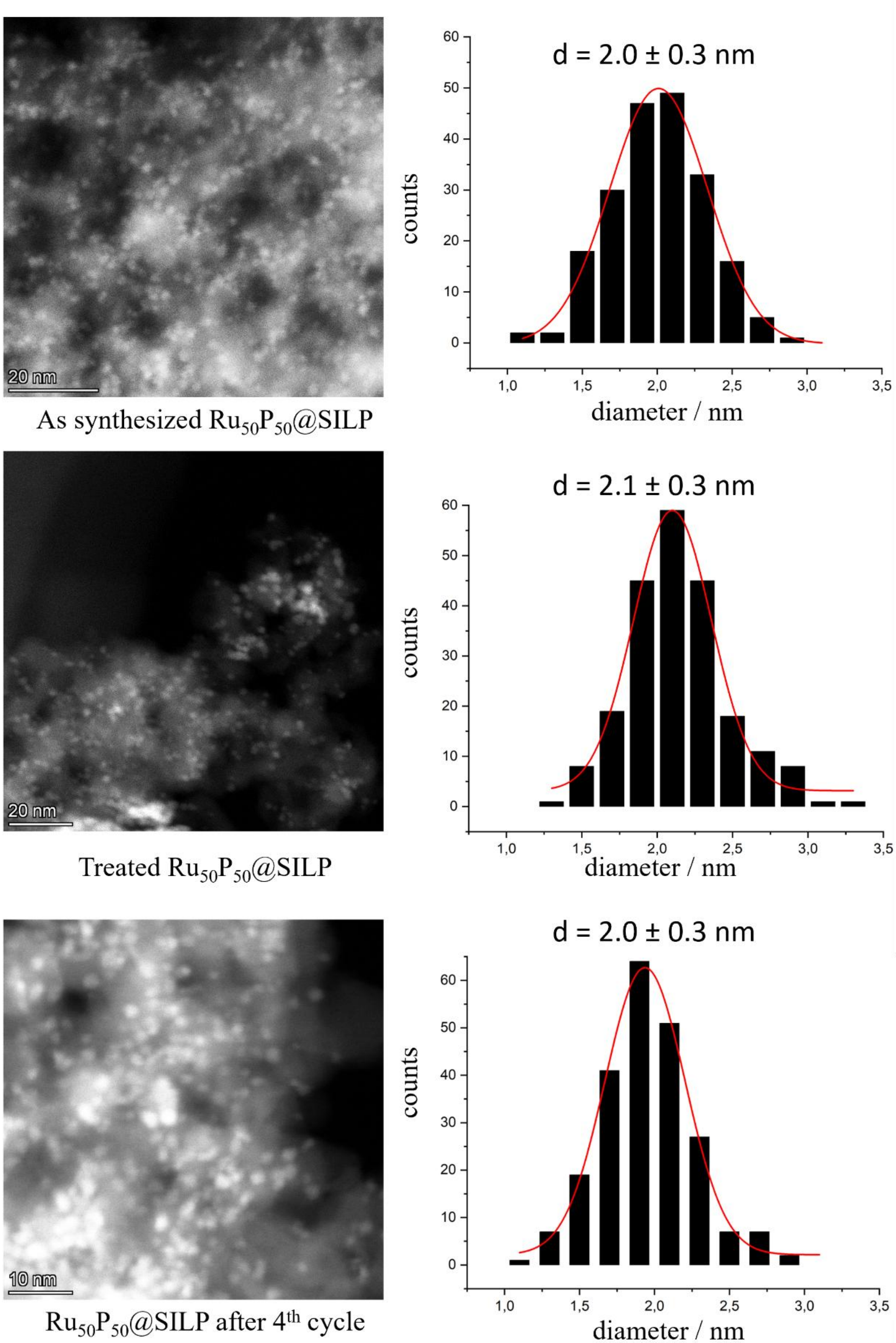

Figure S13. STEM-HAADF images of as-synthesized, treated, and spent $Ru_{50}P_{50}$@SILP catalysts.

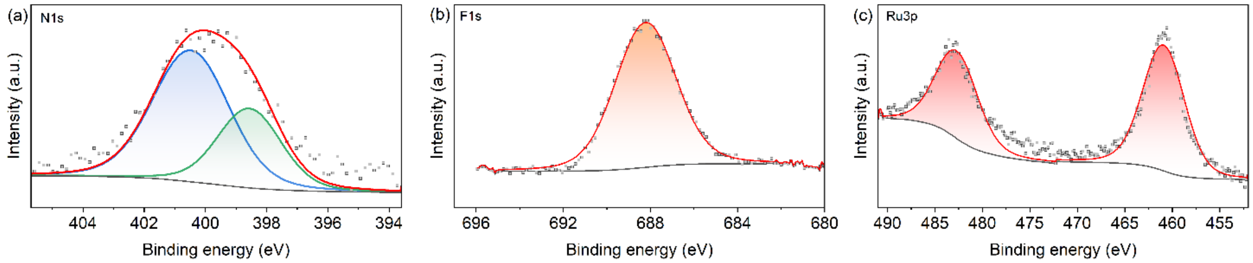

Figure S14. High-resolution XPS spectra of (**a**) N1s and (**b**) F1s and (**c**) Ru3p in treated $Ru_{50}P_{50}$@SILP.

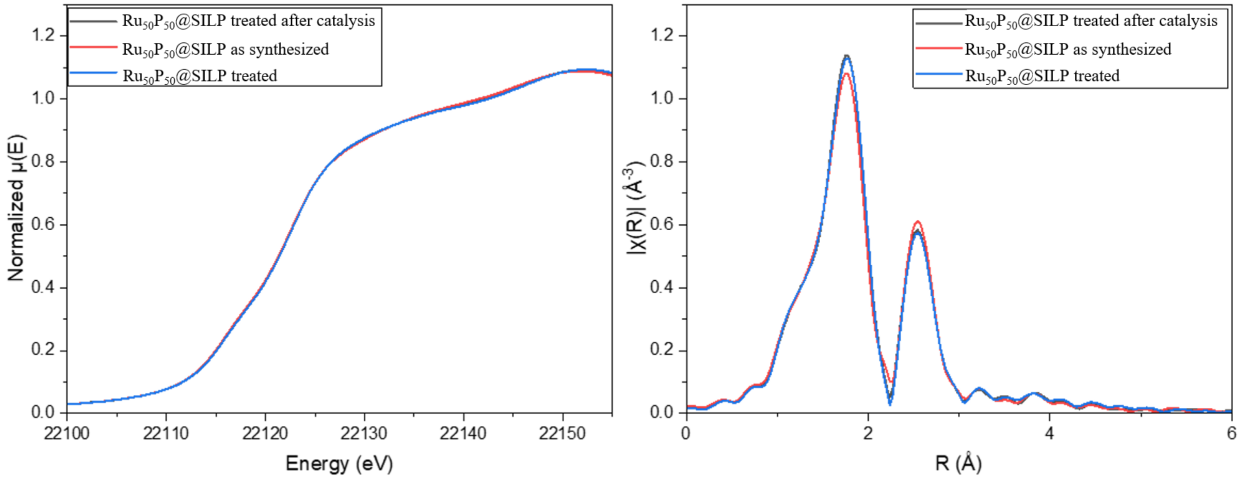

Figure S15. Ru K-edge XAS of as synthesized $Ru_{50}P_{50}$@SILP (red), treated-$Ru_{50}P_{50}$@SILP (blue) and spent $Ru_{50}P_{50}$@SILP (black). (left) Normalized XANES spectra and (right) k^2-weighted R-space Fourier transformed EXAFS (plotted without phase correction).

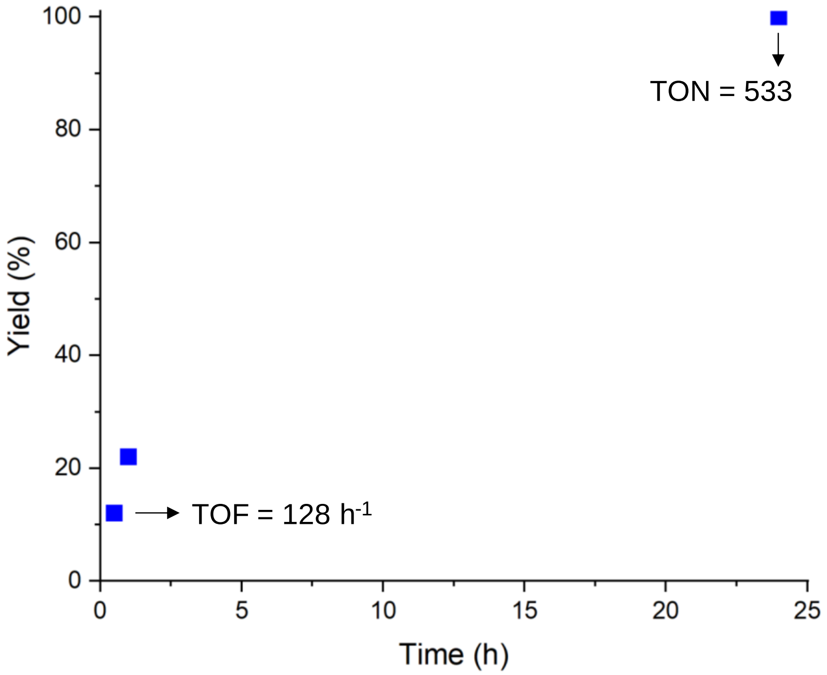

Figure S16: Data points used to determine turnover frequency (TOF) and turnover number (TON). Reaction conditions: $Ru_{50}P_{50}$@SILP (10 mg), quinoline (533 eq. with respect to total Ru loading), 90 °C, 50 bar H_2, 500 rpm.

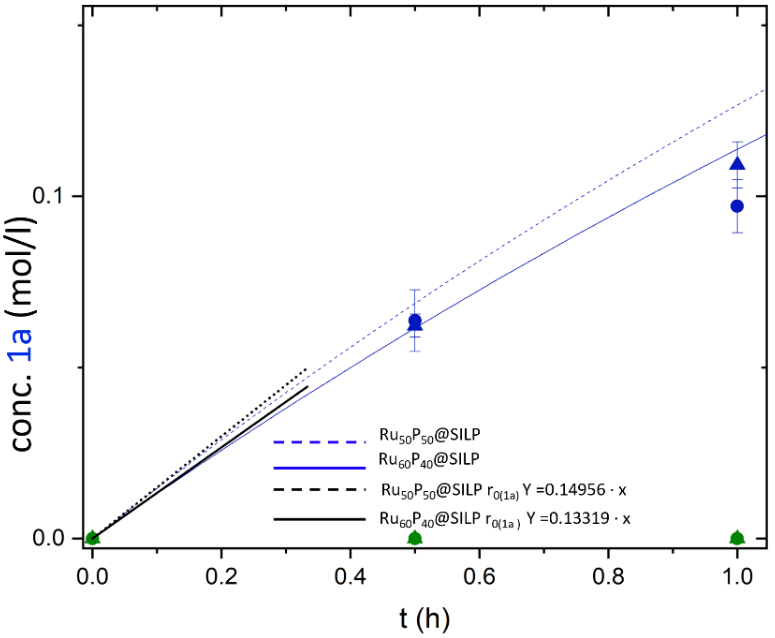

Figure S17. Initial rate determination for both catalyst from the time profiles for the hydrogenation of **1** using $Ru_{50}P_{50}$@SILP and $Ru_{60}P_{40}$@SILP under adapted conditions (Catalyst (10 mg) substrate (53 eq.), heptane (0.5 mL), 50 °C, H_2 (20 bar), 500 rpm). Each data point is the average of 2-3 experiments and error bars represent standard deviations.

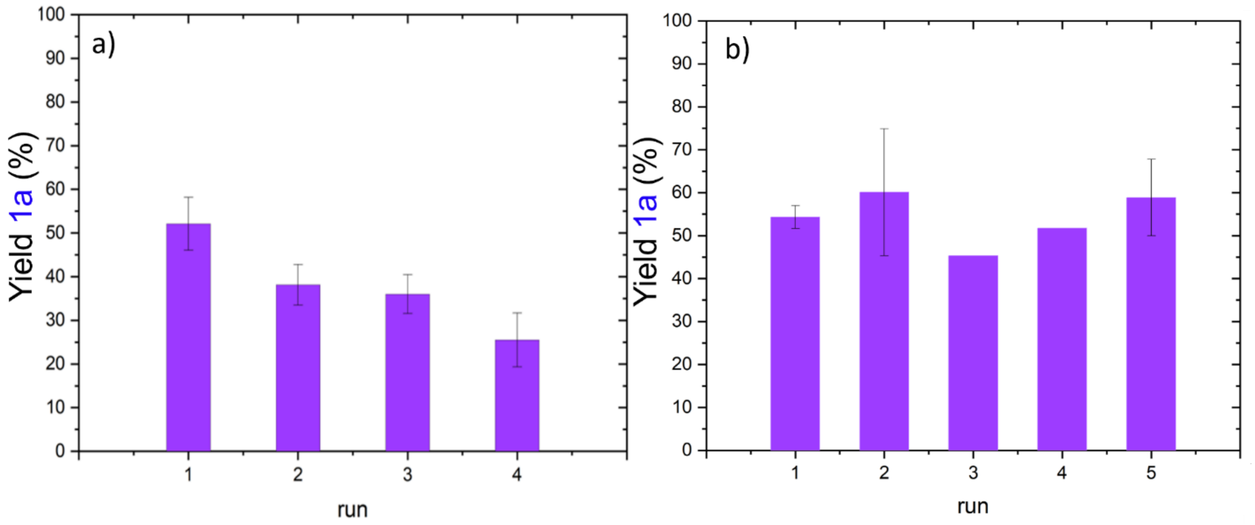

Figure S18. Recycling experiments of selective hydrogenation of quinoline (**1**). **a**) Catalyst was washed with heptane. **b**) Catalyst was washed with ethanol. Reaction conditions: $Ru_{50}P_{50}$@SILP (20 mg), substrate (64 mg, 55 eq. related to Ru metal), 20 bar H_2, 50 °C, 2 h, 500 rpm. The yields were determined via GC-FID, using tetradecane as the internal standard. Each data point in an average of 2-3 experiments and error bars represent standard deviation.

7. Box 1. Green Chemistry Metrics

Following formula were used to determine green chemistry metrics:[27]

- E Factor = mass of total waste/mass of product [(total waste (kg)/total product (kg)]
- Atom economy = molecular weight of product/sum of molecular weights of all starting materials
- Atom efficiency = (% yield) × atom economy
- Reaction mass efficiency: mass of product/total mass of reactants
- Carbon efficiency = (amount of carbon in product/amount of total carbon present in reactants) × 100

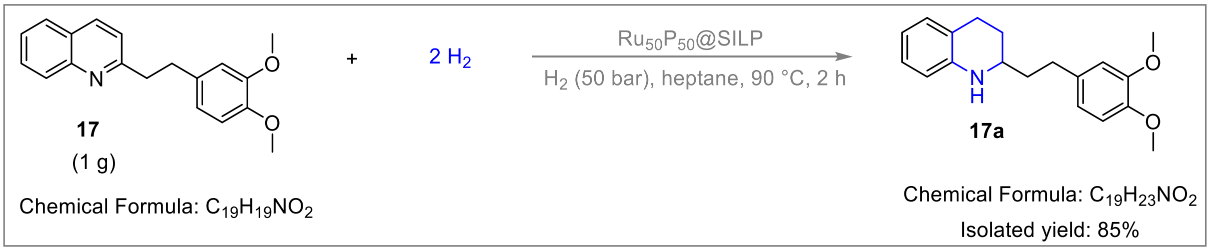

Substrate/product	Name	Mass	Mol. weight	Remarks
Reactant **17**	2-(3,4-dimethoxyphenethyl)quinoline	1.002 g	293.140	-
Reactant (H_2)	Dihydrogen	0.004 g (50 bar)	2.016	Excess hydrogen can be recycled
Solvent for reaction	Heptane	5.0 mL (3.398 g)	100.125 (d = 0.6795 g cm^{-3})	-
Recycled solvent	Heptane	4.5 mL (3.058 g)	-	>90% recovery
Washing solvent	Acetone	2.0 mL (1.569 g)	58.042 (d = 0.7845 g/cm^3)	-
Recycled washing solvent	Acetone	1.5 mL (1.177 g)	-	>75% recovery
Catalyst	$Ru_{50}P_{50}$@SILP	0.138 g	-	Recycled and reused
Product **17a**	2-(3,4-dimethoxyphenethyl)-1,2,3,4-tetrahydroquinoline	0.863 g	297.173	-

E-factor = {1.002 + 0.004 + (3.398 – 3.058 = 0.34) + (1.569 – 1.177 = 0.392)} g / 0.863 g

= 1.738 g / 0.863 g = **2.01**

Atom economy = [297.1729/297.173] x 100 = **100%**

Atom efficiency = **85%**

Carbon efficiency = **100%**

Reaction mass efficiency = [0.863 g/ (1.002 + 0.004) g] x 100 = 0.863/1.006 = **86%**

8. NMR Data for Isolated Products.

1,2,3,4-Tetrahydroquinoline (1a). ^{1}**H NMR** (400 MHz, $CDCl_3$) δ (ppm) 7.0-6.95 (m, 2H), δ 6.62 (td, J = 7.8, 1.0 Hz, 1H), 6.49 (dd, J = 7.8, 0.9 Hz, 1H), 3.63 (s, 1H), 3.33-3.30 (m, 2H), 2.78 (t, J = 6.5 Hz, 2H), 1.99-1.93 (m, 2H). ^{13}**C{^{1}H} NMR** (101 MHz, $CDCl_3$) δ (ppm) 144.9, 129.6, 126.8, 121.5, 117.0, 114.3, 42.1, 27.1, 22.3.

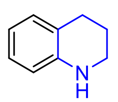

1a

6-Isopropyl-1,2,3,4-tetrahydroquinoline (2a). ^{1}**H NMR** (400 MHz, $CDCl_3$) δ (ppm) 6.88-6.84 (m, 2H), 6.47 (d, J = 8.0 Hz, 1H), 3.66 (s, 1H), 3.31-3.28 (m, 2H), 2.84-2.74 (m, 3H), 1.99-1.93 (m, 2H), 1.23 (d, J = 6.9 Hz, 6H). ^{13}**C{^{1}H} NMR** (101 MHz, $CDCl_3$) δ (ppm) 142.8, 137.8, 127.6, 124.7, 121.6, 114.6, 42.3, 33.3, 27.1, 24.4, 22.5.

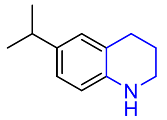

2a

6-Tertbutyl-1,2,3,4-tetrahydroquinoline (3a). ^{1}**H NMR** (400 MHz, $CDCl_3$) δ (ppm) 7.06-7.01 (m, 2H), 6.49 (d, J = 8.2 Hz, 1H), 3.67 (s, 1H), 3.32-3.30 (m, 2H), 2.81 (t, J = 6.5 Hz, 2H), 2.01-1.95 (m, 2H), 1.31 (s, 9H). ^{13}**C{^{1}H} NMR** (101 MHz, $CDCl_3$) δ (ppm) 142.5, 140.0, 126.4, 123.8, 121.1, 114.3, 42.2, 33.9, 31.7, 27.3, 22.6.

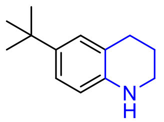

3a

6-Methoxy-1,2,3,4-tetrahydroquinoline (4a). ^{1}**H NMR** (400 MHz, $CDCl_3$) δ (ppm) 6.62-6.57 (m, 2H), 6.46 (d, J = 8.5 Hz, 1H), 3.74 (s, 3H), 3.42 (s, 1H), 3.27-3.24 (m, 2H), 2.76 (t, J = 6.5 Hz, 2H), 1.96-1.90 (m, 2H). ^{13}**C{^{1}H} NMR** (101 MHz, $CDCl_3$) δ (ppm) 151.9, 138.9, 123.0, 115.7, 115.0, 113.0, 55.9, 42.4, 27.3, 22.5.

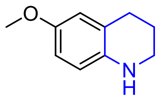

4a

6-(Trifluoromethyl)-1,2,3,4-tetrahydroquinoline (5a). ^{1}**H NMR** (400 MHz, $CDCl_3$) δ (ppm) 7.20-7.18 (m, 2H), 6.45 (d, J = 9.0 Hz, 1H), 4.0 (s, 1H), 3.36-3.33 (m, 2H), 2.78 (t, J = 6.4 Hz, 2H), 1.97-1.91 (m, 2H). ^{13}**C{^{1}H} NMR** (101 MHz, $CDCl_3$) δ (ppm) 147.4, 126.6 (q, J = 3.8 Hz),125.2 (q, J = 270.4 Hz),124.1 (q, J = 3.9 Hz), 120.7, 118.2 (q, J = 32.3 Hz), 113.2, 41.8, 27.0, 21.5.

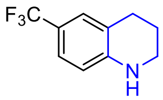

5a

6-Fluoro-1,2,3,4-tetrahydroquinoline (8a). ^{1}**H NMR** (400 MHz, $CDCl_3$) $\underline{\delta}$ (ppm) 6.71-6.66 (m, 2H), 6.41 (dd, J = 9.4, 4.9 Hz, 1H), 3.52 (s, 1H), 3.28-3.25 (m, 2H), 2.75 (t, J = 6.5 Hz, 2H), 1.95-1.89 (m, 2H). ^{13}**C{^{1}H} NMR** (101 MHz, $CDCl_3$) $\underline{\delta}$ (ppm) 155.6 (d, J = 234.5 Hz), 141.0 (d, J = 1.9 Hz), 122.9 (d, J = 6.6 Hz), 115.7 (d, J = 21.6 Hz), 115.0 (d, J = 7.6 Hz), 113.3 (d, J = 22.3 Hz), 42.2, 27.1 (d, J = 1.4 Hz), 22.1.

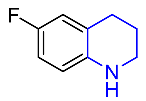

8a

6-Chloro-1,2,3,4-tetrahydroquinoline (9a). ^{1}**H NMR** (400 MHz, $CDCl_3$) $\underline{\delta}$ (ppm) 6.92-6.89 (m, 2H), 6.39 (dd, J = 7.8, 1.1 Hz, 1H), 3.69 (s, 1H), 3.30-3.27 (m, 2H), 2.73 (t, J = 6.4 Hz, 1H), 1.95-1.89 (m, 2H). ^{13}**C{^{1}H} NMR** (101 MHz, $CDCl_3$) $\underline{\delta}$ (ppm) 143.3, 129.1, 126.6, 123.0, 121.3, 115.2, 42.0, 27.0, 21.8.

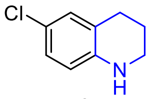

9a

6-Bromo-1,2,3,4-tetrahydroquinoline (10a). ^{1}**H NMR** (400 MHz, $CDCl_3$) $\underline{\delta}$ (ppm) 7.06-7.02 (m, 2H), 6.35 (d, J = 8.3 Hz, 1H), 3.88 (s, 1H), 3.30-3.27 (m, 2H), 2.73 (t, J = 6.4 Hz, 2H), 1.94-1.88 (m, 2H). ^{13}**C{^{1}H} NMR** (101 MHz, $CDCl_3$) $\underline{\delta}$ (ppm) 143.6, 132.0, 129.5, 123.6, 115.7, 108.5, 41.9, 26.9, 21.7.

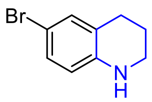

10a

2-Methyl-1,2,3,4-tetrahydroquinoline (14a). ^{1}**H NMR** (400 MHz, $CDCl_3$) $\underline{\delta}$ (ppm) 7.01-6.96 (m, 2H), 6.63 (td, J = 7.4, 1.2 Hz, 1H), 6.50 (dd, J = 8.3, 1.2 Hz, 1H), 3.59 (s, 1H), 3.41 (dtd, J = 12.6, 6.3, 2.9 Hz, 1H), 2.91-2.82 (m, 1H), 2.78-2.72 (m, 1H), 1.98-1.92 (m, 1H), 1.66-1.56 (m, 1H), 1.23 (d, J = 6.3 Hz, 3H). ^{13}**C{^{1}H} NMR** (101 MHz, $CDCl_3$) $\underline{\delta}$ (ppm) 144.8, 129.4, 126.8, 121.2, 117.1, 114.1, 47.3, 30.2, 26.7, 22.7.

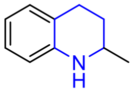

14a

3-Methyl-1,2,3,4-tetrahydroquinoline (15a). ^{1}**H NMR** (400 MHz, $CDCl_3$) $\underline{\delta}$ (ppm) 7.01-6.96 (m, 2H), 6.63 (td, J = 7.3, 1.2 Hz, 1H), 6.51 (dd, J = 7.3, 1.2 Hz, 1H), 3.75 (s, 1H), 3.29 (ddd, J = 10.9, 3.7, 2.0 Hz, 1H), 2.94-2.89 (m, 1H), 2.80 (ddd, J = 16.0, 4.0, 2.0 Hz, 1H), 2.49-2.42 (m, 1H), 2.13-2.04 (m, 1H), 1.07 (d, J = 6.6 Hz, 3H). ^{13}**C{^{1}H} NMR** (101 MHz, $CDCl_3$) $\underline{\delta}$ (ppm) 144.4, 129.6, 126.8, 121.2, 117.1, 114.0, 48.9, 35.6, 27.3, 19.2.

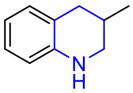

15a

(6-Methoxy-3,4-dihydroquinolin-1(2H)-yl)(3,4,5-trimethoxyphenyl)methanone (4a'). **^{1}H NMR** (400 MHz, $CDCl_3$) *δ* (ppm) 6.68 (s+s, 2H), 6.57 (s, 2H), 6.48 (d, *J* = 6.5 Hz, 1H), 3.86 (t, *J* = 6.5 Hz, 2H), 3.82 (s, 3H), 3.73 (s, 3H), 3.68 (s, 6H), 2.79 (t, *J* = 6.6 Hz, 2H), 2.02 (p, *J* = 6.5 Hz, 2H). **^{13}C{^{1}H} NMR** (101 MHz, $CDCl_3$) *δ* (ppm) 169.5, 156.7, 152.8, 139.6, 133.1, 132.7, 131.3, 126.4, 113.2, 111.5, 106.3, 61.0, 56.1, 55.5, 44.7, 27.3, 24.3.

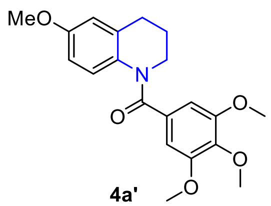

4a'

6,7-Dimethoxy-1-methyl-1,2,3,4-tetrahydroisoquinoline (19a). **^{1}H NMR** (400 MHz, $CDCl_3$) *δ* (ppm) 6.60 (s, 1H), 6.54 (s, 1H), 4.02 (q, *J* = 6.7 Hz, 1H), 3.83 (s, 3H), 3.82 (s, 3H), 3.25-3.20 (m, 1H), 3.0-2.94 (m, 1H), 2.81-2.72 (m, 1H), 2.65-2.59 (m, 1H), 2.01 (s, 1H), 1.42 (d, *J* = 6.6 Hz, 3H). **^{13}C{^{1}H} NMR** (101 MHz, $CDCl_3$) *δ* (ppm) 147.4, 147.3, 132.3, 126.8, 111.8, 109.0, 56.0, 55.9, 51.3, 41.8, 29.5, 22.9.

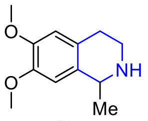

19a

5,10-Dihydrophenazine (21a). **^{1}H NMR (crude mixture)** (400 MHz, $(CD_3)_2SO$) *δ* (ppm) 8.30 - 8.27 (m, 0.11H, phenazine), 7.99 - 7.97 (m, 0.11H, phenazine), 7.29 (brs., 2H, product-amine), 6.27-6.25 (m, 4H, product), 6.04-5.99 (m, 4H, product).

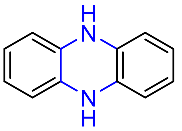

21a

Indolin-1-yl(3,4,5-trimethoxyphenyl)methanone (28a). **^{1}H NMR** (400 MHz, $CDCl_3$) *δ* (ppm) 7.91 (s, 1H), 7.19 (d, *J* = 7.5 Hz, 1H), 7.10 (s, 1H), 6.99 (t, *J* = 7.5 Hz, 1H), 6.76 (s, 2H), 4.09 (t, *J* = 8.3 Hz, 2H), 3.87 (s, 3H), 3.84 (s, 6H), 3.10 (t, *J* = 8.3 Hz, 2H). **^{13}C{^{1}H} NMR** (101 MHz, $CDCl_3$) *δ* (ppm) 168.6, 153.4, 142.5, 139.7, 132.4, 127.2, 125.0, 124.0, 116.9, 104.5, 61.0, 56.3, 50.6, 28.0.

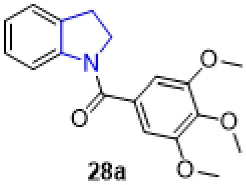

28a

9. NMR Spectra.

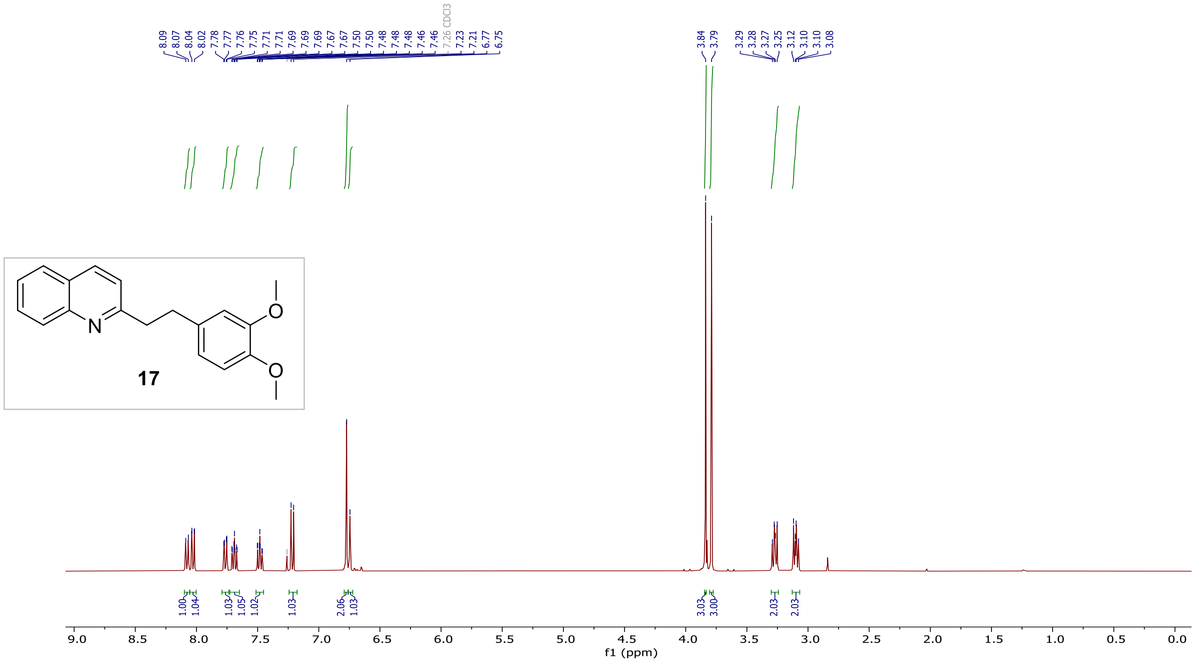

Figure S19. ^{1}H NMR (400 MHz, $CDCl_3$) spectrum of 2-(3, 4-dimethoxyphenethyl)quinoline (**17**).

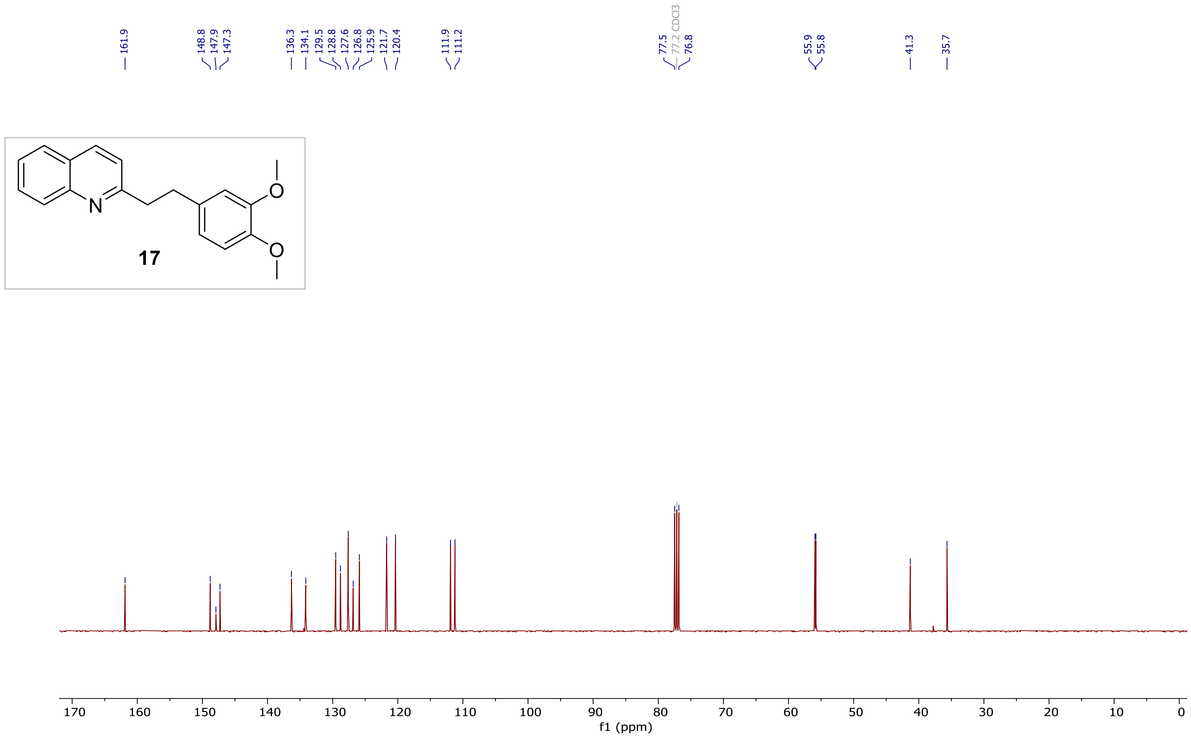

Figure S20. $^{13}C\{^{1}H\}$ NMR (101 MHz, $CDCl_3$) spectrum of 2-(3, 4-dimethoxyphenethyl)quinoline (**17**).

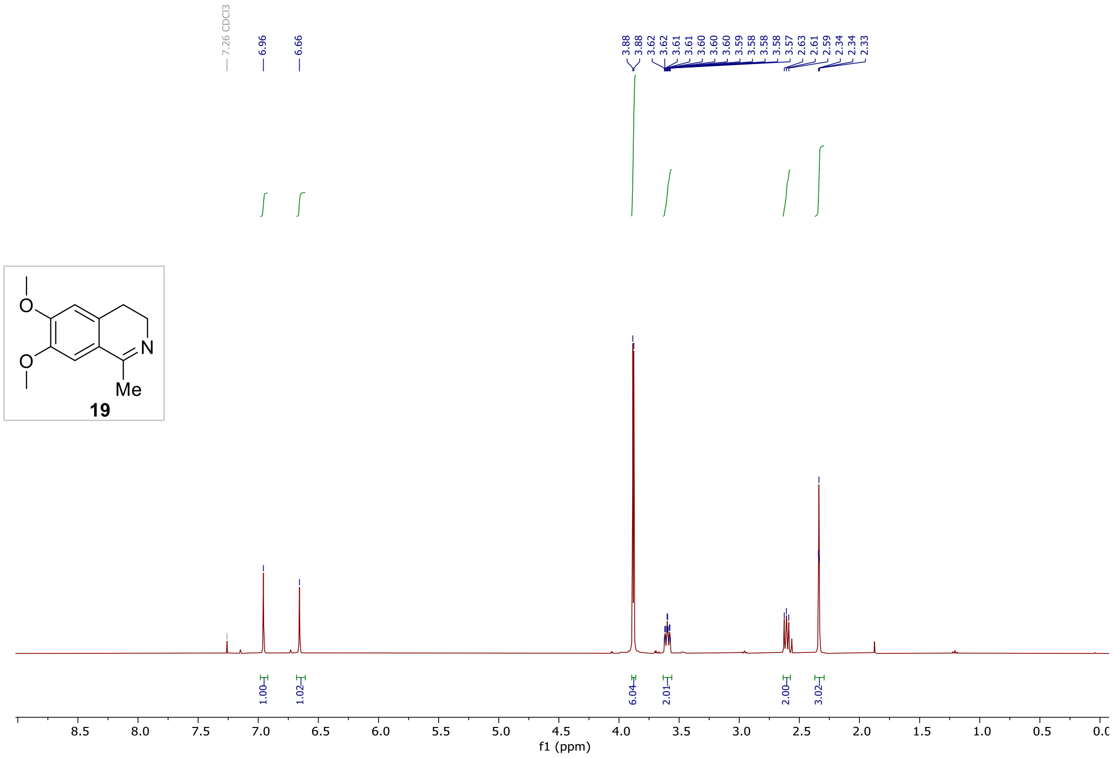

Figure S21. ^{1}H NMR (400 MHz, $CDCl_3$) spectrum of 6,7-dimethoxy-1-methyl-3,4-dihydroisoquinoline (**19**).

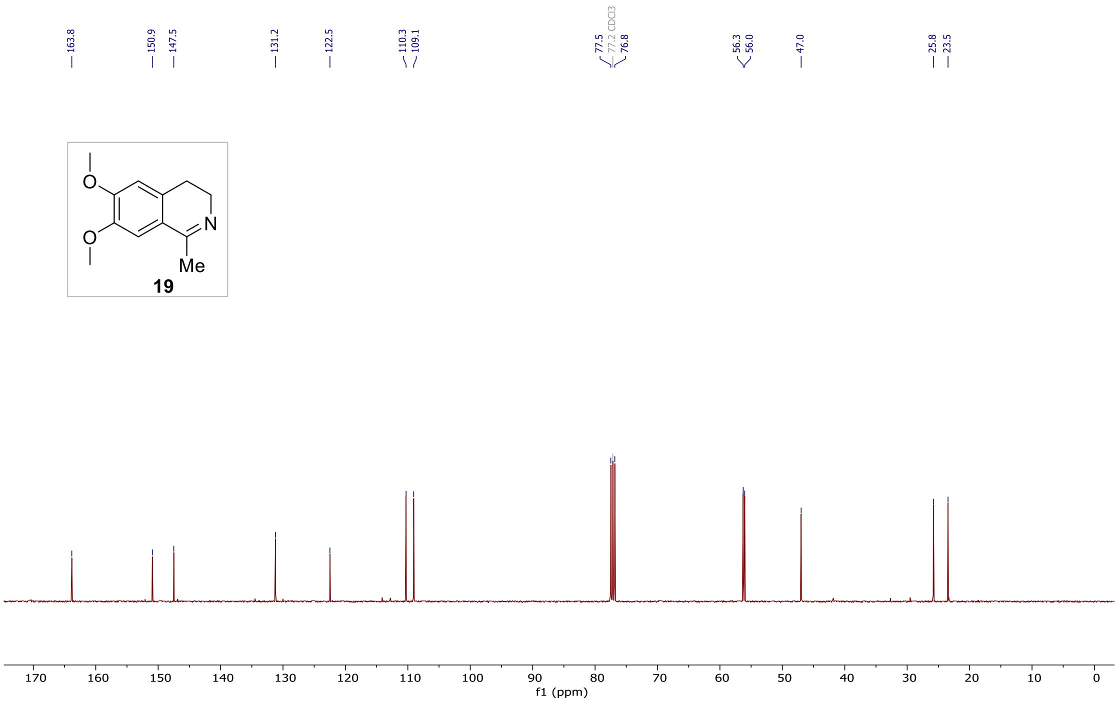

Figure S22. $^{13}C\{^{1}H\}$ (101 MHz, $CDCl_3$) NMR spectrum of 6,7-dimethoxy-1-methyl-3,4-dihydroisoquinoline (**19**).

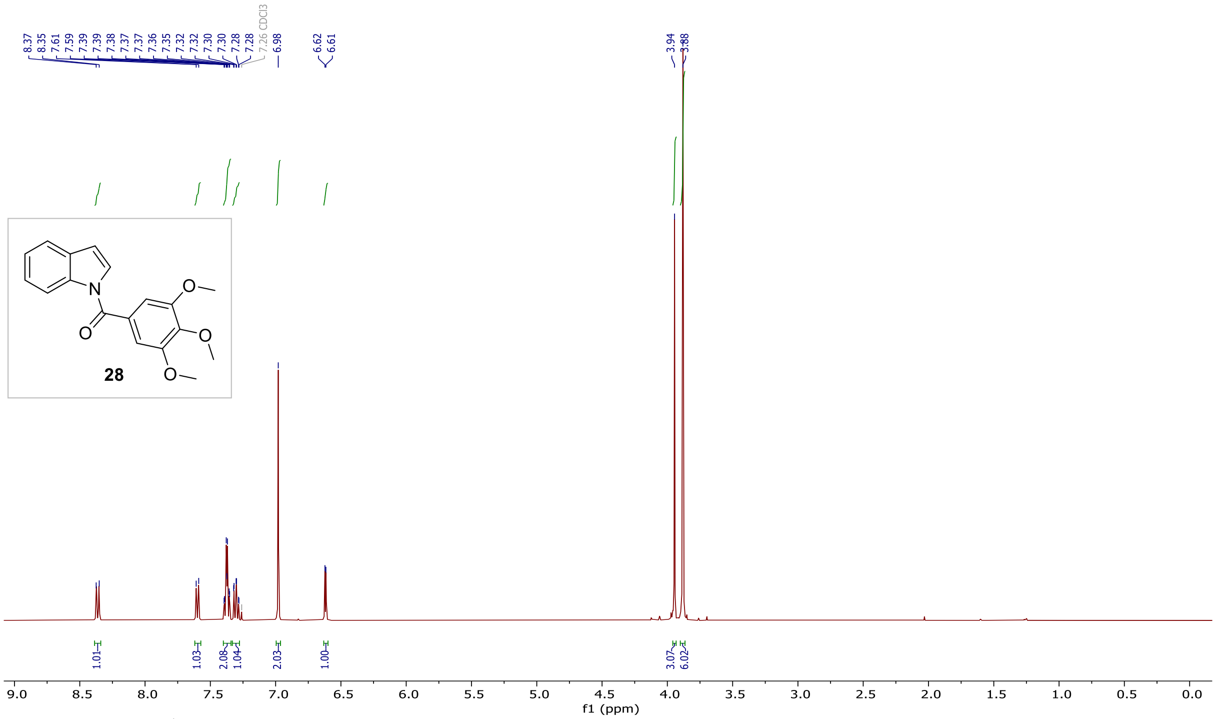

Figure S23. ^{1}H NMR (400 MHz, $CDCl_3$) spectrum of (1H-indol-1-yl)(3,4,5-trimethoxyphenyl)methanone (**28**).

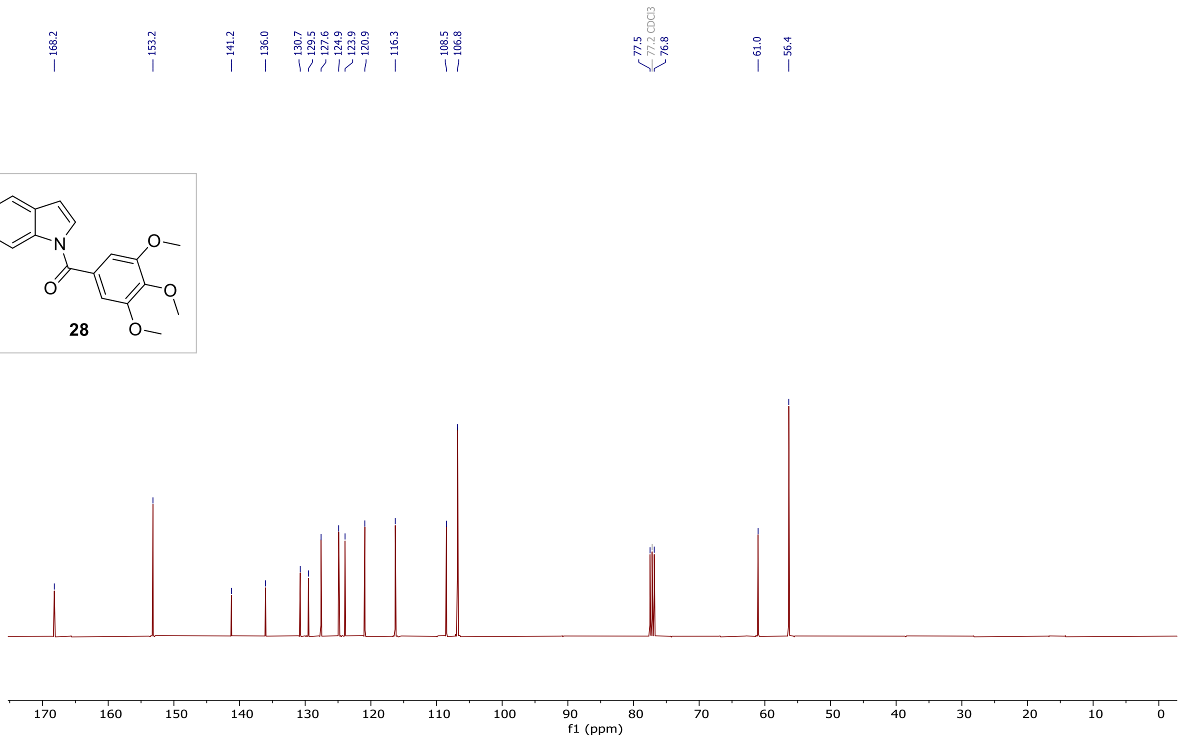

Figure S24. $^{13}C\{^{1}H\}$ NMR (101 MHz, $CDCl_3$) spectrum of (1H-indol-1-yl)(3,4,5-trimethoxyphenyl)methanone (**28**).

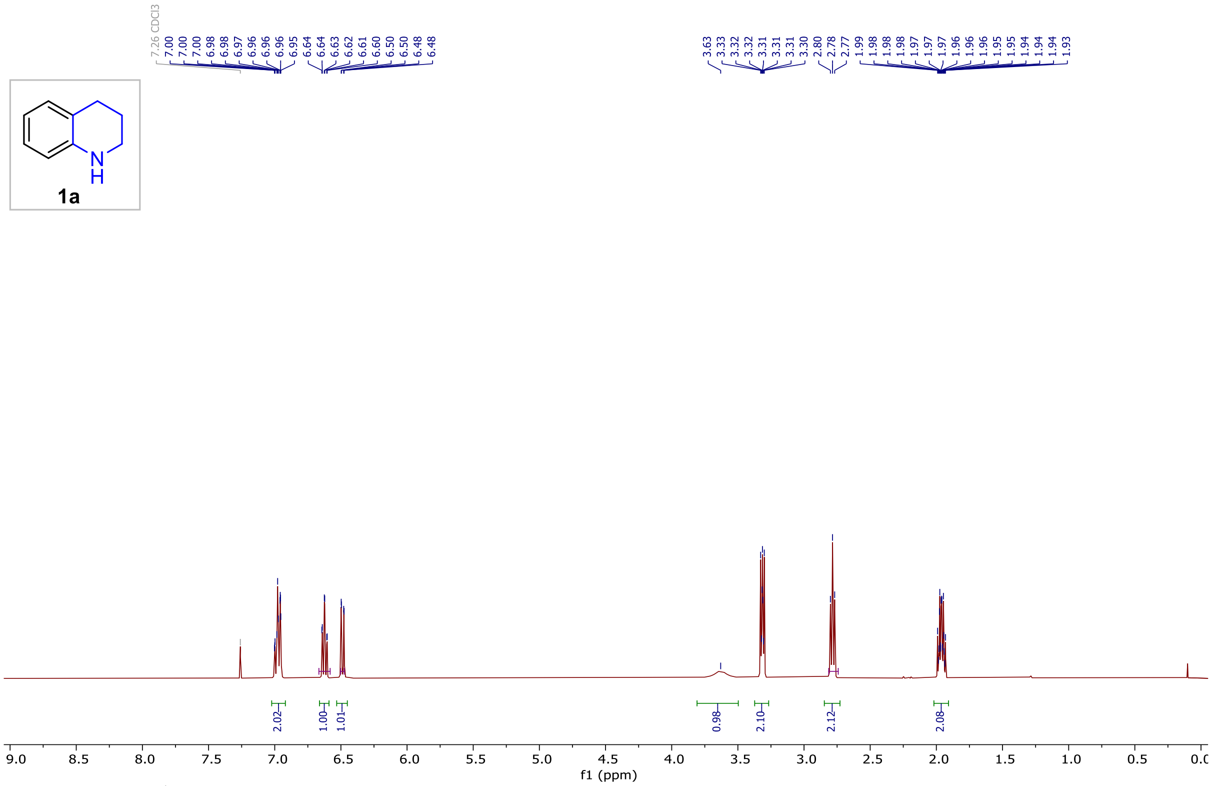

Figure S25.^{1}H NMR (400 MHz, $CDCl_3$) spectrum of 1,2,3,4-tetrahydroquinoline (**1a**).

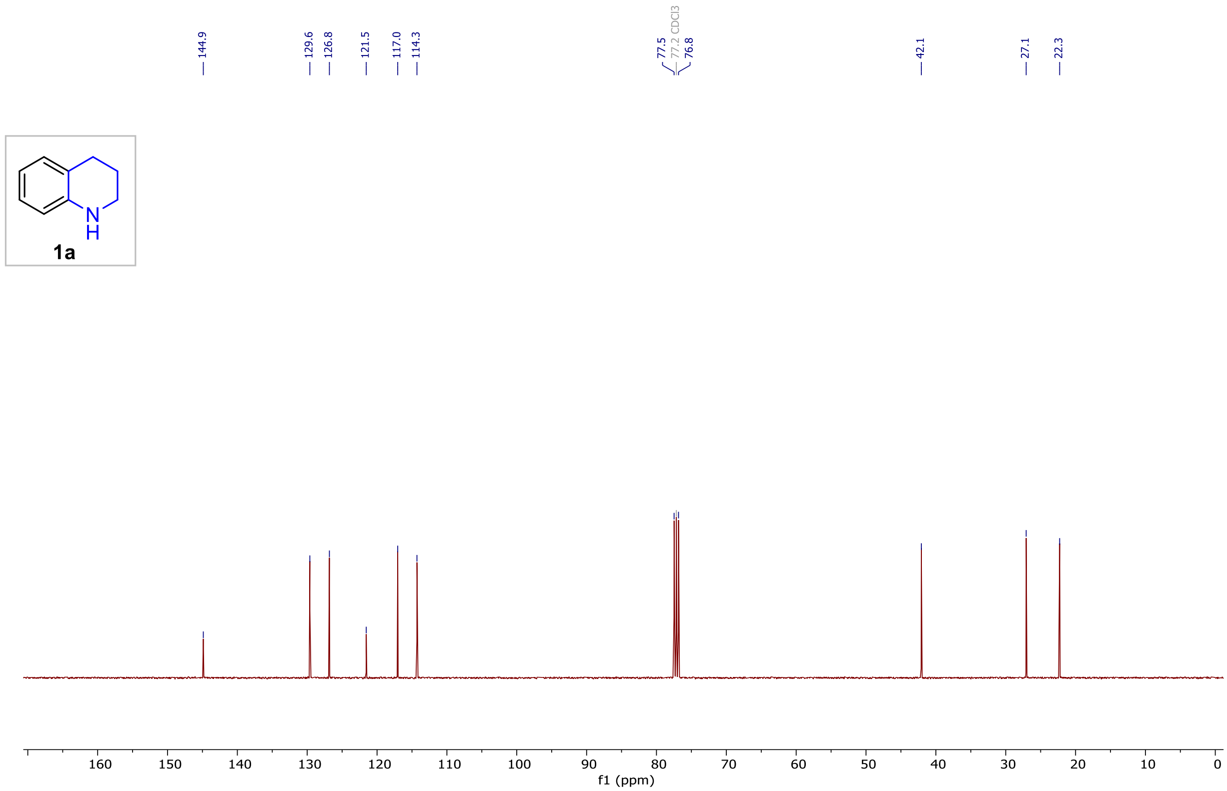

Figure S26. $^{13}C\{^{1}H\}$ NMR (101 MHz, $CDCl_3$) spectrum of 1,2,3,4-tetrahydroquinoline (**1a**).

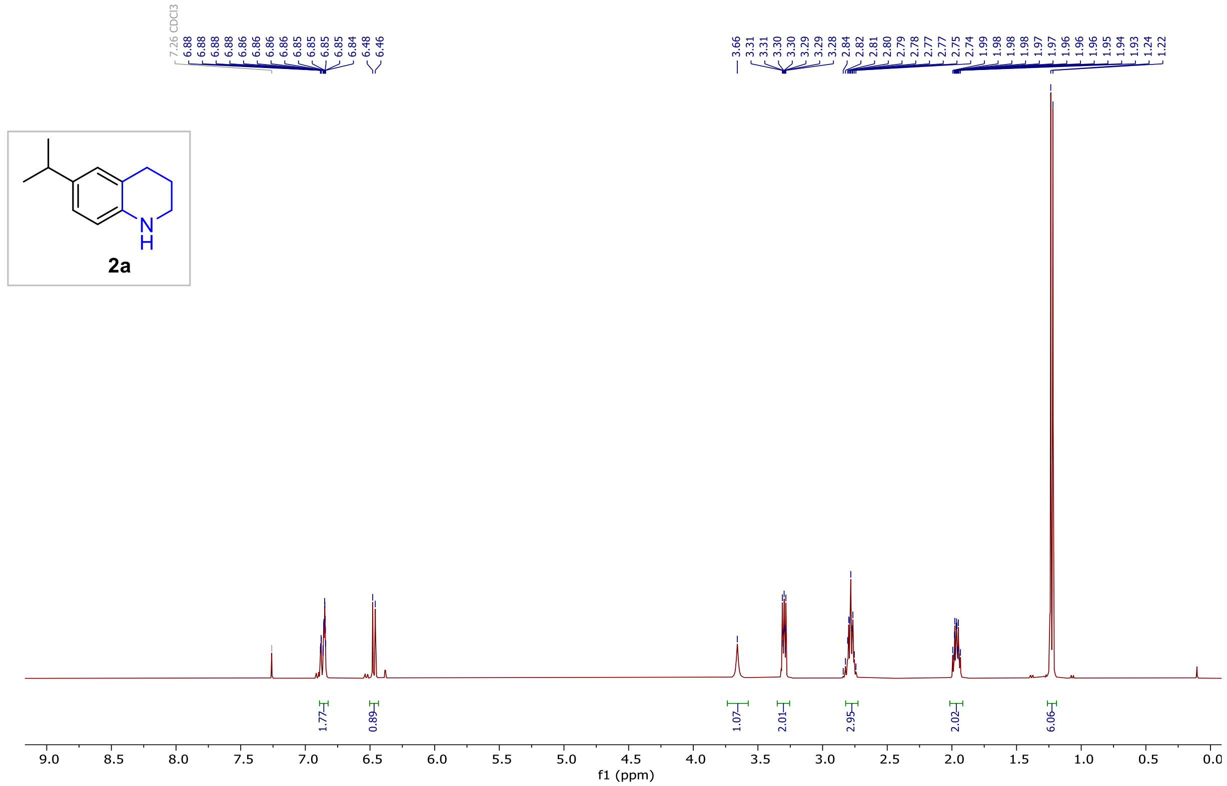

Figure S27. ^{1}H NMR (400 MHz, $CDCl_3$) spectrum of 6-isopropyl-1,2,3,4-tetrahydroquinoline (**2a**).

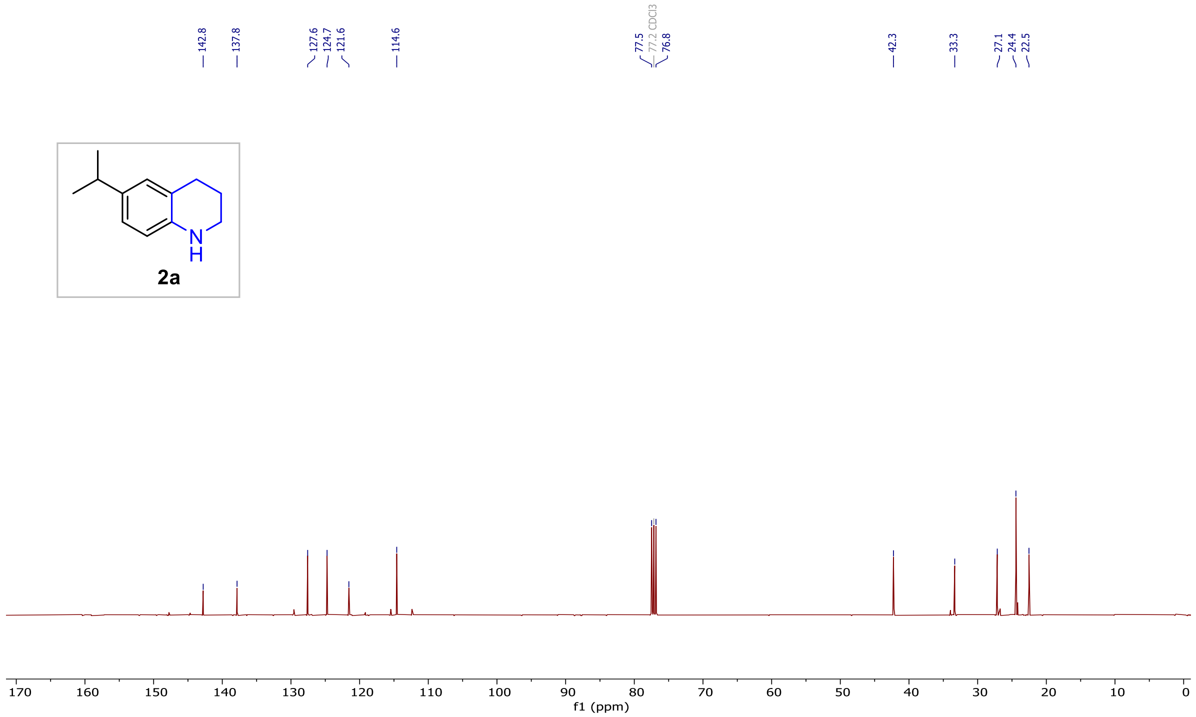

Figure S28. $^{13}C\{^{1}H\}$ NMR (101 MHz, $CDCl_3$) spectrum of 6-isopropyl-1,2,3,4-tetrahydroquinoline (**2a**).

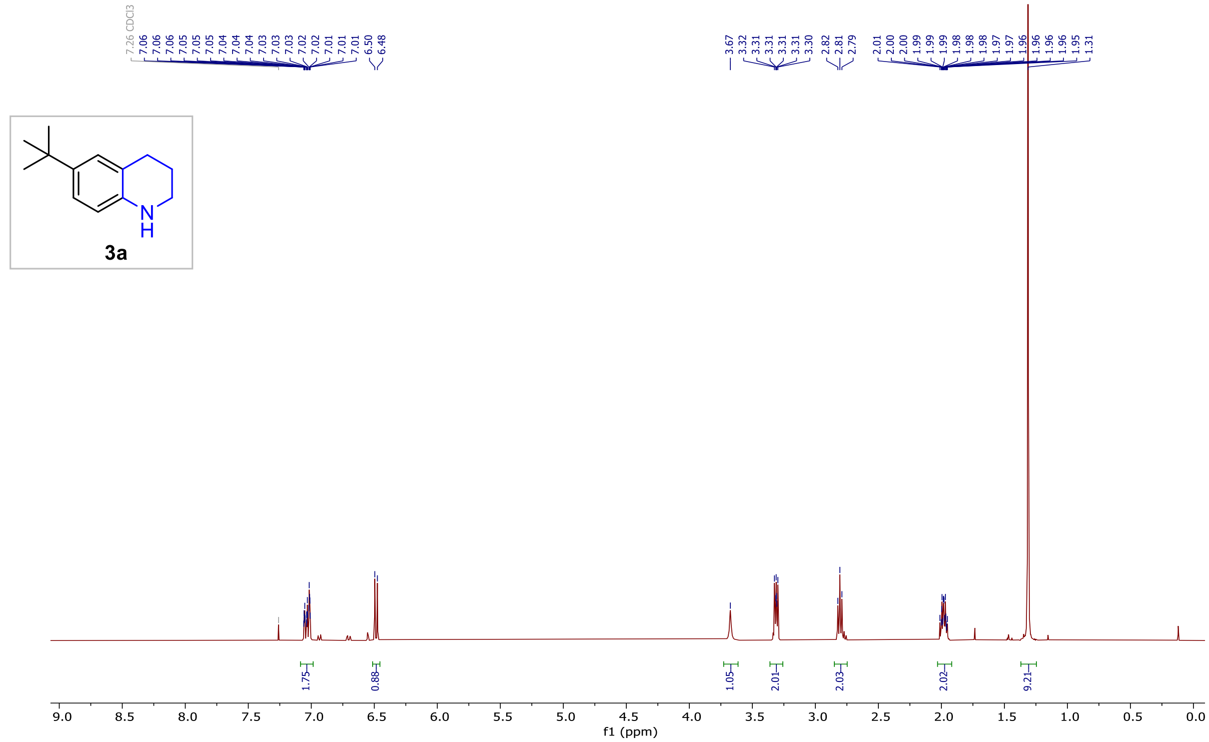

Figure S29. ^{1}H NMR (400 MHz, $CDCl_3$) spectrum of 6-tertbutyl-1,2,3,4-tetrahydroquinoline (**3a**).

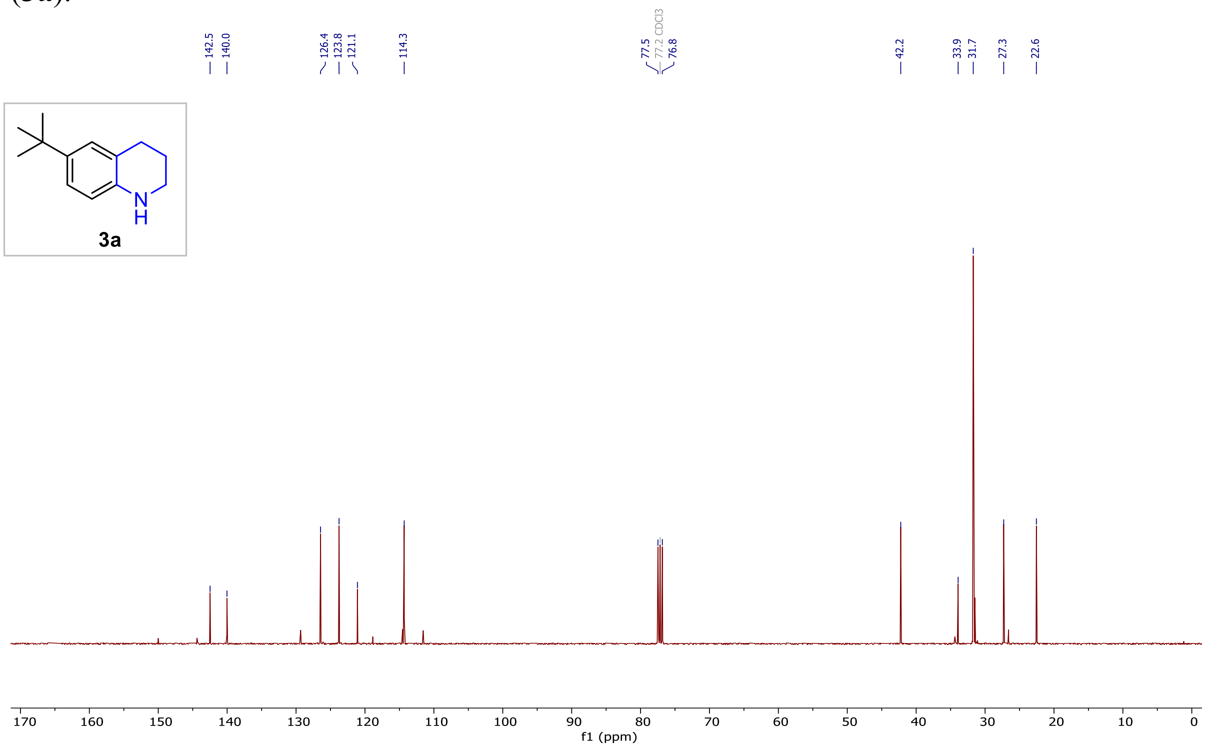

Figure S30. $^{13}C\{^{1}H\}$ NMR (101 MHz, $CDCl_3$) spectrum of 6-tertbutyl-1,2,3,4-tetrahydroquinoline (**3a**).

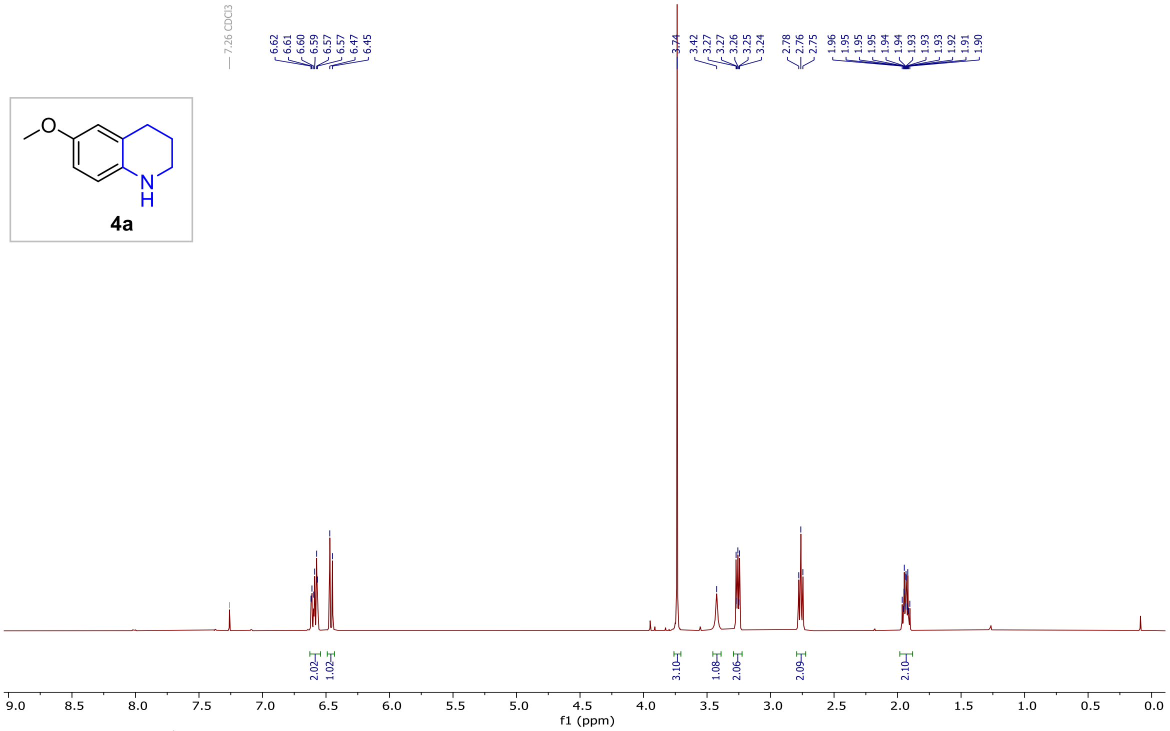

Figure S31. ^{1}H NMR (400 MHz, $CDCl_3$) spectrum of 6-methoxy-1,2,3,4-tetrahydroquinoline (**4a**).

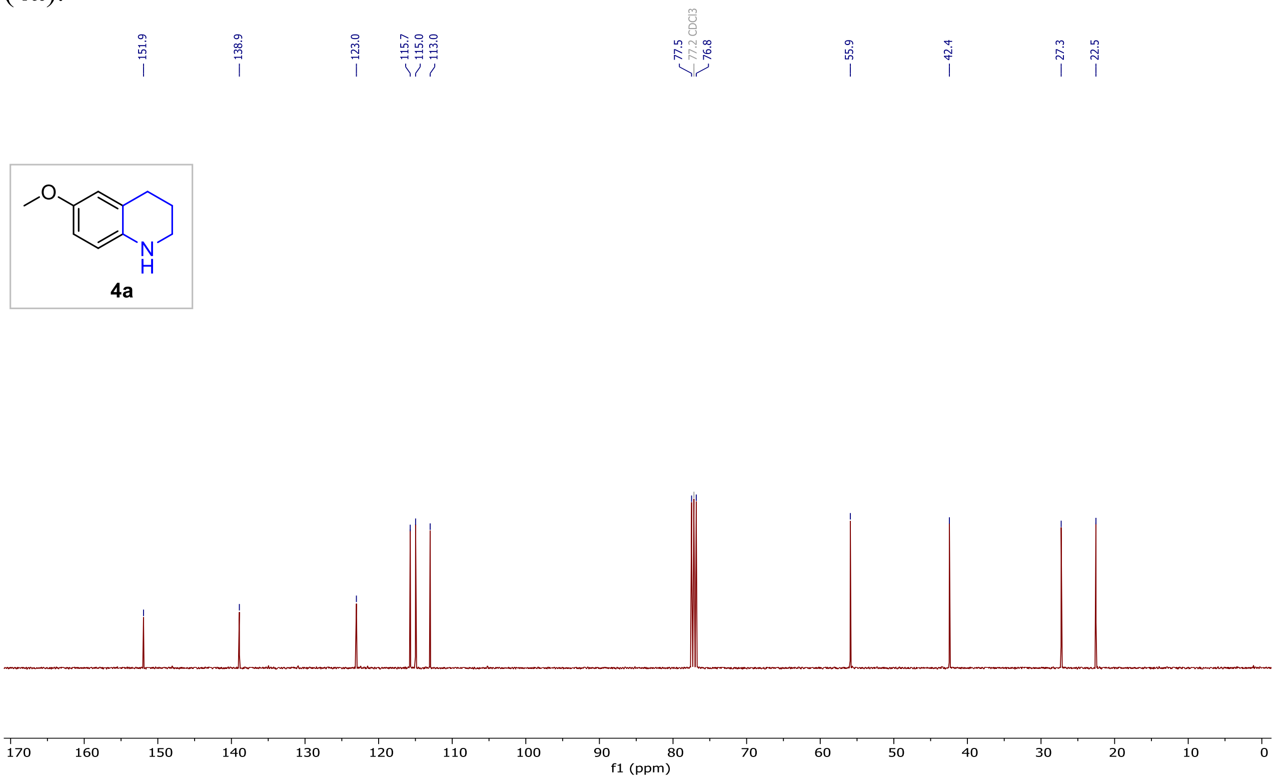

Figure S32. $^{13}C\{^{1}H\}$(101 MHz, $CDCl_3$) NMR spectrum of 6-methoxy-1,2,3,4-tetrahydroquinoline (**4a**).

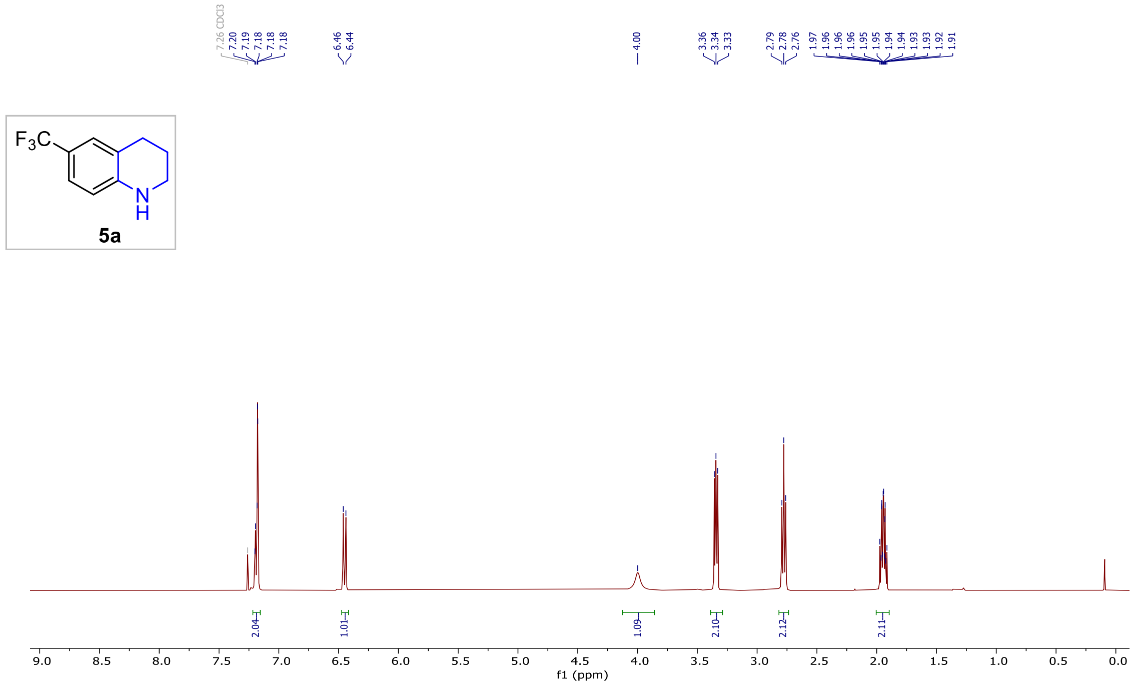

Figure S33. ^{1}H NMR (400 MHz, $CDCl_3$) spectrum of 6-(trifluoromethyl)-1,2,3,4-tetrahydroquinoline (**5a**).

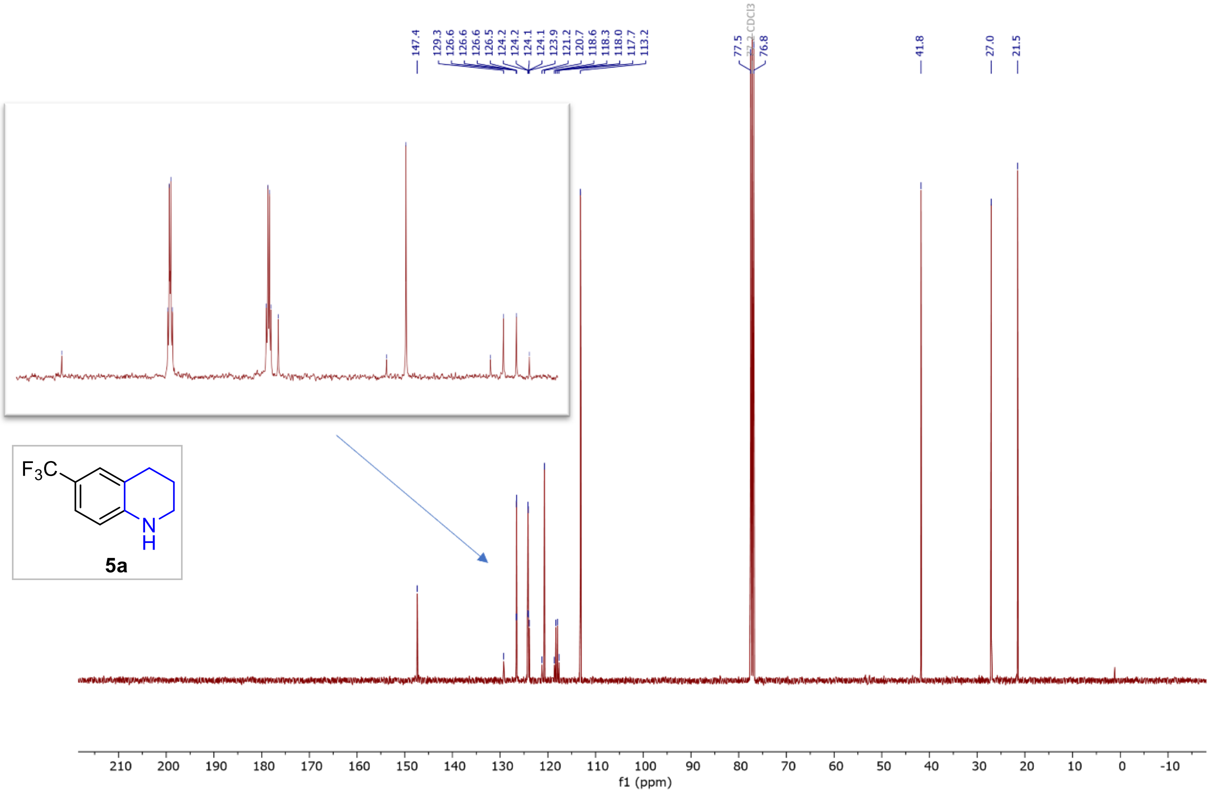

Figure S34. ^{13}C{^{1}H} NMR (101 MHz, $CDCl_3$) spectrum of 6-(trifluoromethyl)-1,2,3,4-tetrahydroquinoline (**5a**).

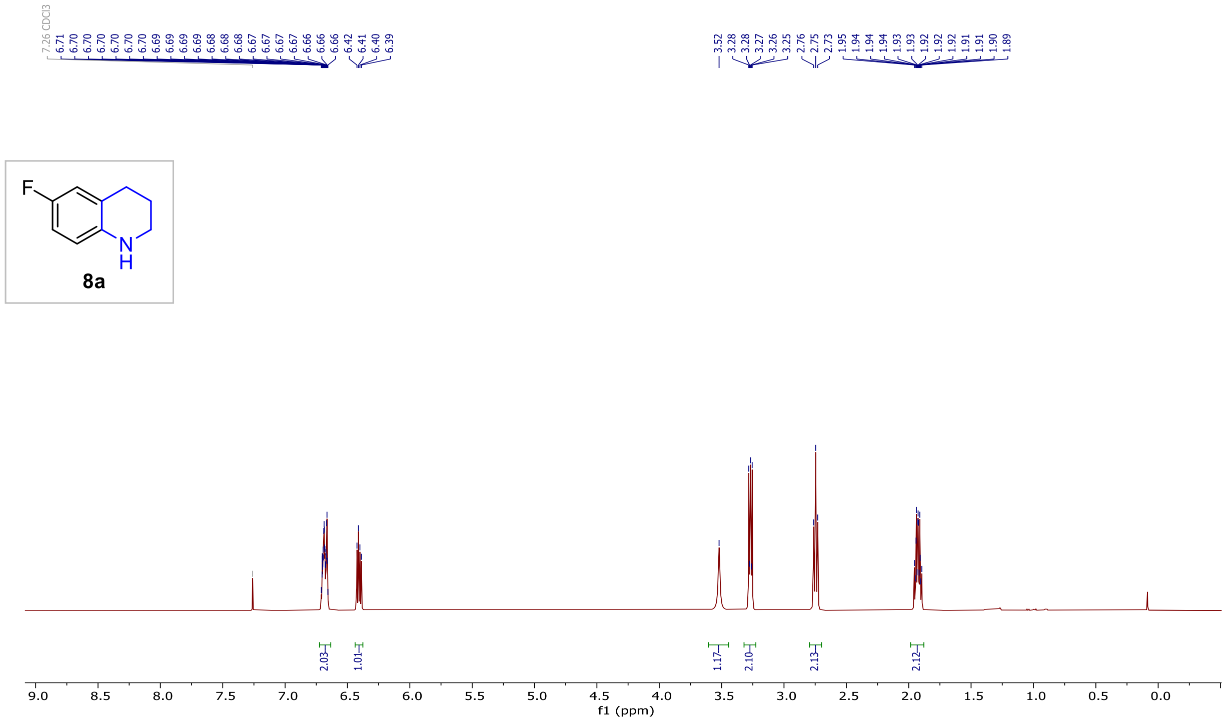

Figure S35. ^{1}H NMR (400 MHz, $CDCl_3$) spectrum of 6-fluoro-1,2,3,4-tetrahydroquinoline (**8a**).

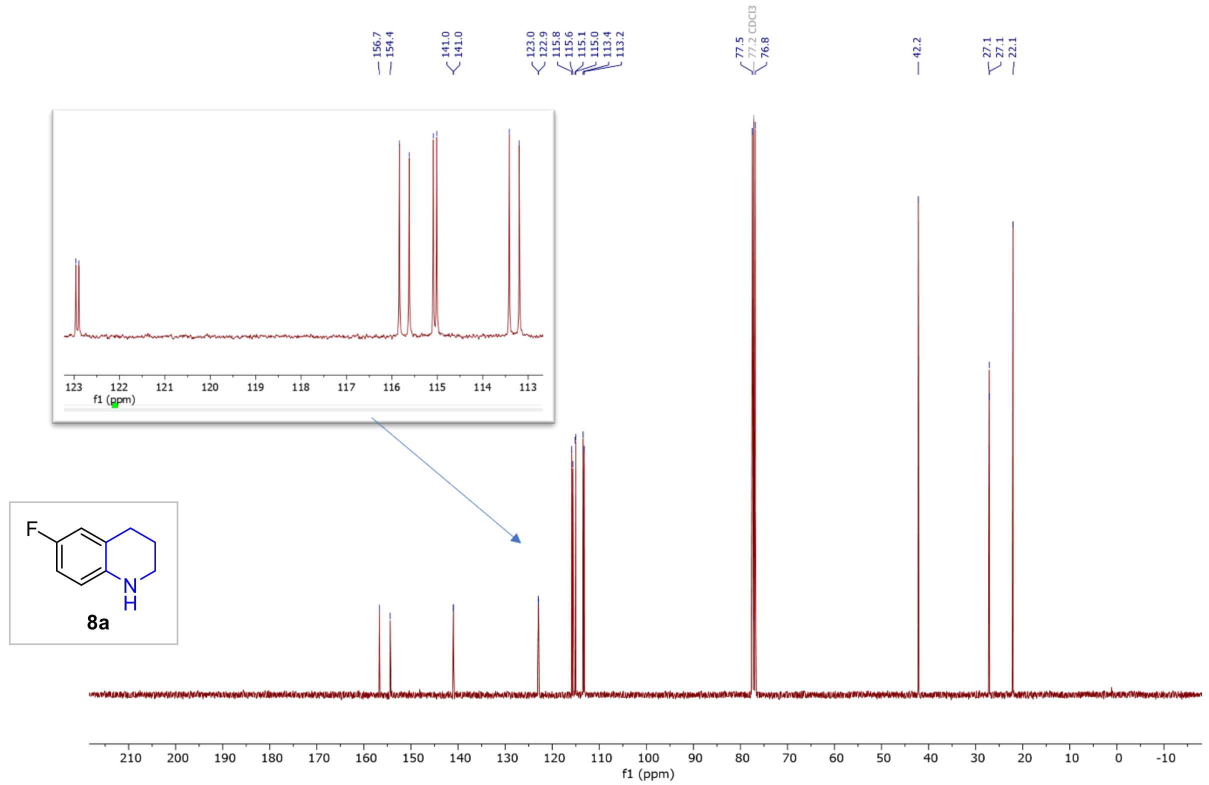

Figure S36. $^{13}C\{^{1}H\}$(101 MHz, $CDCl_3$) NMR spectrum of 6-fluoro-1,2,3,4-tetrahydroquinoline (**8a**).

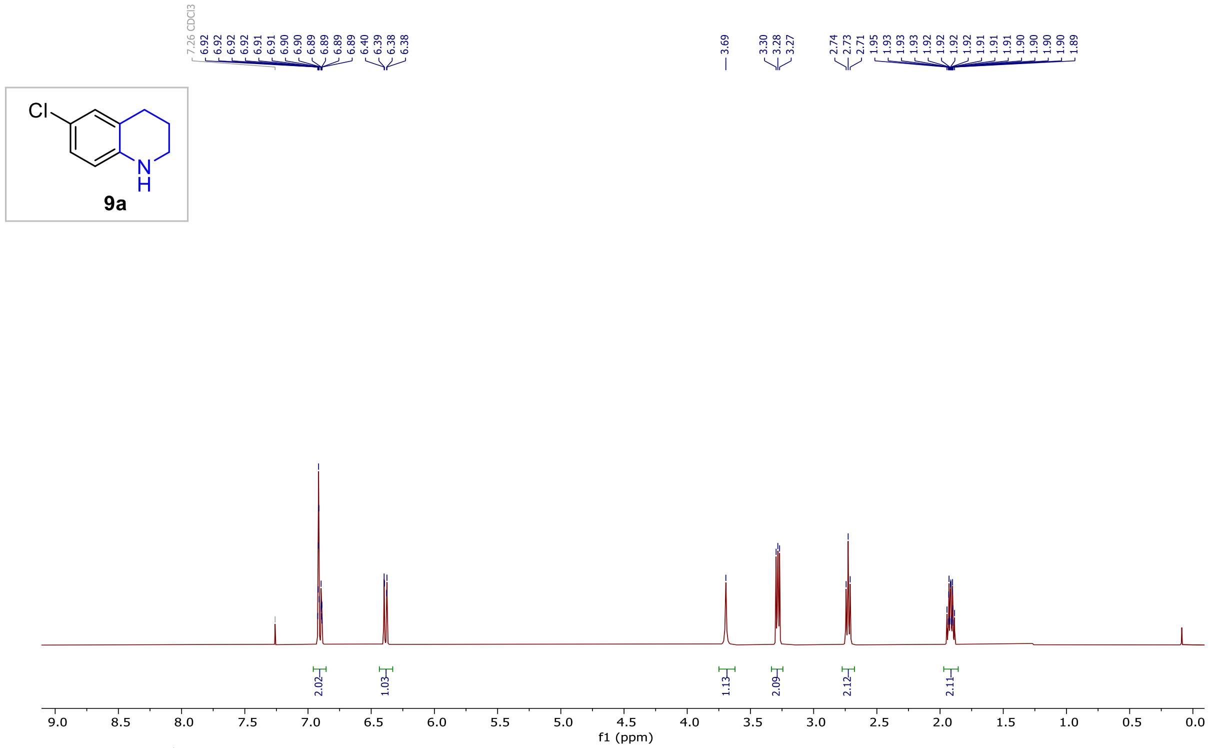

Figure S37. ^{1}H NMR (400 MHz, $CDCl_3$) spectrum of 6-chloro-1,2,3,4-tetrahydroquinoline (**9a**).

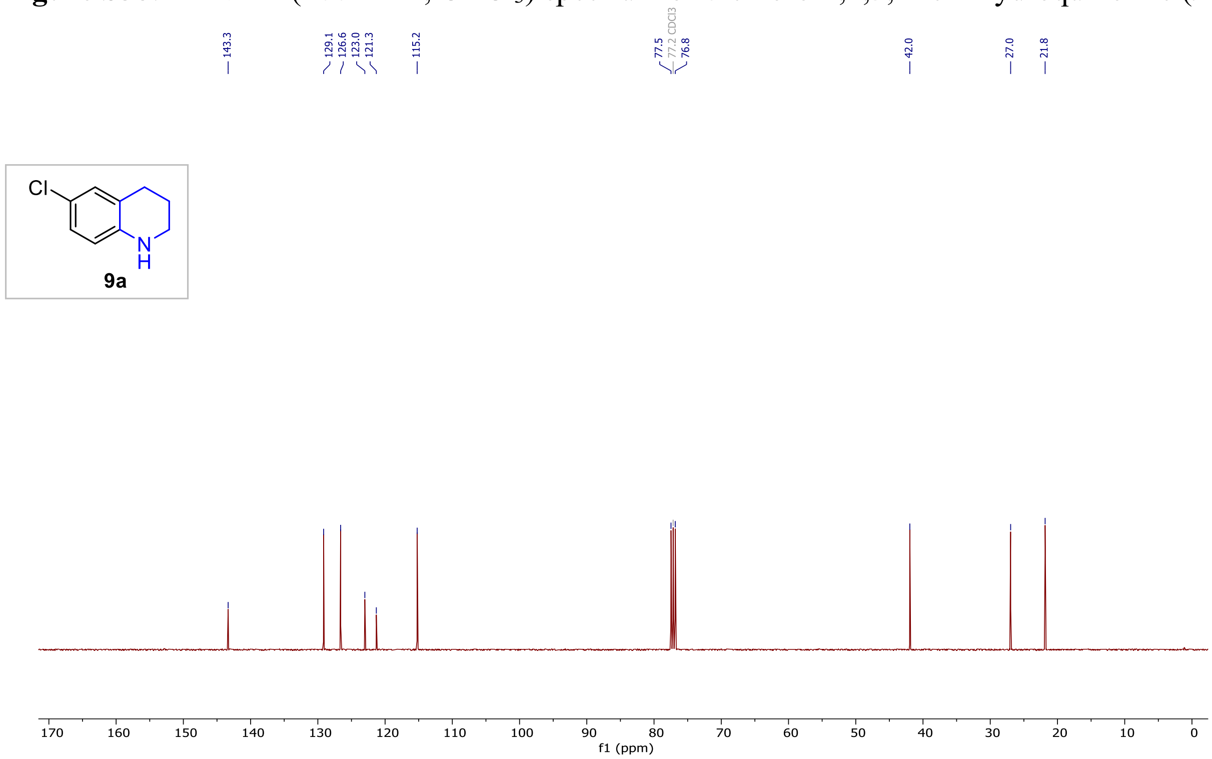

Figure S38. $^{13}C\{^{1}H\}$ (101 MHz, $CDCl_3$) NMR spectrum of 6-chloro-1,2,3,4-tetrahydroquinoline (**9a**).

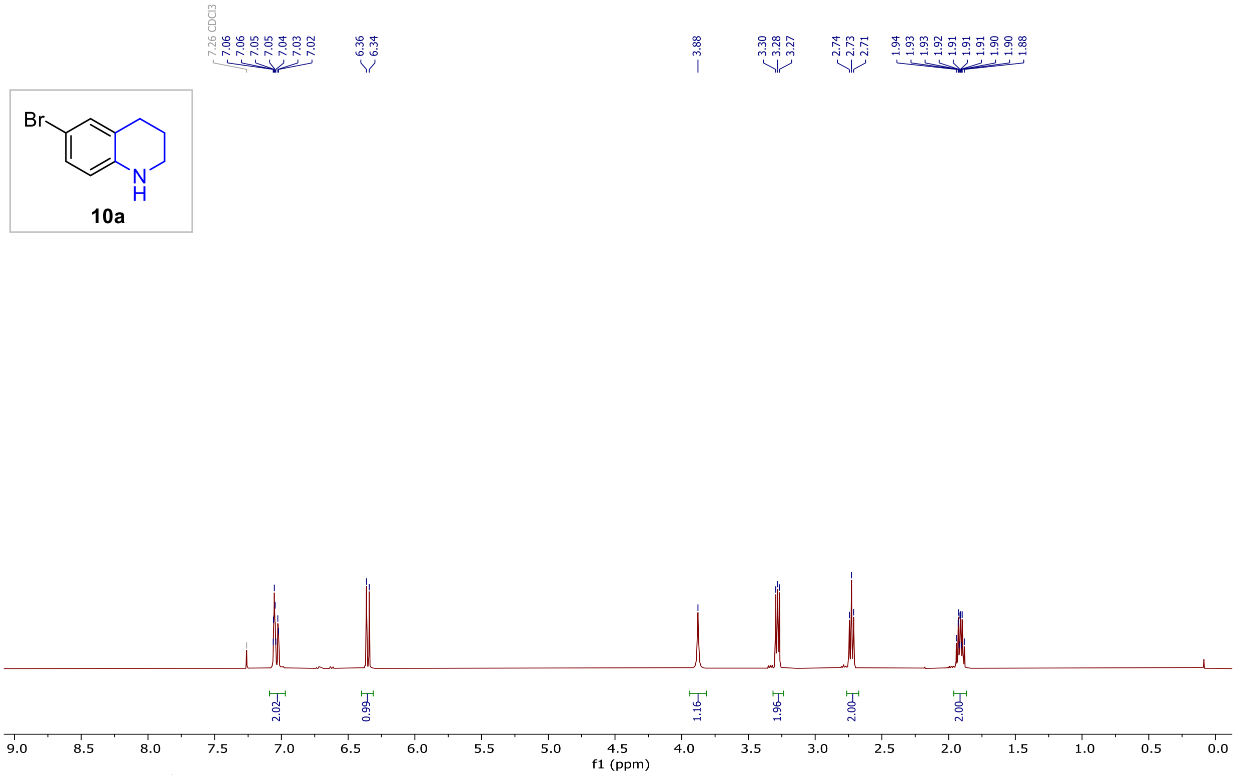

Figure S39. ^{1}H NMR (400 MHz, $CDCl_3$) spectrum of 6-bromo-1,2,3,4-tetrahydroquinoline (**10a**).

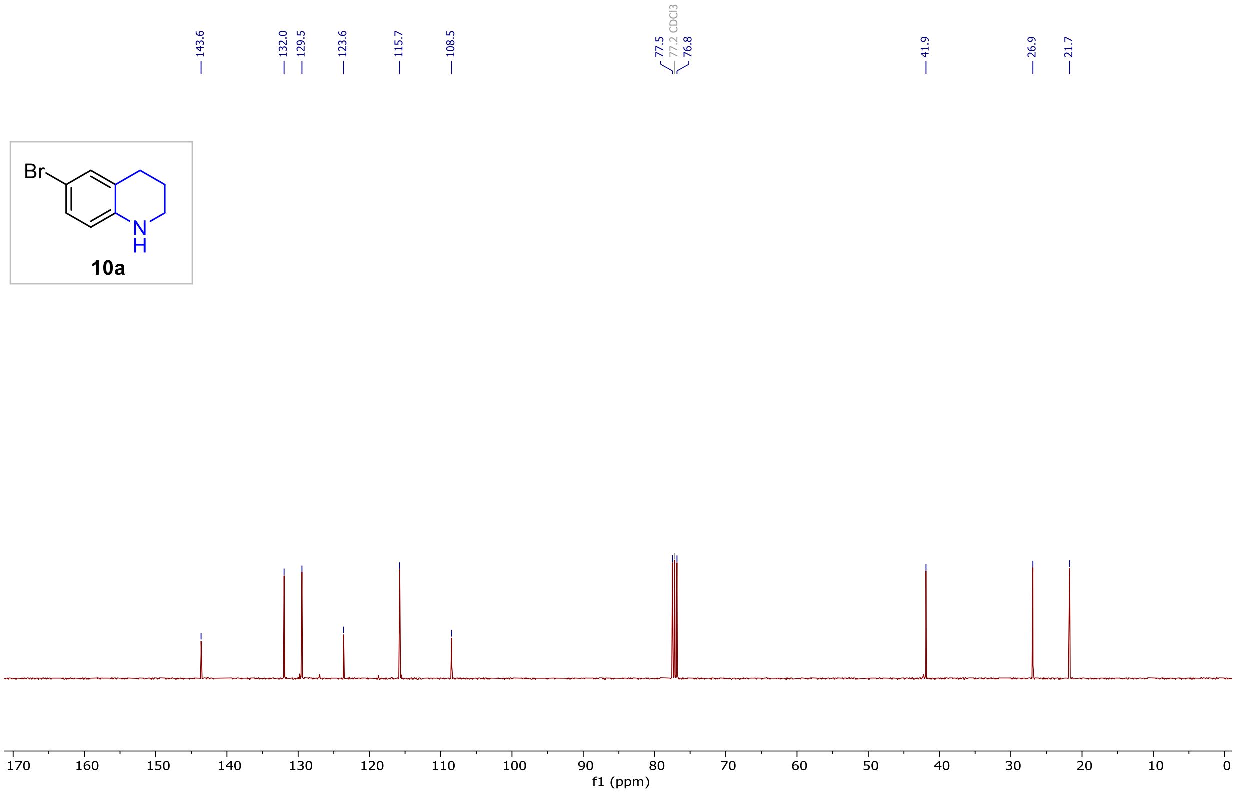

Figure S40. $^{13}C\{^{1}H\}$ NMR (101 MHz, $CDCl_3$) spectrum of 6-bromo-1,2,3,4-tetrahydroquinoline (**10a**).

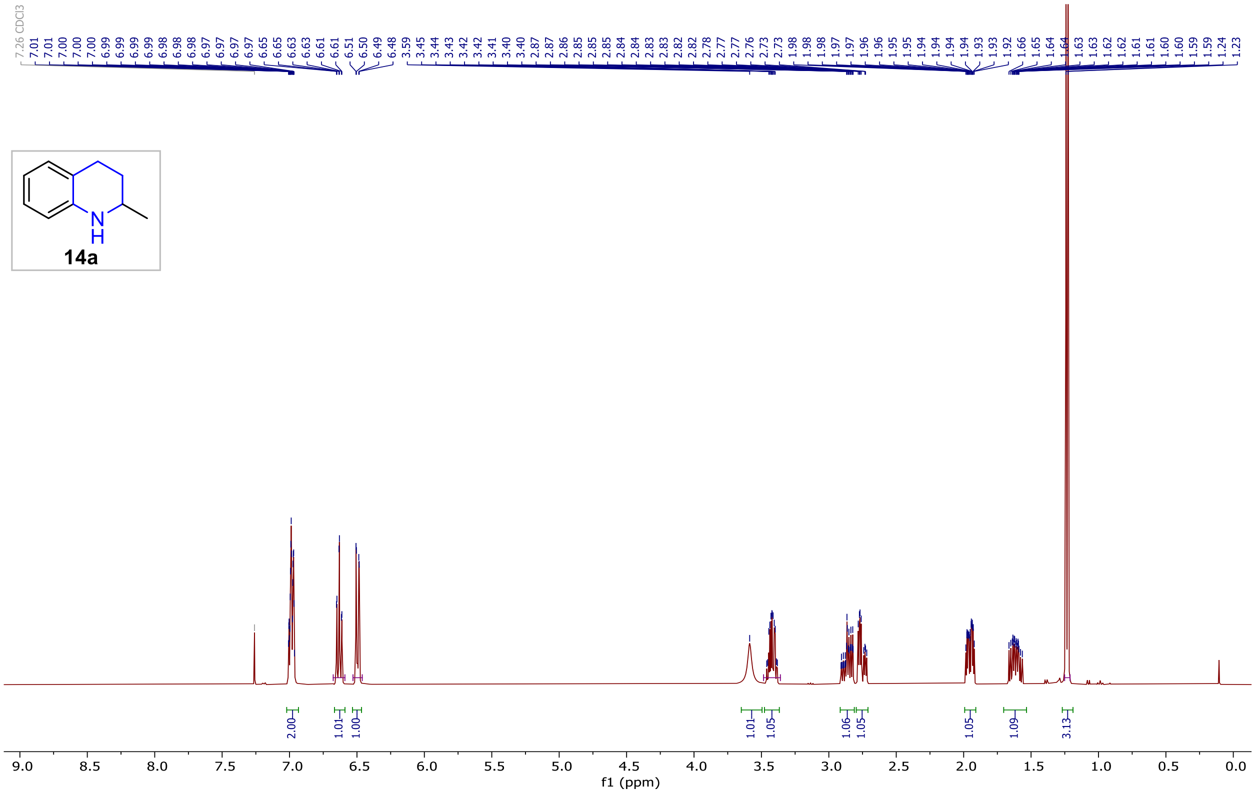

Figure S41. ^{1}H NMR (400 MHz, $CDCl_3$) spectrum of 2-methyl-1,2,3,4-tetrahydroquinoline (**14a**).

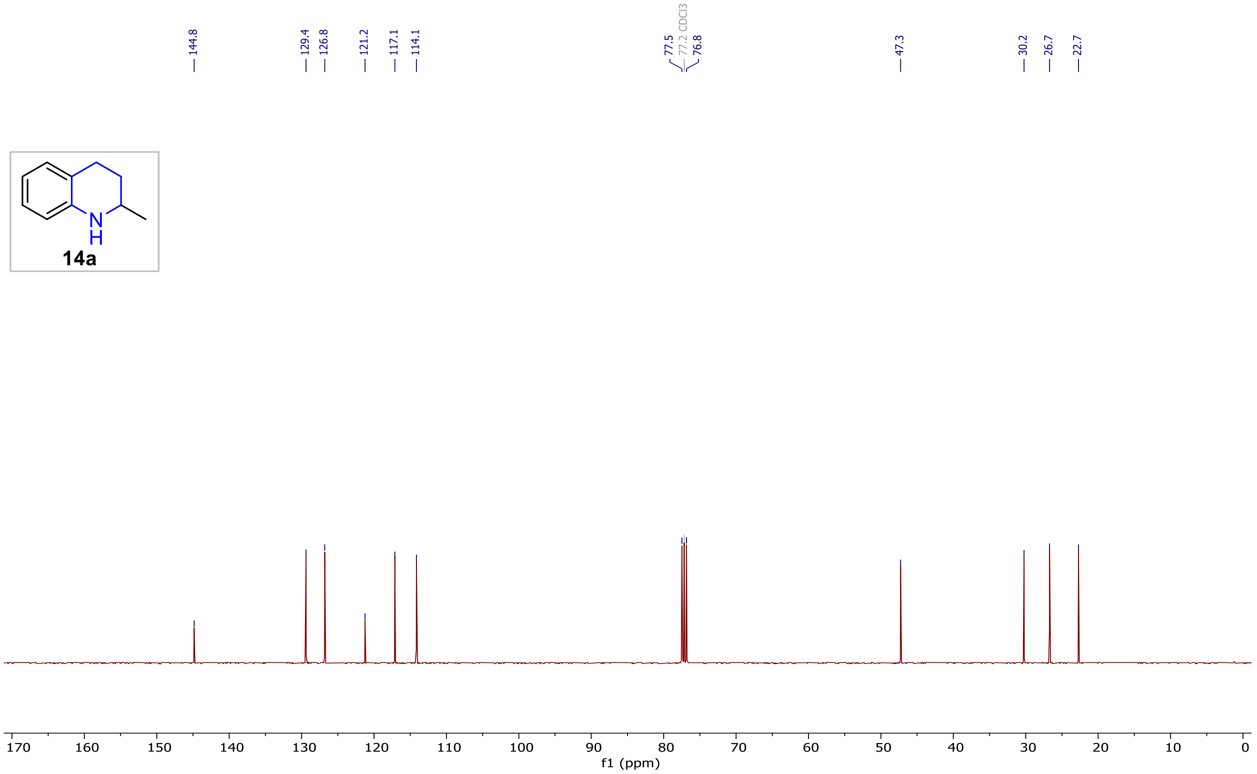

Figure S42. $^{13}C\{^1H\}$ NMR (101 MHz, $CDCl_3$) spectrum of 2-methyl-1,2,3,4-tetrahydroquinoline (**14a**).

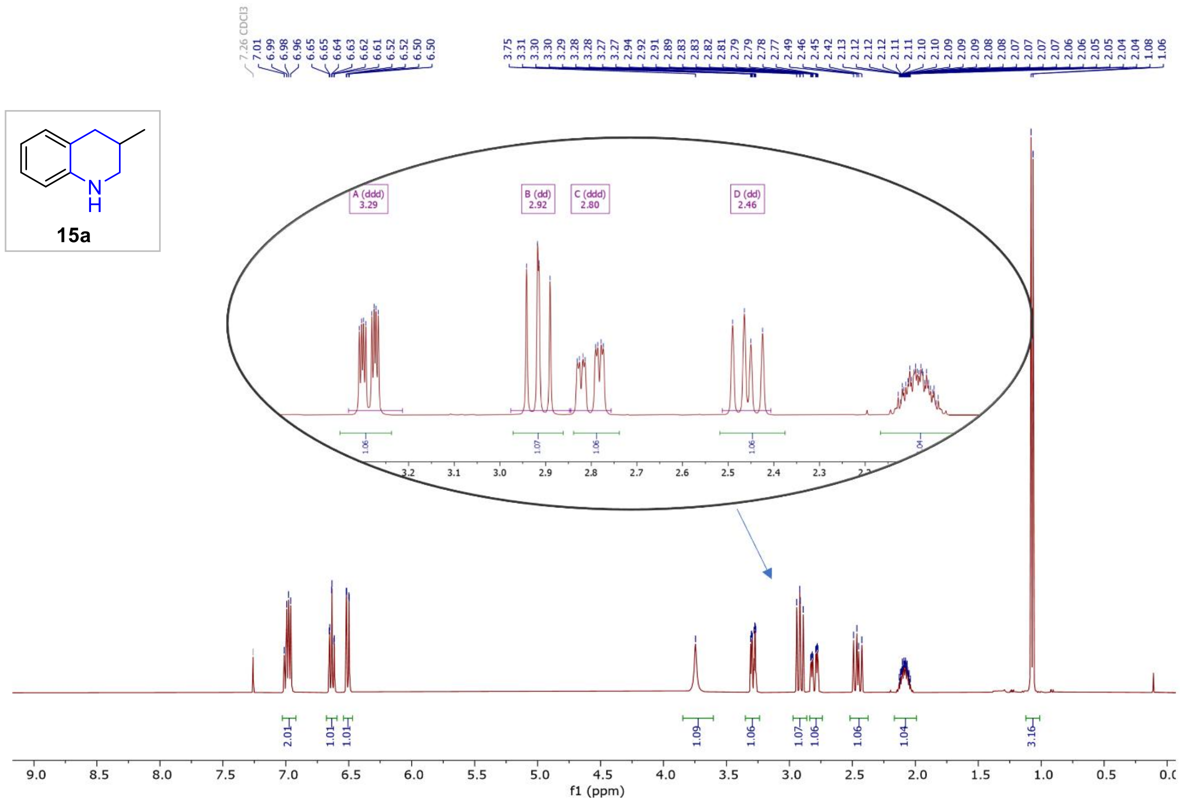

Figure S43. ^{1}H NMR (400 MHz, $CDCl_3$) spectrum of 3-methyl-1,2,3,4-tetrahydroquinoline (**15a**).

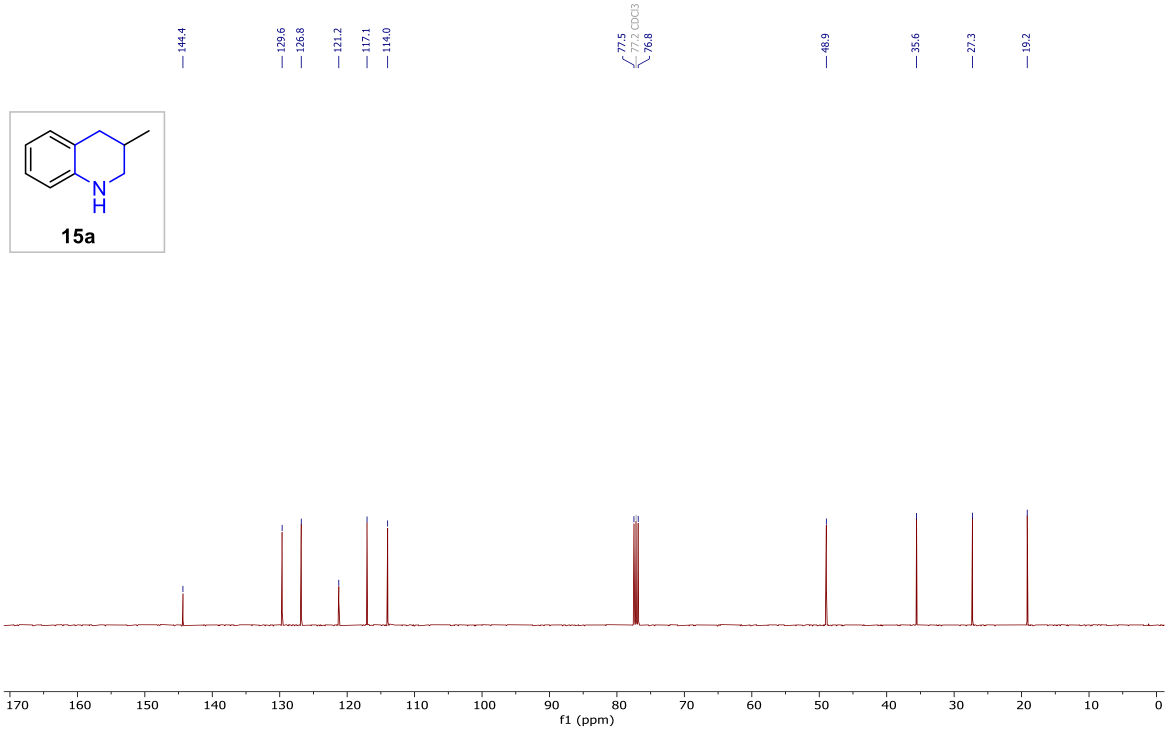

Figure S44. $^{13}C\{^{1}H\}$ NMR (101 MHz, $CDCl_3$) spectrum 3-methyl-1,2,3,4-tetrahydroquinoline (**15a**).

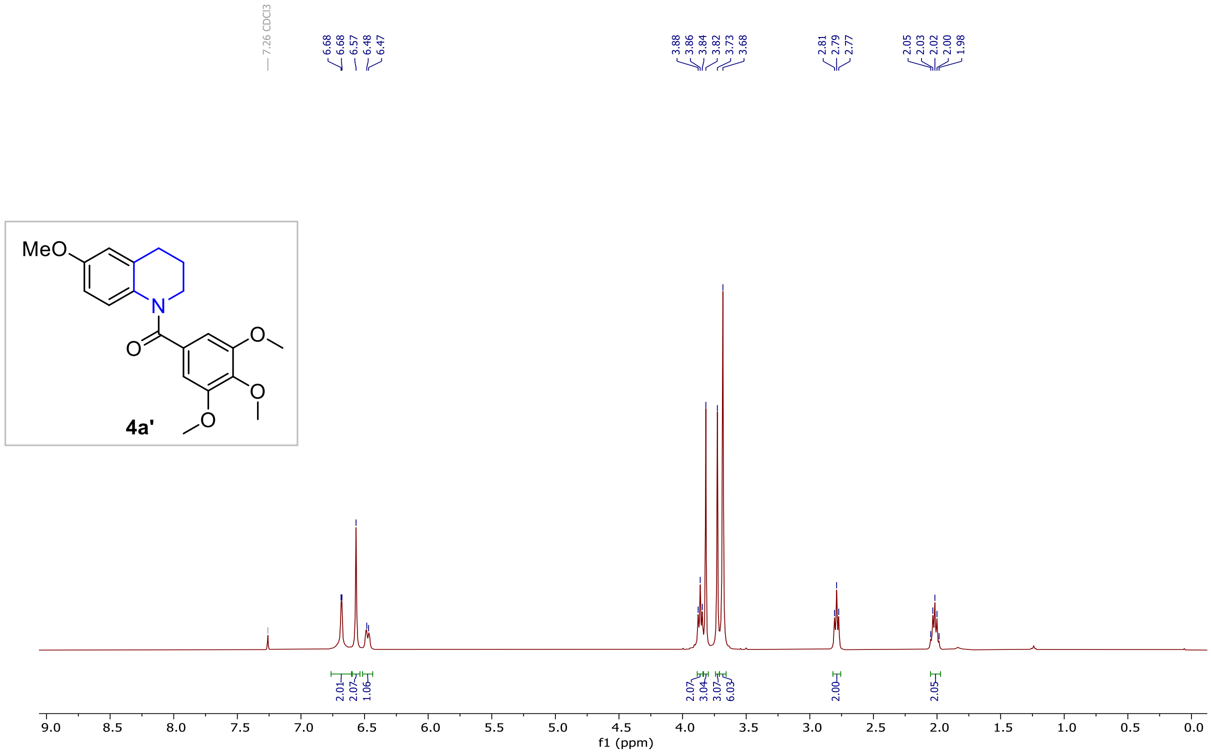

Figure S45. ^{1}H NMR (400 MHz, $CDCl_3$) spectrum of (6-methoxy-3,4-dihydroquinolin-1(2H)-yl)(3,4,5-trimethoxyphenyl)methanone(**4a'**).

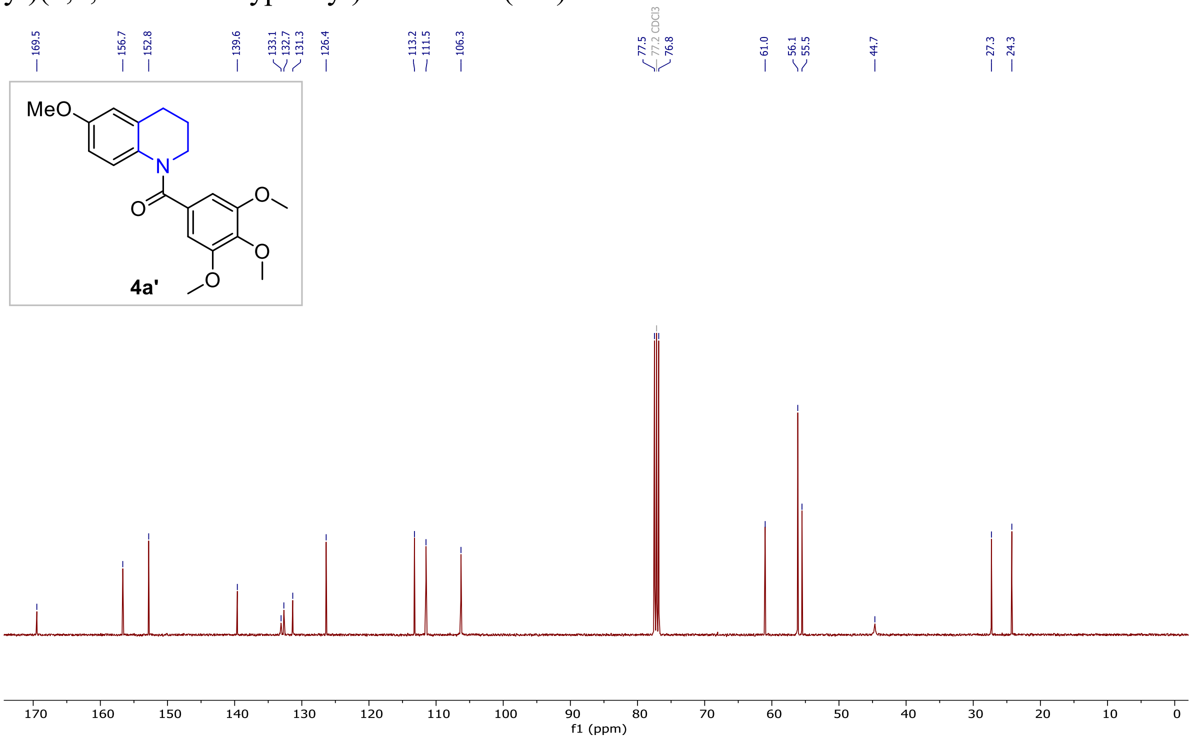

Figure S46. $^{13}C\{^{1}H\}$NMR (101 MHz, $CDCl_3$) spectrum of (6-methoxy-3,4-dihydroquinolin-1(2H)-yl)(3,4,5-trimethoxyphenyl)methanone (**4a'**).

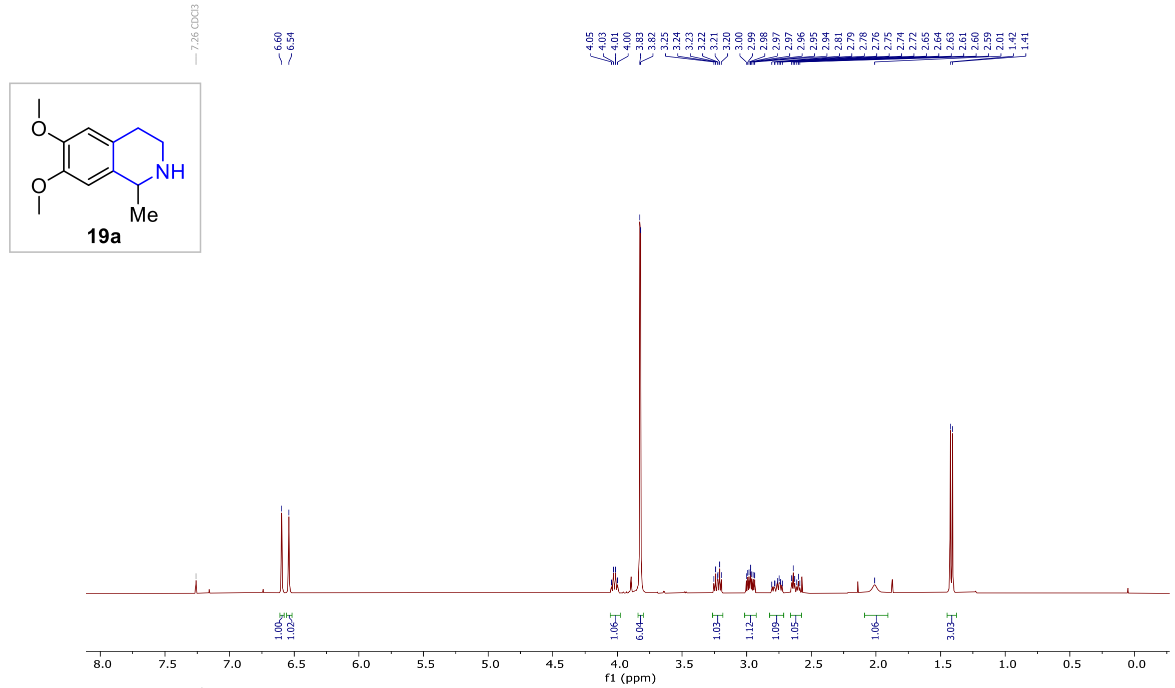

Figure S47. ^{1}H NMR (400 MHz, $CDCl_3$) spectrum of 6,7-dimethoxy-1-methyl-1,2,3,4-tetrahydroisoquinoline (**19a**).

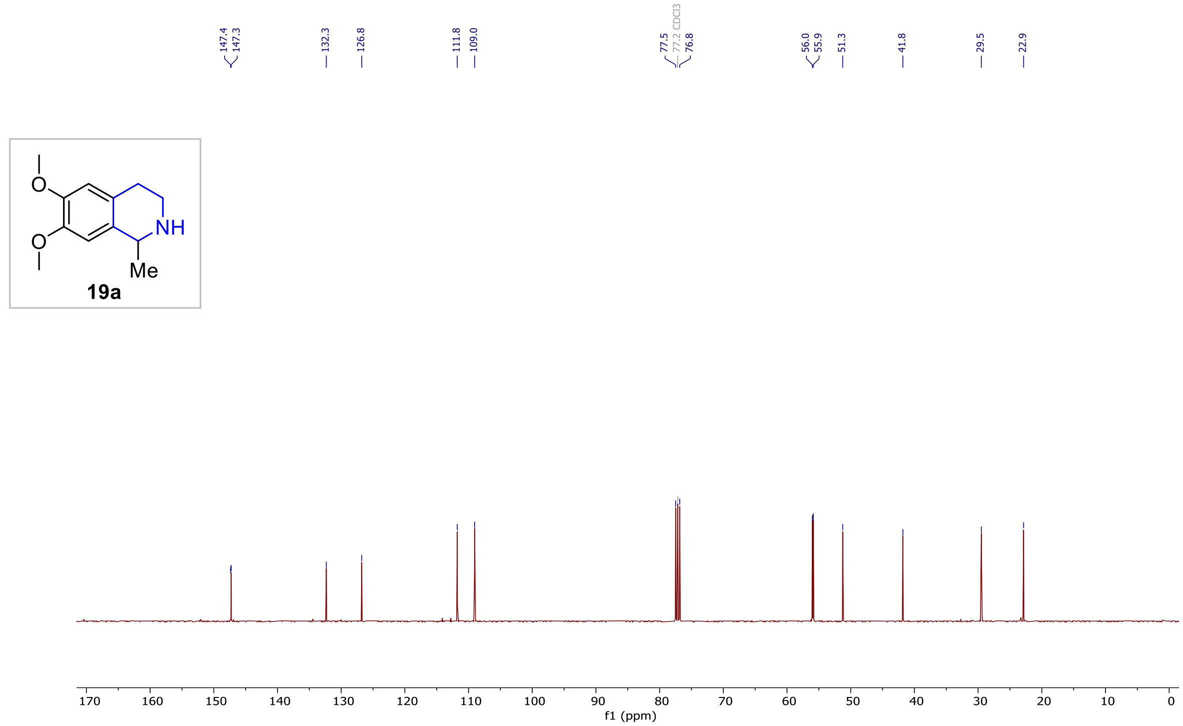

Figure S48. $^{13}C\{^{1}H\}$ NMR (101 MHz, $CDCl_3$) spectrum of 6,7-dimethoxy-1-methyl-1,2,3,4-tetrahydroisoquinoline (**19a**).

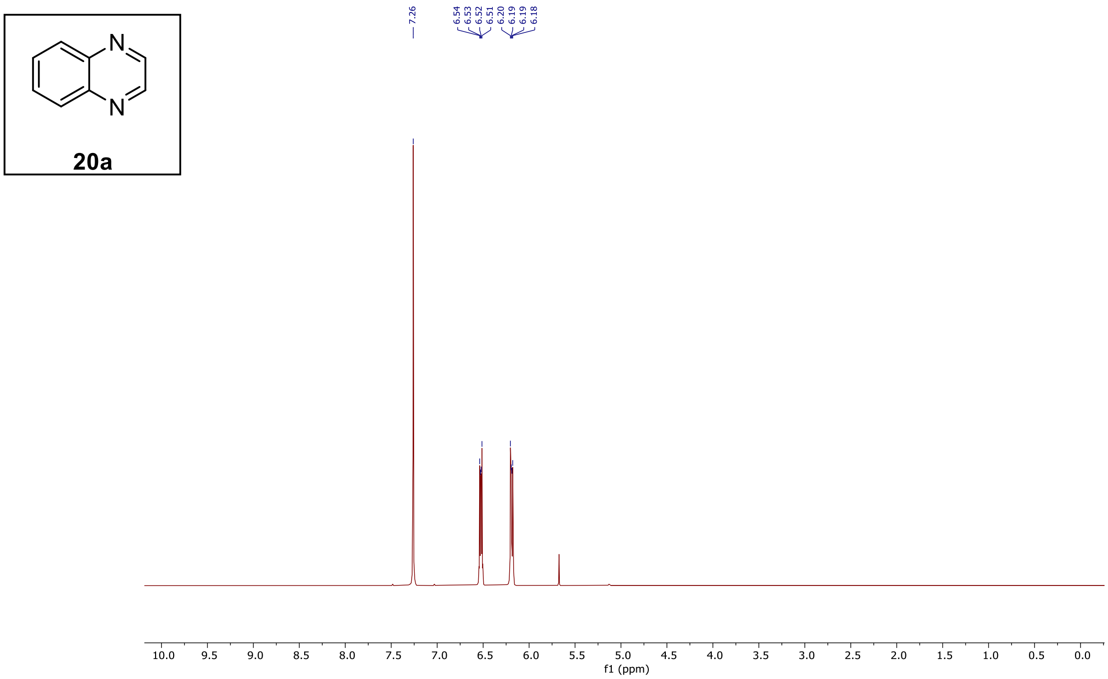

Figure S49. ^{1}H NMR (400 MHz, $CDCl_3$) spectrum of quinoxaline (**20a**).

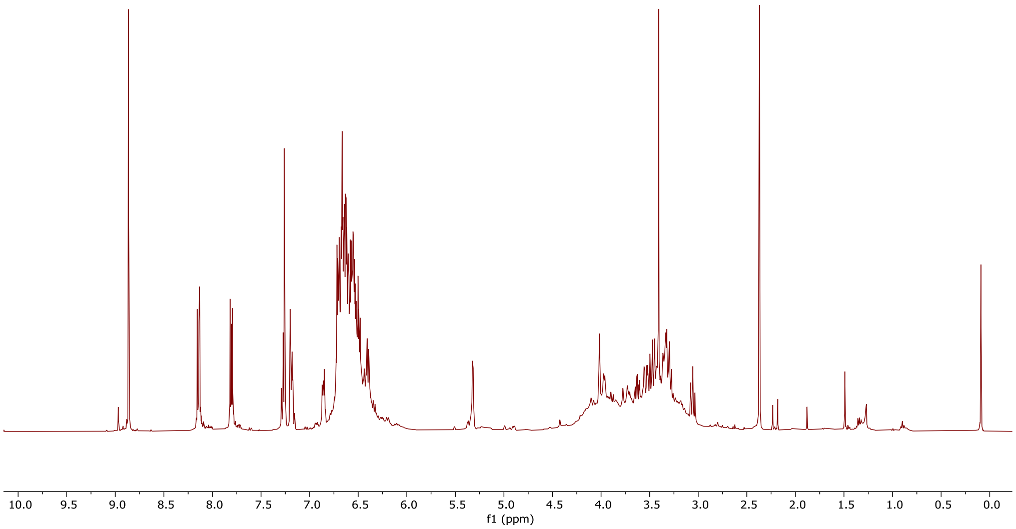

Figure S50: ^{1}H NMR (400 MHz, $CDCl_3$) spectrum of the crude reaction mixture of the hydrogenation of quinoxaline in toluene under standard conditions.

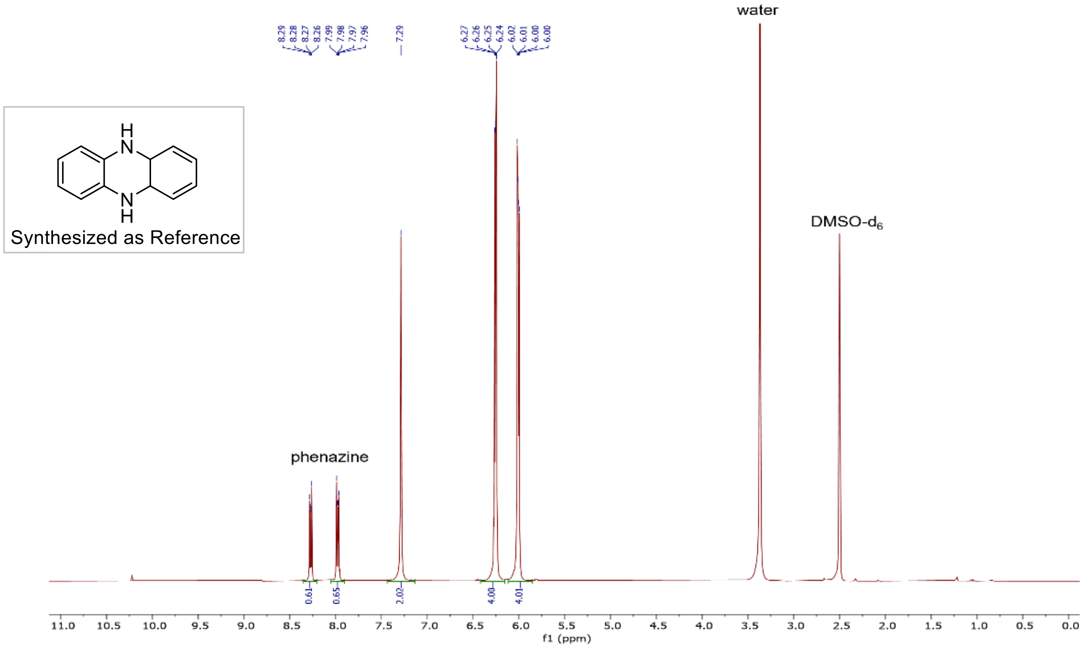

Figure S51. ^{1}H NMR (400 MHz, DMSO-d$_6$) spectrum of 5,10-dihydrophenazine (**21a**) synthesized as reference following the literature report [28-29].

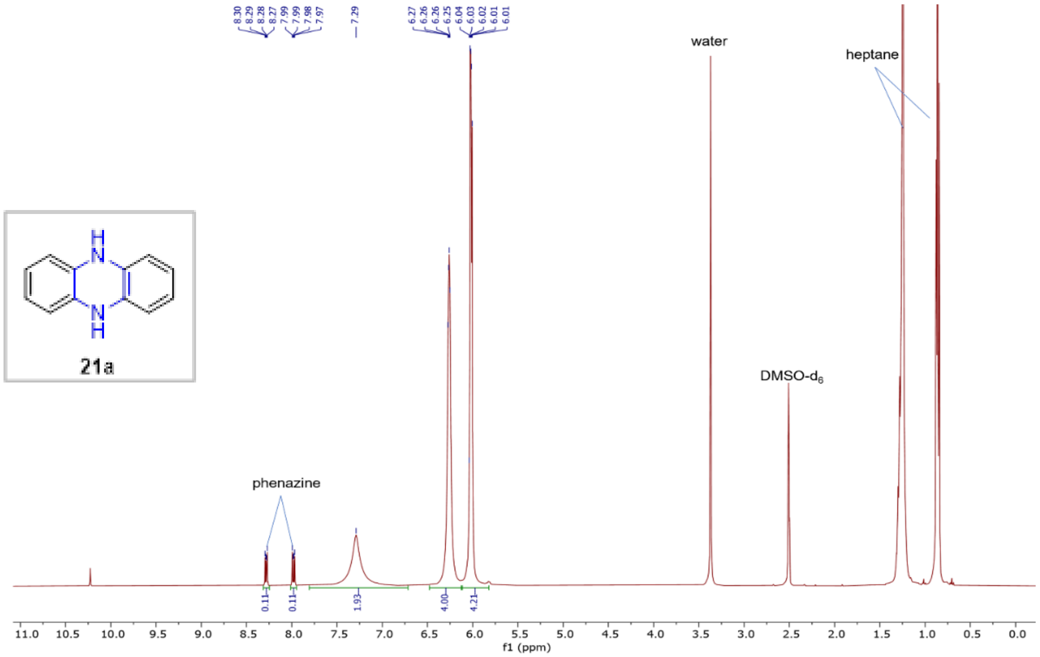

Figure S52. ^{1}H NMR (400 MHz, acetone-d$_6$) spectrum of the crude reaction mixture of the hydrogenation of phenazine (**21a**) under standard conditions. N.B. This product tends to decompose in some organic solvents (e.g. acetone). Therefore, the yield was determined from the corresponding ^{1}H-NMR spectrum in DMSO-d$_6$ in which the product is stable.

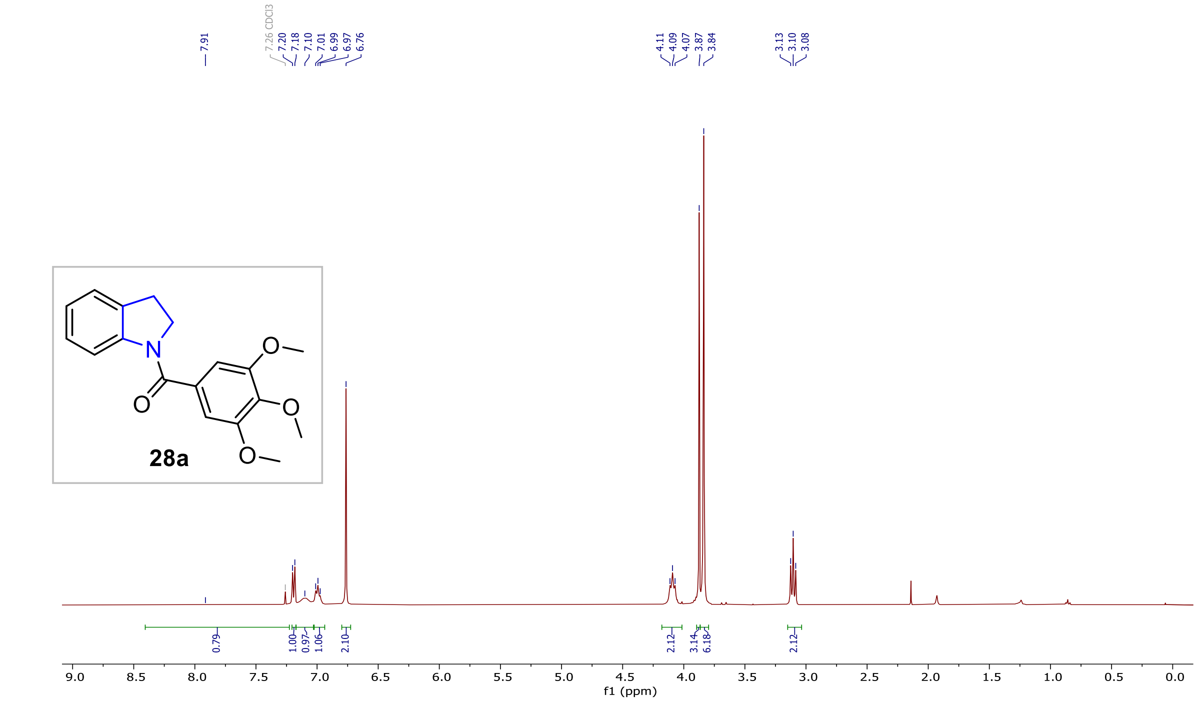

Figure S53. ^{1}H NMR (400 MHz, $CDCl_3$) spectrum of indolin-1-yl(3,4,5-trimethoxyphenyl)methanone (**28a**).

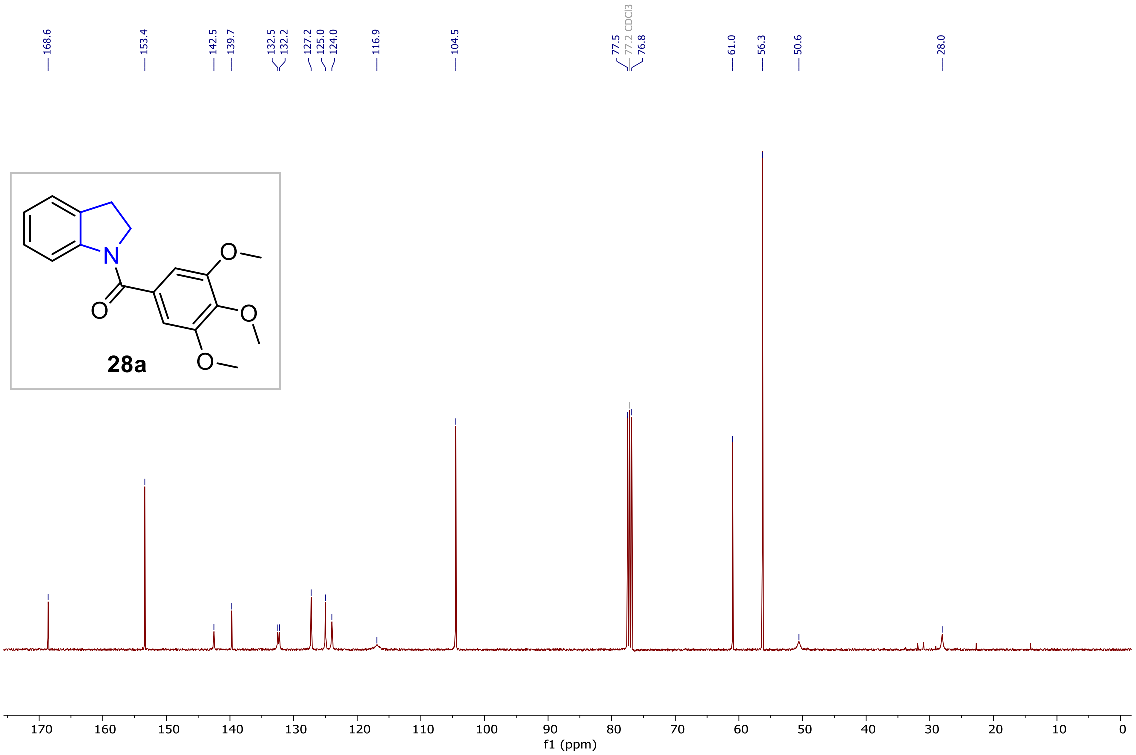

Figure S54. $^{13}C\{^{1}H\}$ (101 MHz, $CDCl_3$) NMR spectrum of indolin-1-yl(3,4,5-trimethoxyphenyl)methanone (**28a**).

10. Characterization of 17a, 17a[D], 17a' and 17a'[D]

In this section, compounds **17a, 17a[D], 17a', and 17a'[D]** were thoroughly investigated using various NMR techniques and HR-MS.

For the NMR description, the following abbreviations were used to facilitate peak assignments:

Arom.-Ring A/B = aromatic ring A or B

Cq = quaternary carbon atom

br. = broad

The numbering of the molecule was assigned according to the chemical shift of the respective carbon atom.

Additionally, HR-MS was used to determine the major isotopomers in deuterated mixtures.

2-(3,4-Dimethoxyphenethyl)-1,2,3,4-tetrahydroquinoline (17a). ^{1}**H NMR** (500 MHz, CD_2Cl_2)

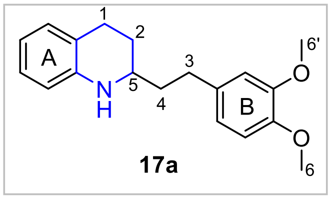

17a

$\underline{\delta}$ (ppm): 6.94-6.91 (m, 2H, CH-arom.-A), 6.82-6.81 (m, 1H, CH-arom.-B), 6.79-6.76 (m, 2H, CH-arom.-B), 6.57 (td, J = 7.4, 1.2 Hz, 1H, CH-arom.-A), 6.45-6.43 (m, 1H, CH-arom.-A), 3.83 (s, 3H, H-6'), 3.81 (s, 3H, H-6), 3.30 (dtd, J = 9.4, 6.3, 3.0 Hz, 1H, H-5), 2.84-2.74 (m, 2H, H-1), 2.72-2.68 (m, 2H, H-3), 2.03-1.98 (m, 1H, H-2), 1.83-1.79 (m, 2H, H-4), 1.69-1.62 (m, 1H, H-2) . ^{13}**C{^{1}H} NMR** (126 MHz, CD_2Cl_2) $\underline{\delta}$ (ppm): 149.5 (Cq, arom.-B), 147.8 (Cq, arom. B), 145.2 (Cq, arom. A), 135.1 (Cq, arom. B), 129.5 (CH, arom.-A), 126.9 (CH, arom.-A), 121.6 (Cq, arom. A), 120.5 (CH, arom.-B), 117.1(CH, arom.-A), 114.3 (CH, arom.-A), 112.3 (CH, arom.-B), 112.0 (CH, arom.-B), 56.2 (CH_3, C-6), 56.1 (CH_3, C-6'), 51.6 (CH, C-5), 38.9 (CH_2, C-4), 32.1 (CH_2, C-3), 28.4 (CH_2, C-2), 26.7 (CH_2, C-1).

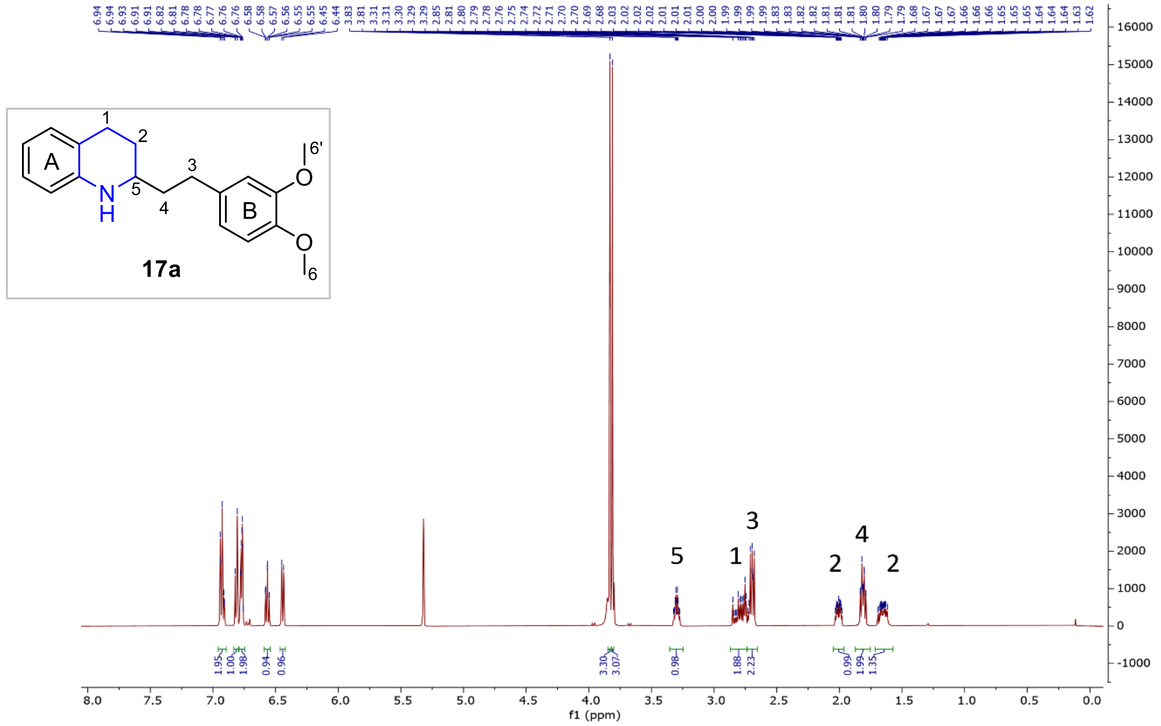

Figure S55. ^{1}H NMR (500 MHz, CD_2Cl_2) spectrum of 2-(3,4-dimethoxyphenethyl)-1,2,3,4-tetrahydroquinoline (**17a**).

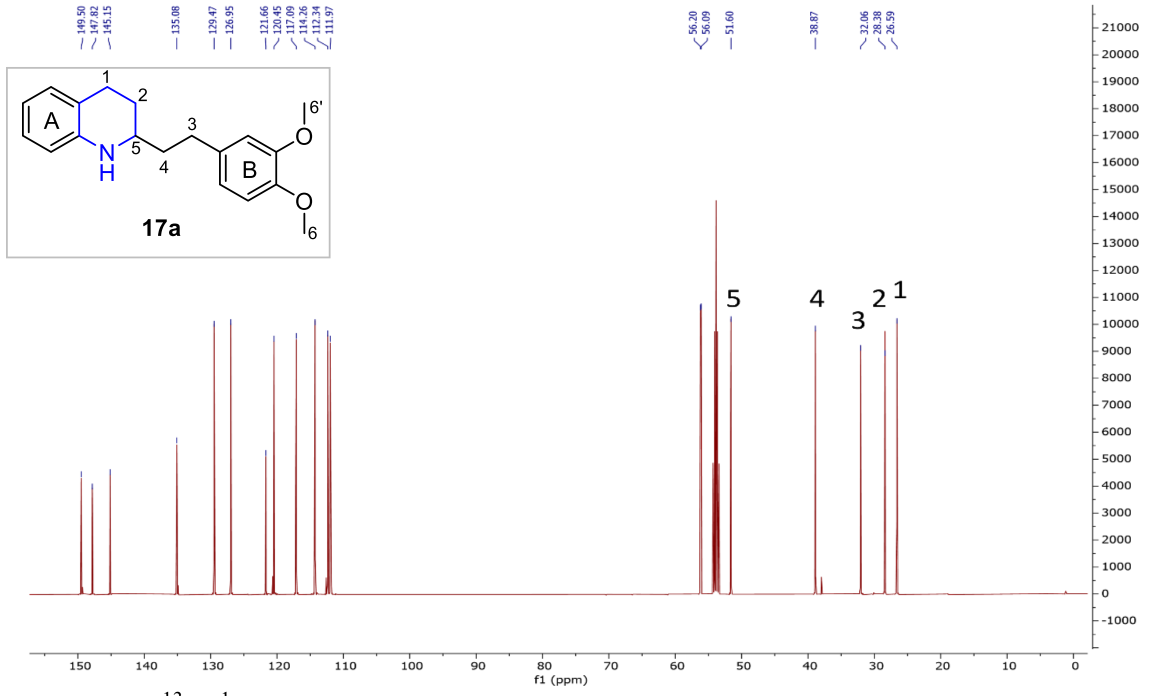

Figure S56. $^{13}C\{^{1}H\}$ (126 MHz, CD_2Cl_2) NMR spectrum of 2-(3,4-dimethoxyphenethyl)-1,2,3,4-tetrahydroquinoline (**17a**).

2-(3,4-Dimethoxyphenethyl)-1,2,3,4-tetrahydroquinoline[D] (17[D]) (mixture of isotopomers).

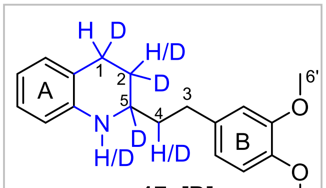

17a[D]

^{1}H NMR (500 MHz, CD_2Cl_2) δ (ppm): 6.93-6.90 (m, 2H, CH-arom.-A), 6.81-6.80 (m, 1H, CH-arom.-B), 6.78-6.75 (m, 2H, CH-arom.-B), 6.55 (td, J = 7.4, 1.2 Hz, 1H, CH-arom.-A), 6.44-6.43 (m, 1H, CH-arom.-A), 3.82 (s, 3H, H-6'), 3.80 (s, 3H, H-6), 3.29-3.27 (m, 0.07H, D-5), 2.81-2.67 (m, 3H, H/D-1, H-3), 1.97-1.96 (m, 0.19H, H/D-2), 1.84-1.75 (m, 1.5H, H/D-4), 1.62-1.60 (m, 0.42H, H/D-2).

^{2}H NMR (77 MHz, CD_2Cl_2) δ (ppm): 3.27 (br. D-5), 2.75 (br, H/D-1), 1.97(br. H/D-2), 1.79 (br. H/D-4), 1.62 (br. H/D-2).

^{13}C{^{1}H} NMR (126 MHz, CD_2Cl_2) δ (ppm): 149.5(Cq, arom.-B), 147.8 (Cq, arom. B), 145.2 (Cq, arom. A), 135.1(Cq, arom. B), 129.5 (CH, arom.-A), 129.5 (CH, arom.-A), 127.0 (CH, arom.-A), 121.6, (Cq, arom. A), 120.5 (CH, arom.-B), 117.1(CH, arom.-A), 114.2 (CH, arom.-A), 112.3 (CH, arom.-B), 112.0 (CH, arom.-B), 56.2 (CH_3, C-6), 56.1(CH_3, C-6'), 51.5-50.7 (m, CD, C-5), 38.8-38.0 (m, CH/D, C-4), 32.1-31.9 (m, CH_2, C-3), 28.2-27.1 (m, CDH/D, C-2), 26.3-25.8 (m, CH/D, C-1).

HR-MS: calculated for $C_{19}H_{18}N_1O_2D_5$ (as selected isotopomer to be analyzed) = 302.203714;(m/z), found: 302.204250. The ratio of the corresponding isotopomeres was determined by HR-MS by using the undeuterated molecule as reference: [D_1] = 0.1%, [D_2] = 2.3%, [D_3] =26.6%, [D_4] = 32.4%, [D_5] =27.1%, [D_6] = 11.4, [D_7] = 0.1 (± 0.1%).

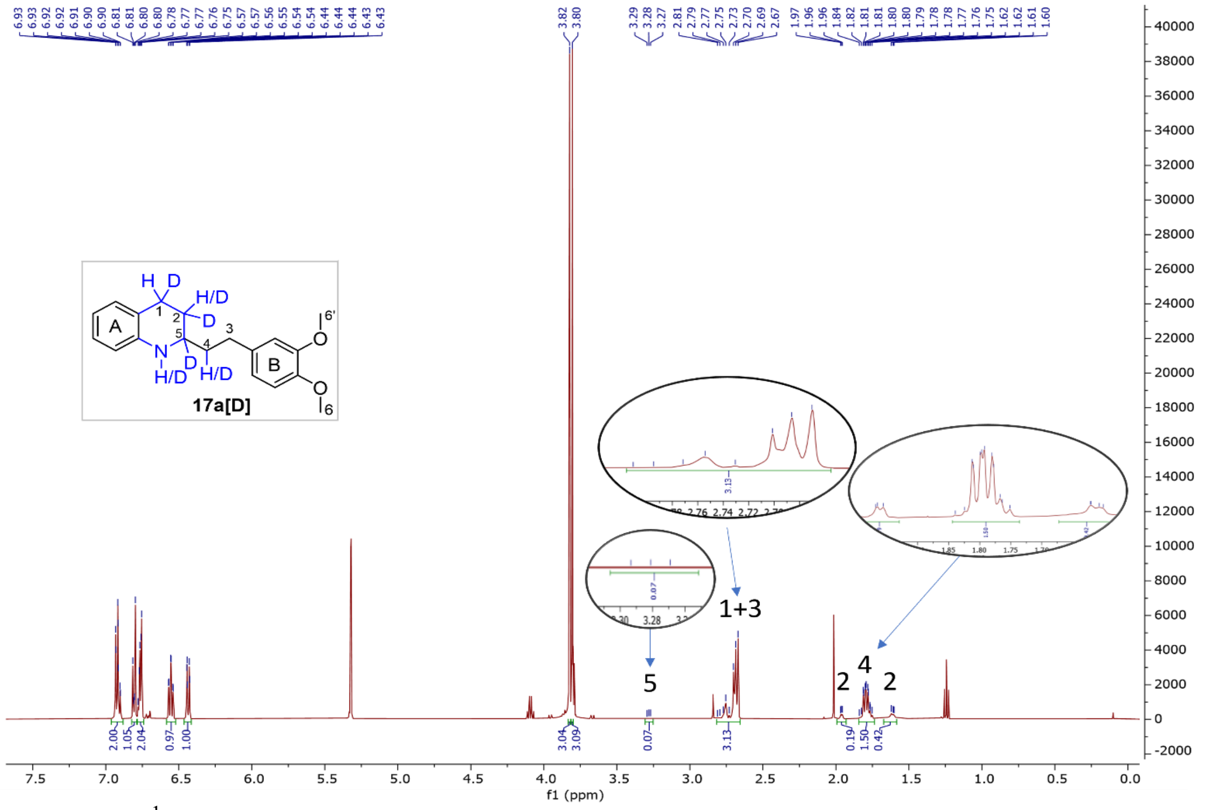

Figure S57. ^{1}H NMR (500 MHz, CD_2Cl_2) spectrum of 2-(3,4-dimethoxyphenethyl)-1,2,3,4-tetrahydroquinoline[D] (**17a[D]**).

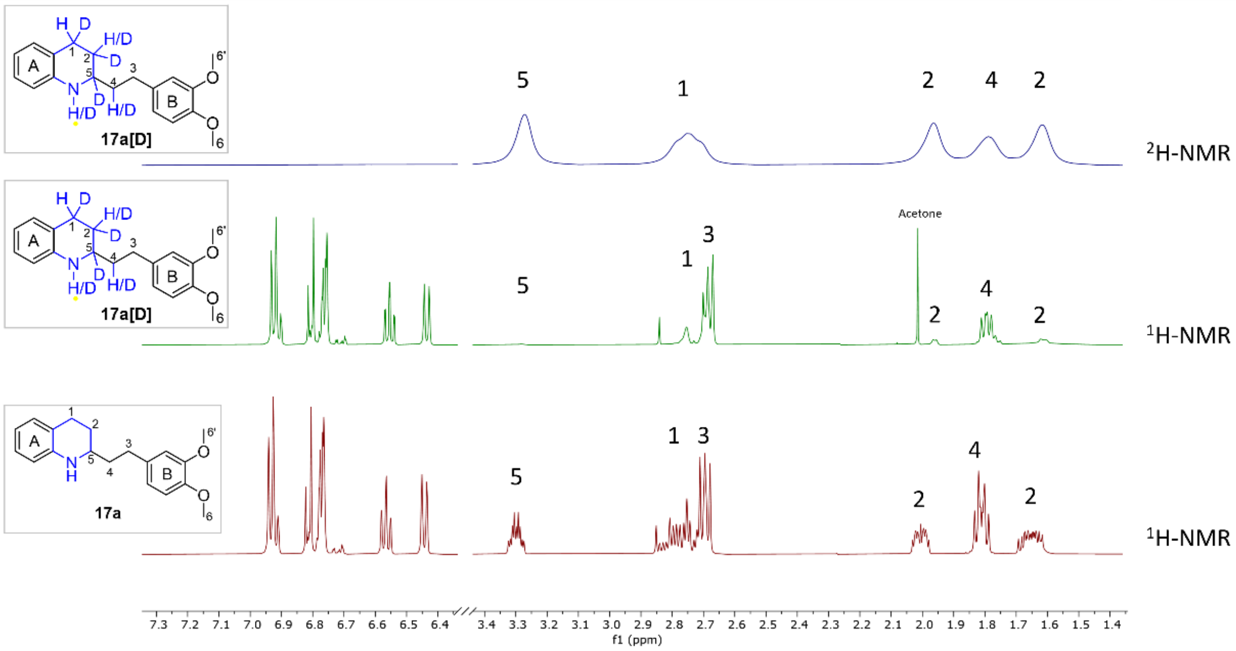

Figure S58. Top: ^{2}H-NMR of **17a[D]**, middle: ^{1}H-NMR of **17a[D]**, bottom ^{1}H-NMR of **17a.** (in CD_2Cl_2).

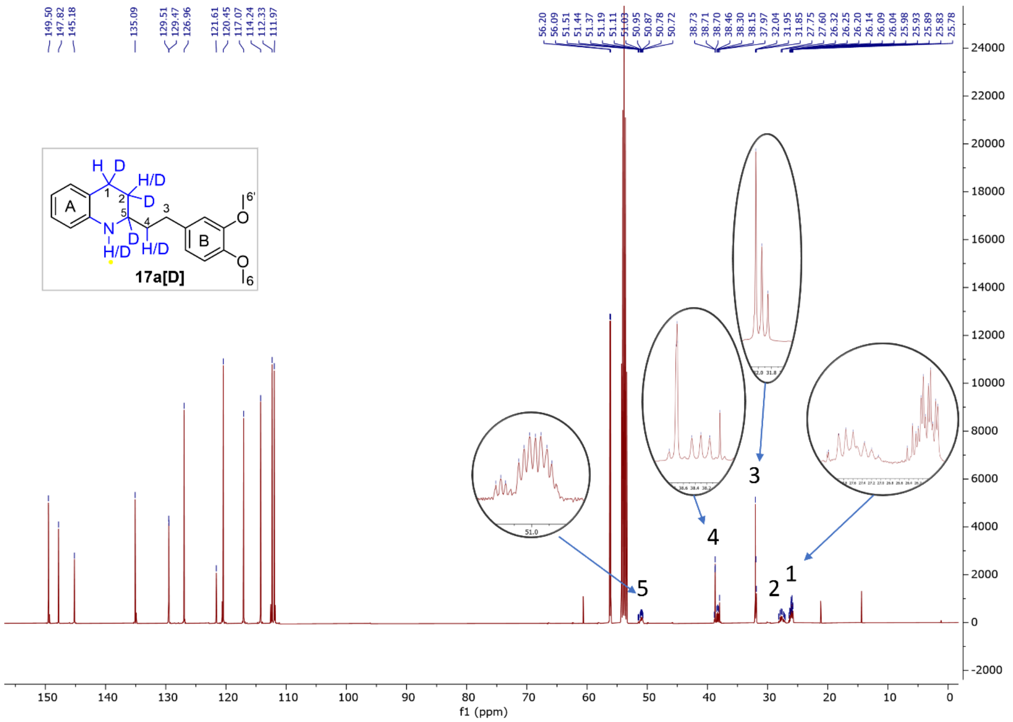

Figure S59. $^{13}C\{^{1}H\}$ (126 MHz, CD_2Cl_2) NMR spectrum of 2-(3,4-dimethoxyphenethyl)-1,2,3,4-tetrahydroquinoline[D] (**17a[D]**).

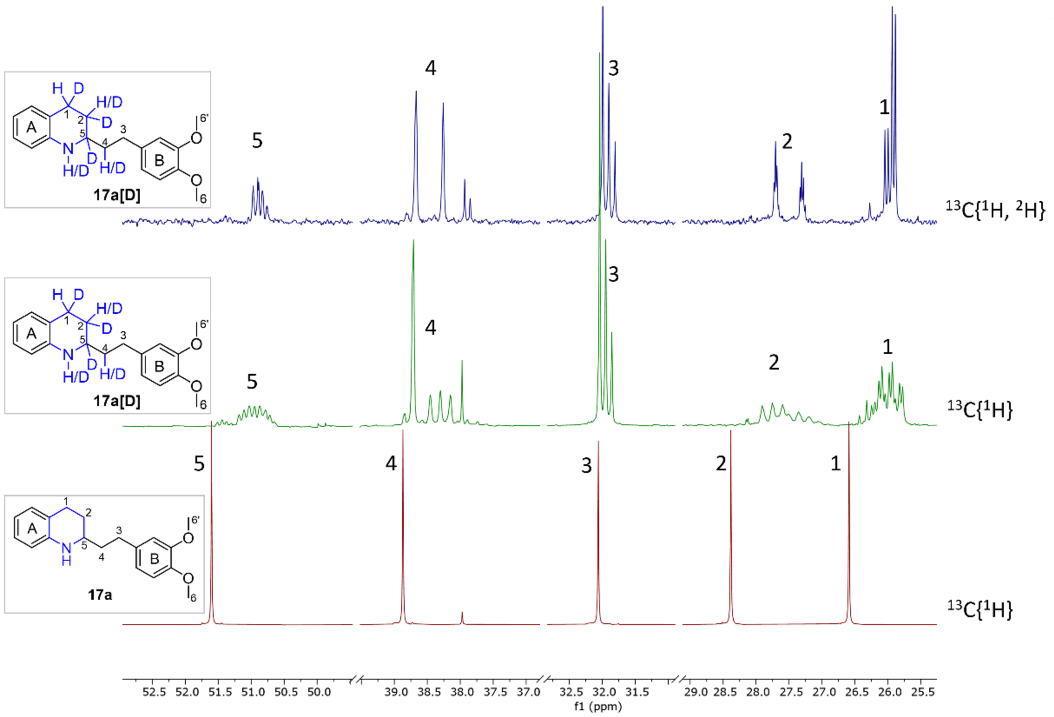

Figure S60. Top: $^{13}C\{^{1}H,^{2}H\}$-NMR of **17a[D]**, middle: $^{13}C\{^{1}H\}$-NMR of **17a[D]**, bottom $^{13}C\{^{1}H\}$-NMR of **17a**. (in CD_2Cl_2)

2-(3,4-Dimethoxyphenethyl)-1-methyl-1,2,3,4-tetrahydroquinoline (17a').

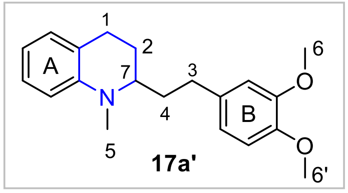

[1]**H NMR** (400 MHz, CD_2Cl_2) δ: 7.04-7.01 (m, 1H, arom.-A), 6.96-6.94 (m, 1H, arom.-A), 6.80-6.78 (m, 1H, arom.-B), 6.75-6.73 (m, 2H, arom.-B), 6.54 (td, *J* = 7.3, 1.2 Hz, 1H, arom.-A), 6.51-6.50 (m, 1H, arom.-A) 3.81 (s, 3H, H-6), 3.80 (s, 3H, H-6'), 3.29 (dq, *J* = 8.5, 4.2 Hz, 1H, H-7), 2.91 (s, 3H, H-5), 2.88-2.81(m, 1H, H-1), 2.70-2.64 (m, 2H, H-1, H-3), 2.56-2.50 (m, 1H, H-3), 2.01-1.96 (m, 1H, H-2), 1.94-1.86 (m, 2H, H-2, H-4), 1.75-1.67 (m, 1H, H-4). [13]**C{^{1}H} NMR** (126 MHz, CD_2Cl_2) δ: 149.5 (Cq, arom.-B), 147.7 (Cq. arom.-B), 145.8 (Cq, arom.-A), 135.2 (Cq, arom.-B), 128.9 (CH-arom.-A), 127.4 (CH-arom.-A), 122.2 (Cq, arom.-A), 120.4 (CH-arom.-B), 115.6 (CH-arom.-A), 112.3 (CH-arom.-B), 111.9 (CH-arom.-B), 110.8 (CH-arom.-A), 58.8 (CH, C-7), 56.2 (CH_3, C6'), 56.1 (CH_3, C6), 38.2 (CH_3, C-5), 33.4 (CH_2, C-4), 32.2 (CH_2, C-3), 24.8 (CH_2, C-2), 23.9 (CH_2, C-1).

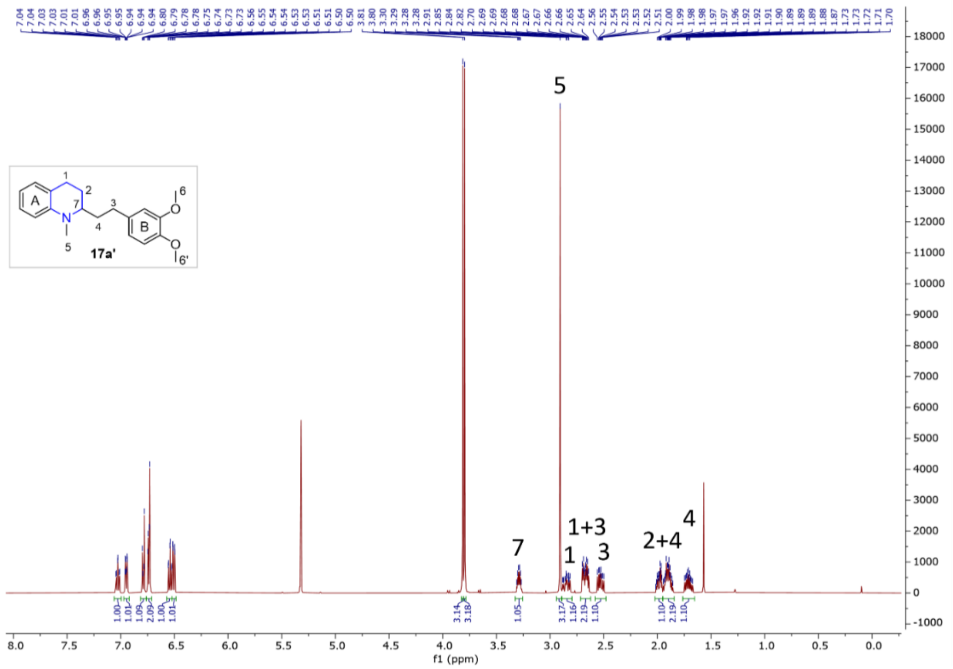

Figure S61. ^{1}H NMR (500 MHz, CD_2Cl_2) spectrum of 2-(3,4-dimethoxyphenethyl)-1-methyl-1,2,3,4-tetrahydroquinoline (**17a’**).

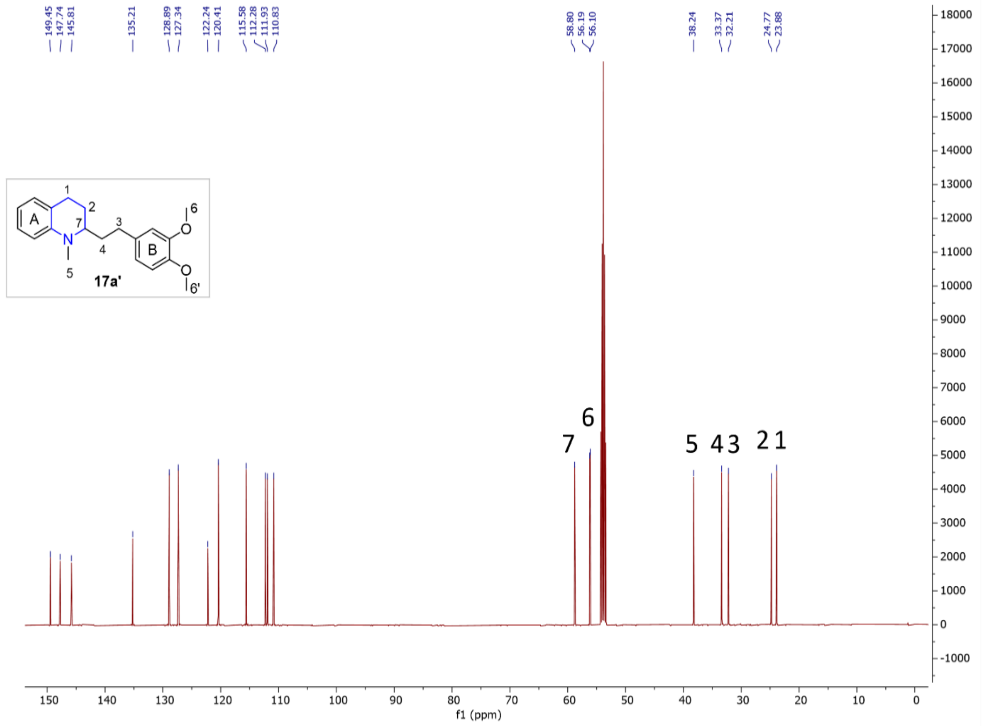

Figure S62. $^{13}C\{^{1}H\}$(126 MHz, CD_2Cl_2) NMR spectrum of 2-(3,4-dimethoxyphenethyl)-1-methyl-1,2,3,4-tetrahydroquinoline (**17a’**).

2-(3,4-Dimethoxyphenethyl)-1-methyl-1,2,3,4 tetrahydroquinoline[D] (mixture of isotopomers) (17a'[D]).

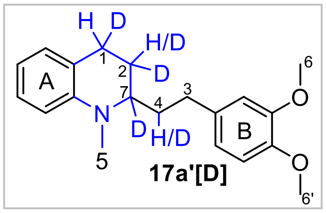

^{1}**H NMR** (MHz, CD_2Cl_2) δ: 7.04-7.00 (m, 1H, arom.-A), 6.95-6.93 (m, 1H, arom.-A), 6.79-6.78 (m, 1H, arom.-B), 6.74-6.73 (m, 2H, arom.-B), 6.54 (td, J = 7.3, 1.1 Hz, 1H, arom.-A), 6.51-6.49 (m, 1H, arom.-A) 3.81 (s, 3H, H-6), 3.79 (s, 3H, H-6'), 3.29-3.26 (m, 0.1H, D-7), 2.90 (s, 3H, H-5), 2.84-2.63 (m, 2H, H-1, H-3), 2.55-2.49 (m, 1H, H-3), 1.95-1.84 (m, 1.1H, H-2, H-4), 1.72-1.66 (m, 0.8 H, H-4).

^{2}**H NMR** (77 MHz, CD_2Cl_2) δ: 3.27 (br., D-7), 2.83 (br., H/D-1), 2.66 (br., H/D-1), 1.94-1.86 (m, br., H/D-2,4), 1.69 (br., H/D-4).

^{13}**C{^{1}H} NMR** (126 MHz, CD_2Cl_2) δ: 149.5 (Cq, arom.-B), 147.7(Cq. arom.-B), 145.8 (Cq, arom.-A), 135.2 (Cq, arom.-B), 128.9 (CH-arom.-A), 127.4 (CH-arom.-A), 122.2 (Cq, arom.-A), 120.4 (CH-arom.-B), 115.6 (CH-arom.-A), 112.3 (CH-arom.-B), 111.9 (CH-arom.-B), 110.8 (CH-arom.-A), 58.7-57.9 (m, CD, C-7), 56.2 (CH_3, C-6'), 56.1(CH_3, C-6), 38.2 (CH_3, C-5), 33.3-32.6 (m, CH/D, C-4), 32.2-32.0 (m, CH_2, C-3), 24.6-23.8 (m, CH/D, C-2), 23.7-23.1 (m, CH/D, C-1).

HR-MS: calculated for $C_{20}H_{20}N_1O_2D_5$ (as selected isotopomer to be analyzed) =316.219364 (m/z), found: 316.219720. The ratio of the corresponding isotopomeres was determined by HR-MS using the undeuterated molecule as reference: [D_1] = 1.4%, [D_2] = 3.3%, [D_3] =29.5%, [D4] = 32.7%, [D_5] =26.1%, [D_6] = 6.5 (± 2.6%).

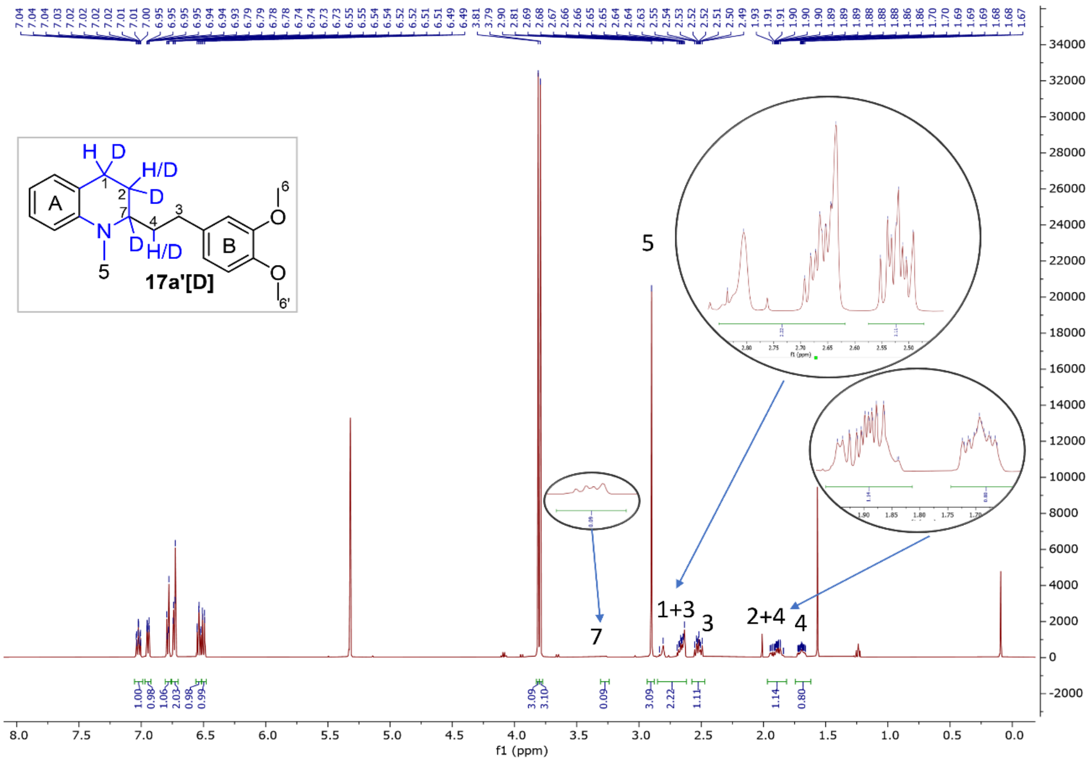

Figure S63. ^{1}H NMR (500 MHz, CD_2Cl_2) spectrum of 2-(3,4-dimethoxyphenethyl)-1-methyl-1,2,3,4-tetrahydroquinoline[D] (**17a'[D]**).

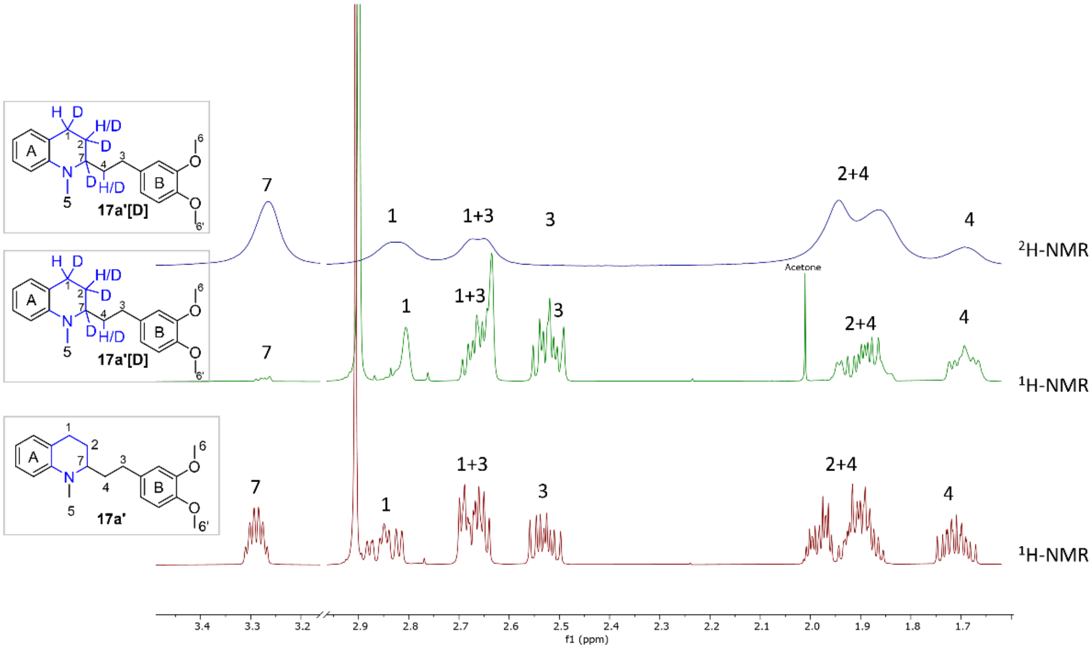

Figure S64. Top: ^{2}H-NMR of **17a'[D]**, middle: ^{1}H-NMR of **17a'[D]**, bottom ^{1}H-NMR of **17a'**. (in CD_2Cl_2)

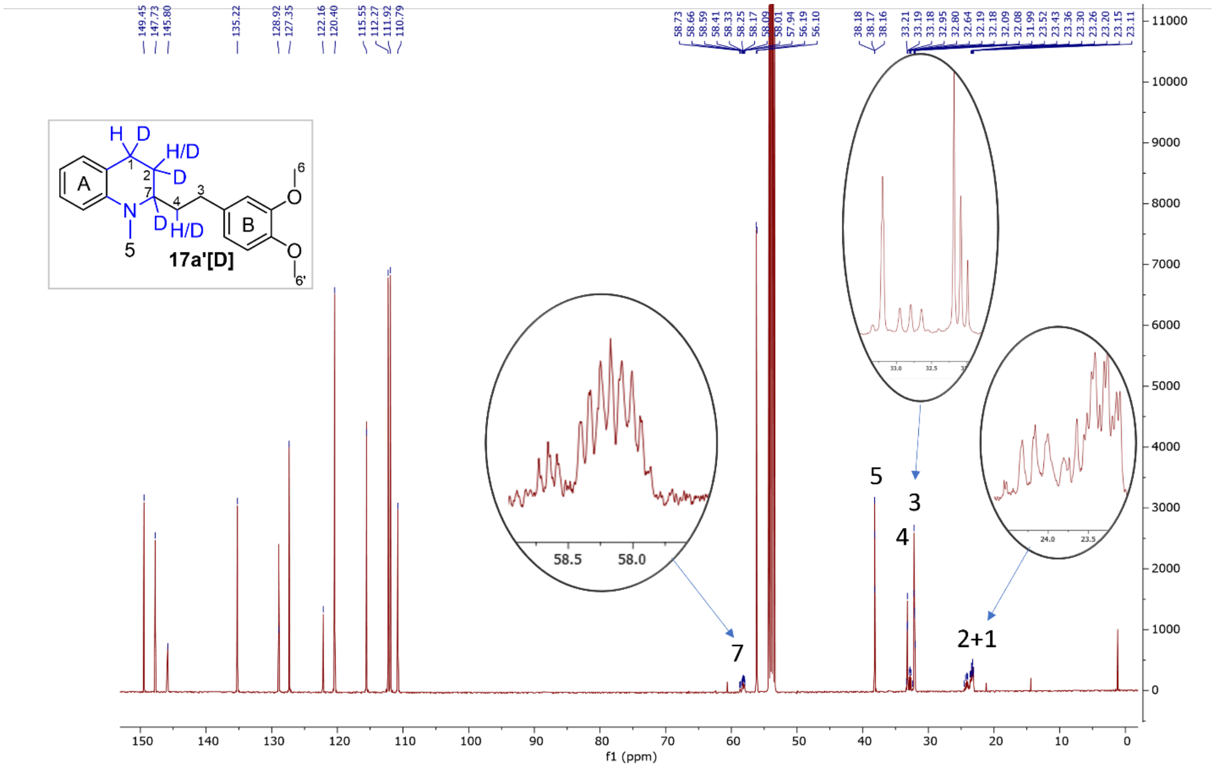

Figure S65. $^{13}C\{^1H\}$ (126 MHz, CD_2Cl_2) NMR spectrum of 2-(3,4-dimethoxyphenethyl)-1,2,3,4-tetrahydroquinoline[D] (**17a'[D]**).

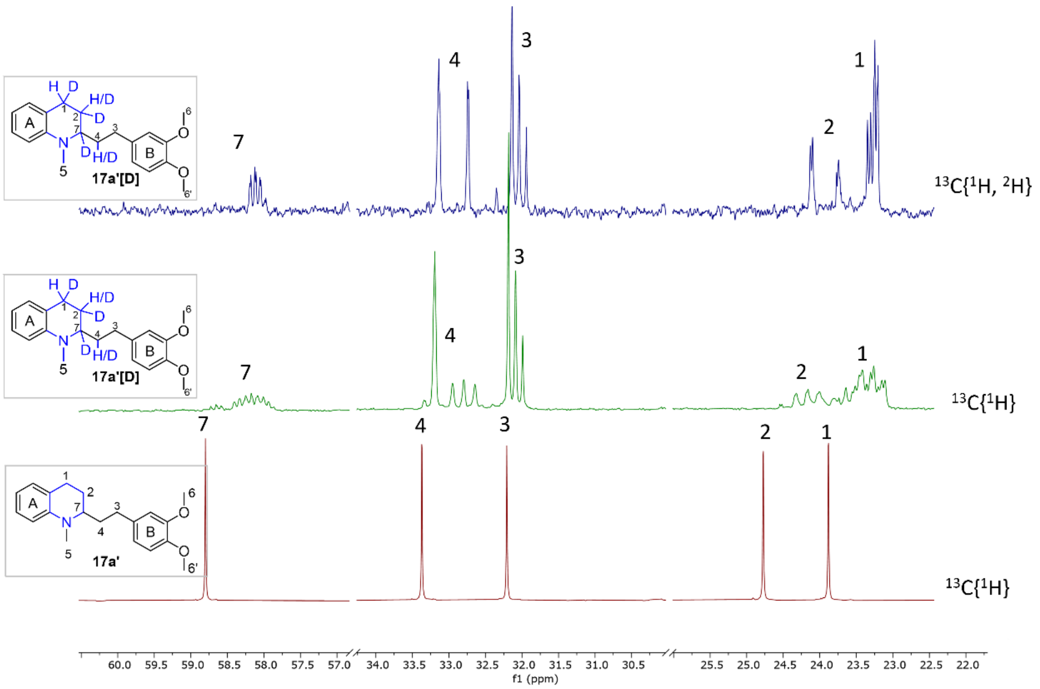

Figure S66. Top: $^{13}C\{^1H,^2H\}$-NMR of **17a'[D]**, middle: $^{13}C\{^1H\}$-NMR of **17a'[D]**, bottom $^{13}C\{^1H\}$-NMR of **17a'**. (in CD_2Cl_2)

11. References

(1) Mahun, A.; Abbrent, S.; Czernek, J.; Rohlicek, J.; Mackova, H.; Ning, W.; Konefal, R.; Brus, J.; Kobera, L. Reconstructing reliable powder patterns from spikelets (Q)CPMG NMR spectra: simplification of UWNMR crystallography analysis. *Molecules* **2021,** *26*, 6051.

(2) Massiot, D.; Fayon, F.; Capron, M.; King, I.; Le Calvé, S.; Alonso, B.; Durand, J. O.; Bujoli, B.; Gan, Z.; Hoatson, G. Modelling one- and two-dimensional solid-state NMR spectra. *Magn. Reson. Chem.* **2002,** *40*, 70-76.

(3) Fung, B. M.; Khitrin, A. K.; Ermolaev, K. An improved broadband decoupling sequence for liquid crystals and solids. *J. Magn. Reson.* **2000,** *142*, 97–101.

(4) Harris, R.; Becker, E.; de Menezes, C.; Goodfellow, R.; Granger, P. NMR nomenclature, nuclear spin properties, and conventions for chemical shifts. *Pure Appl. Chem.* **2001,** *73*, 1795–1818.

(5) Morcombe, C. R.; Zilm, K. W. Chemical shift referencing in MAS solid state NMR. *J. Magn. Reson.* **2003,** *162*, 479-86.

(6) Rankin, A. G. M.; Webb, P. B.; Dawson, D. M.; Viger-Gravel, J.; Walder, B. J.; Emsley, L.; Ashbrook, S. E. Determining the surface structure of silicated alumina catalysts via isotopic enrichment and dynamic nuclear polarization surface-enhanced NMR spectroscopy. *J. Phys. Chem. C* **2017,** *121*, 22977-22984.

(7) Kupce, E.; Freeman, R. Adiabatic pulses for wideband inversion and broadband decoupling. *J. Magn. Reson. Series A* **1995,** *115*, 273–276.

(8) Eichele, K.; Wasylishen, R. E. ^{31}P NMR study of powder and single-crystal samples of ammonium dihydrogen phosphate: effect of homonuclear dipolar coupling. *J. Phys. Chem.* **1994,** *98*, 3108–3113.

(9) Welter, E.; Chernikov, R.; Herrmann, M.; Nemausat, R. A beamline for bulk sample x-ray absorption spectroscopy at the high brilliance storage ring PETRA III. *AIP Conf. Proc.* **2019,** *2054*.

(10) S. Belin; Briois, V.; Traverse, A.; Idir, M.; Moreno, T.; Ribbens, M. SAMBA a new beamline at SOLEIL for x-ray absorption spectroscopy in the 4–40 keV energy range. *Phys. Scr.* **2005,** *T115*, 980-983.

(11) Fonda, E.; Rochet, A.; Ribbens, M.; Barthe, L.; Belin, S.; Briois, V. The SAMBA quick-EXAFS monochromator: XAS with edge jumping. *J. Synchrotron Radiat.* **2012,** *19*, 417-424.

(12) Ravel, B.; Newville, M. ATHENA, ARTEMIS, HEPHAESTUS: data analysis for X-ray absorption spectroscopy using IFEFFIT. *J. Synchrotron Radiat.* **2005,** *12*, 537-541.
(13) Liu, C.; Wang, M.; Liu, S.; Wang, Y.; Peng, Y.; Lan, Y.; Liu, Q. Manganese-catalyzed asymmetric hydrogenation of quinolines enabled by π–π interaction. *Angew. Chem. Int. Ed.* **2021,** *60*, 5108-5113.
(14) Kwan, M. H. T.; Breen, J.; Bowden, M.; Conway, L.; Crossley, B.; Jones, M. F.; Munday, R.; Pokar, N. P. B.; Screen, T.; Blacker, A. J. Continuous flow chiral amine racemization applied to continuously recirculating dynamic diastereomeric crystallizations. *J. Org. Chem.* **2021,** *86*, 2458-2473.
(15) Liou, J.-P.; Wu, C.-Y.; Hsieh, H.-P.; Chang, C.-Y.; Chen, C.-M.; Kuo, C.-C.; Chang, J.-Y. 4- and 5-Aroylindoles as Novel Classes of Potent Antitubulin Agents. *J. Med. Chem.* **2007,** *50*, 4548-4552.
(16) Sahoo, B.; Kreyenschulte, C.; Agostini, G.; Lund, H.; Bachmann, S.; Scalone, M.; Junge, K.; Beller, M. A robust iron catalyst for the selective hydrogenation of substituted (iso)quinolones. *Chem. Sci.* **2018,** *9*, 8134-8141.
(17) Bhattacharyya, D.; Nandi, S.; Adhikari, P.; Sarmah, B. K.; Konwar, M.; Das, A. Boric acid catalyzed chemoselective reduction of quinolines. *Org. Biomol. Chem.* **2020,** *18*, 1214-1220.
(18) Louis Anandaraj, S. J.; Kang, L.; DeBeer, S.; Bordet, A.; Leitner, W. Catalytic hydrogenation of CO_2 to formate using ruthenium nanoparticles immobilized on supported ionic liquid phases. *Small* **2023,** *19*, e2206806.
(19) Sodreau, A.; Zahedi, H. G.; Dervisoglu, R.; Kang, L.; Menten, J.; Zenner, J.; Terefenko, N.; DeBeer, S.; Wiegand, T.; Bordet, A.; Leitner, W. A simple and versatile approach for the low-temperature synthesis of transition metal phosphide nanoparticles from metal chloride complexes and $P(SiMe_3)_3$. *Adv. Mater.* **2023,** *35*, e2306621.
(20) Zhang, Y.; El Sayed, S.; Kang, L.; Sanger, M.; Wiegand, T.; Jessop, P. G.; DeBeer, S.; Bordet, A.; Leitner, W. Adaptive catalysts for the selective hydrogenation of bicyclic heteroaromatics using ruthenium nanoparticles on a CO_2-responsive support. *Angew. Chem. Int. Ed.* **2023,** *62*, e202311427.
(21) Shao, F.; Yao, Z.; Gao, Y.; Zhou, Q.; Bao, Z.; Zhuang, G.; Zhong, X.; Wu, C.; Wei, Z.; Wang, J. Geometric and electronic effects on the performance of a bifunctional Ru2P catalyst in the

hydrogenation and acceptorless dehydrogenation of N-heteroarenes. *Chin. J. Catal.* **2021,** *42*, 1185-1194.

(22) Sun, B.; Khan, F.-A.; Vallat, A.; Süss-Fink, G. NanoRu@hectorite: A heterogeneous catalyst with switchable selectivity for the hydrogenation of quinoline. *Appl. Catal. A: Gen.* **2013,** *467*, 310-314.

(23) Niu, M.; Wang, Y.; Chen, Q.; Du, D.; Jiang, J.; Jin, Z. Highly efficient and recyclable rhodium nanoparticle catalysts for hydrogenation of quinoline and its derivatives. *Catal. Sci. Technol* **2015,** *5*, 4746-4749.

(24) Bai, L.; Wang, X.; Chen, Q.; Ye, Y.; Zheng, H.; Guo, J.; Yin, Y.; Gao, C. Explaining the Size Dependence in Platinum-Nanoparticle-Catalyzed Hydrogenation Reactions. *Angew. Chem. Int. Ed.* **2016,** *55*, 15656-15661.

(25) Chen, F.; Surkus, A.-E.; He, L.; Pohl, M.-M.; Radnik, J.; Topf, C.; Junge, K.; Beller, M. Selective catalytic hydrogenation of heteroarenes with N-graphene-modified cobalt nanoparticles (Co_3O_4-Co/NGr@α-Al_2O_3). *J. Am. Chem. Soc.* **2015,** *137*, 11718-11724.

(26) Schmiedbauer, F.; Monkowius, U.; Schwarzinger, C.; Mullegger, S.; Bartling, S.; Rockstroh, N.; Topf, C. Hydrogenation of Quinolines and Aldehydes Catalyzed by a Pyrolyzed, Augmented Cobalt-Salen Complex. *ACS Omega* **2025,** *10*, 36455-36465.

(27) Sheldon, R. A. Metrics of green chemistry and sustainability: past, present, and future. *ACS Sustain. Chem. Eng.* **2017,** *6*, 32-48.

(28) Su, Y.; Jin, X.; Su, J.; Feng, Y.; Wang, Q.; Zhang, Z.; Tian, H.; Ma, X. Radical Afterglow Emission Harnessed by Doping N,N'-Diaryl-5,10-Dihydrophenazines to Epoxy Resins. *Adv. Opt. Mater.* **2023,** *11*.

(29) Zhang, C.; Zhang, H.; Dong, L. 2-Arylphthalazine-1,4-diones as Nitrene Precursors for the Synthesis of Phenazine Derivatives. *Org. Lett.* **2025,** *27*, 7903-7907.
